# Beyond the Virial Expansion: Microscopic Origins of Partial Molar Volumes in LiCl Solutions

**DOI:** 10.1021/jacs.6c08519

**Published:** 2026-09-24

**Authors:** Chun-Ting Lin, Diganta Dasgupta, Tinglu Yang, Cesare Malosso, Giulia Sormani, Colin Egan, Giovanni Bussi, Ali Hassanali, Paul S. Cremer

**Affiliations:** † Department of Chemistry, 311285 The Pennsylvania State University, University Park, Pennsylvania 16802, United States; ‡ Condensed Matter and Statistical Physics, 18473 The Abdus Salam International Centre for Theoretical Physics (ICTP), Trieste 34151, Italy; § 19040 Scuola Internazionale Superiore di Studi Avanzati (SISSA), Trieste 34136, Italy; ∥ Laboratory of Computational Science and Modelling, 27218 IMX, École Polytechnique Fédérale de Lausanne, Lausanne CH-1015, Switzerland; ⊥ Initiative for Computational Catalysis, 543881 Flatiron Institute, New York, New York 10010, United States

## Abstract

Although electrolyte density measurements have been reported for over a century, employing them to determine accurate partial molar volume (PMV) profiles as a function of salt concentration has remained elusive. Obtaining such curves requires precise density measurements combined with a proper treatment of the associated virial expansion. In this work, we derive PMV profiles for aqueous LiCl solutions. The resulting data enable the development of highly accurate force fields for Li^+^ and Cl^–^, revealing a clear progression from isolated ions to ion pairs and ultimately to higher-order chain and ring structures. Because ion clustering emerges from nonlocal interactions, it cannot be easily mapped onto specific virial terms. Instead, a direct structural and volumetric interpretation can be achieved by partitioning molecular dynamics (MD) simulation snapshots into three-dimensional polyhedral regions associated with individual salt ions and water molecules. The corresponding ionic and water volumes from this treatment quantitatively reproduce the experimental PMV curve, showing that the PMV for salt increases (while water decreases) up to 6.7 M. Above this concentration, the direction reverses as three- and four-body interactions become prominent. Complementary multivariate curve resolution (MCR) Raman spectroscopy and density functional theory (DFT) calculations elucidate the molecular-level details of water electrostriction, which also persists up to 6.7 M. Significantly, the PMV data can be correlated with key thermodynamic properties, including osmotic coefficients and the eutectic point. The procedures established here provide a general framework for modeling electrolyte solutions and enable the development of a new generation of accurate force fields for aqueous ions.

## Graphical abstract

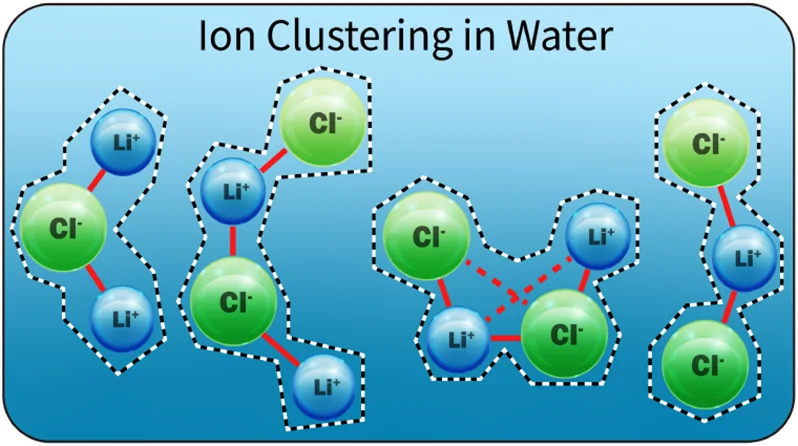

## 1 Introduction

Ion-specific effects in aqueous electrolyte solutions have been actively explored since the foundational studies of Franz Hofmeister^1^ in the 1880s. Their influence is pervasive across the physical, chemical, biological, and material sciences.^2–6^ For example, the specific identity of both cations and anions influences transport properties in batteries and fuel cells,^7,8^ affects corrosion rates,^9,10^ solvent extraction efficiency,^11^ as well as protein and drug molecule crystallization.^5,12,13^ Ion-specific interactions are also at the heart of technologies aimed at optimizing wastewater treatment^14^ and carbon capture.^15^ Moreover, cation specificity is central to the behavior of living organisms. For example, K^+^ and Mg^2+^ accumulate in the cytosol, while Na^+^ and Ca^2+^ are highly abundant in the extracellular matrix, a crucial factor for controlling the cell membrane potential.^16^ While Li^+^ is less frequently encountered in biological systems, its clinical utility in treating bipolar disorder is well established. Additionally, endogenous trace Li^+^ levels within cerebrospinal fluid are vital to maintaining cognitive function.^17,18^

To understand electrolyte properties in aqueous solutions, it is necessary to anchor the behavior of dissolved salts to fundamental solution properties, such as density. Indeed, the density of an aqueous solution containing ions has historically been used as a key property to parametrize and validate interaction potentials in all-atom molecular dynamics simulations.^19–31^ Curiously, however, density data from the literature yield substantially different partial molar volume (PMV) curves as a function of salt concentration. The challenge is that the PMV for salt $\left(V_{m}^{\mathrm{salt}}\right)$ and water $\left(V_{m}^{H_{2}O}\right)$ need to be obtained from the derivative of the density with respect to salt concentration. This requires high-quality measurements, which are difficult to make with classical weight and volume measurements, as well as only moderately pure chemicals.

Beyond the challenges of obtaining accurate experimental data, interpreting thermodynamic behavior introduces an additional layer of complexity. Building on the statistical foundations established by McMillan and Mayer,^32,33^ the virial expansion has become the standard framework for describing how thermodynamic properties of electrolyte solutions, such as the PMV, depend on concentration (eq 1):

1 $$V_{m}=B_{0}+B_{1}\left[c\right]+B_{2}{[c]}^{2}+B_{3}{[c]}^{3}+B_{4}{[c]}^{4}+.....$$

In this equation, B_0_ represents the partial molar volume at infinite salt dilution, while the higher order terms, B_*n*_, with *n* > 0, serve as correction factors that describe how the PMV evolves with increasing salt concentration. From a statistical mechanical perspective, the virial coefficients can be formally related to contributions from one-body, two-body, up to n-body correlations.^32,34^ The main conceptual difficulty lies in their microscopic interpretation. Although the PMV is a well-defined thermodynamic property, mapping the B_*n*_ coefficients onto physically meaningful local volumes associated with specific configurations of ions and water molecules is not straightforward. This challenge becomes particularly pronounced at higher electrolyte concentrations, where many-body correlations, collective behavior, and cooperative effects play an increasingly significant role.

Figure 1 illustrates the evolution of an electrolyte solution upon the introduction of salt, starting from pure water. A variety of multibody effects directly include the response of the surrounding solvent molecules as well as the ions. The question arises as to how such n-body interactions contribute to the PMV. Moreover, one can ask how the structure of a 2-body or 3-body effect is defined at the molecular level. These questions currently remain open in electrolyte theory but have profound implications for a variety of thermodynamic properties such as viscosity, conductivity, surface tension, and osmotic pressure, among others, that can each be fit to a power series. Molecular dynamics simulations could, in principle, provide detailed structural insight to clarify the interpretation of these parameters, but this requires interatomic potentials capable of accurately reproducing experimental PMVs, a requirement that has remained challenging to meet.

**Figure 1:**
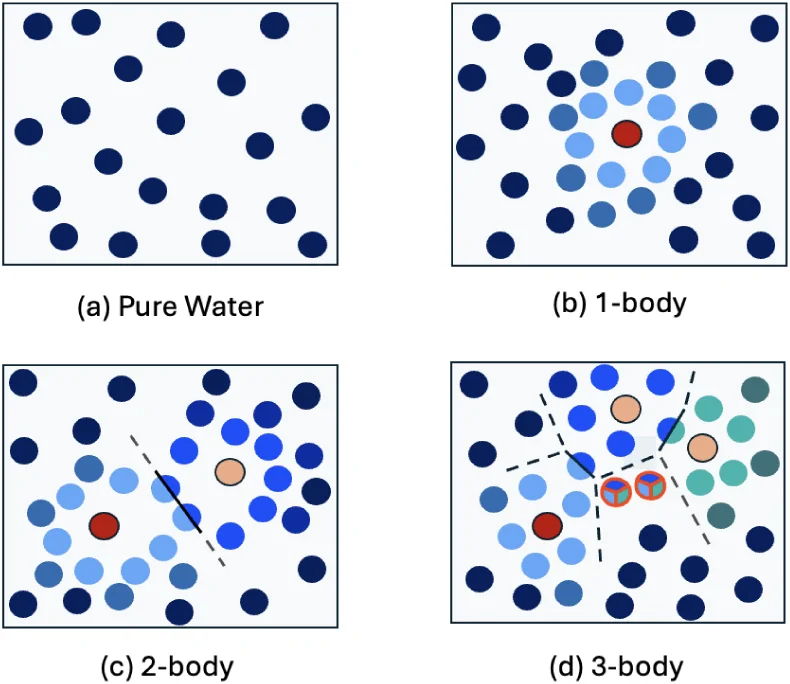
Schematic diagrams of the interactions between ions and water molecules in aqueous solutions showing a structural progression from (a) water molecules in neat water (navy blue spheres) to (b) the hydration of a single ion (red sphere), and finally to increasingly complex solvent structuring as (c) one and (d) two counterions (beige spheres) are introduced. Water molecules in the hydration shells of the ions are shaded light blue, bright blue, and light green. When a water molecule is in the hydration shell of more than one ion, multiple colors are used.

Herein, a general framework is presented for probing electrolyte solutions that combines thermodynamic measurements, vibrational spectroscopy, molecular dynamics simulations, and quantum-chemistry calculations to obtain and interpret the PMV for electrolyte solutions. The procedure begins with state-of-the-art pycnometry measurements of the density, enabling the extraction of $V_{m}^{\mathrm{salt}}$ and $V_{m}^{H_{2}O}$ as a function of salt concentration. These measurements afford aqueous electrolyte solution densities to five significant figures at ±0.03% uncertainty. Next, we conducted all-atom classical molecular dynamics (MD) simulations using the TIP4*P*/2005 ^31^ water model. This was done via an optimization procedure in which the ion force-field parameters were tuned to reproduce the experimentally measured solution density and PMV values as a function of concentration. The data harvested from the MD simulations were used to build a PMV model, bottom-up, from local Voronoi-like volumes^35–37^ associated with ions and water. Finally, the response of the hydrogen-bonding network of water was probed as a function of salt concentration using Raman spectroscopy analyzed via multivariate curve resolution (MCR).^38–40^ The OH stretch resonances were assigned by normal-mode vibrational spectra determined from density functional theory (DFT) electronic structure calculations.^41^

This synergistic experimental and computational approach was used to investigate LiCl solutions between 0.1 and 9.5 M at room temperature and pressure. Due to its small ionic radius and high charge density, Li^+^ served as a versatile model to investigate how kosmotropic cations^2,5^ perturbed the thermodynamic and dynamical properties of salt solutions. It was found that the PMV curves for salt and water displayed a maximum and minimum, respectively, near 6.7 M. Using molecular simulations from optimized potentials that nearly quantitatively reproduced the experimentally determined PMVs, we demonstrated that nonlocal ion-pairing led to the formation of chain and ring structures that were a crucial factor shaping the PMV data. By decomposing the thermodynamic parameters into molecular contributions, these results indicated that the PMV arose from a collective response involving both isolated hydrated ions and correlated species, forming nonlocal ion pairs. Vibrational Raman spectra revealed distinct populations of waters engaged in forming weaker and stronger hydrogen bonds, which DFT calculations link to the hydration of isolated chloride ions, water molecules involved in solvent-shared ion pairing, and the hydration of contact ion pairs.

## 2 Methods

We begin by briefly summarizing the essential aspects of the experimental and computational methods that were employed in this work before proceeding to the results. A more detailed description of the equipment, protocols, and procedures which were used on both the experimental and theoretical fronts can be found in the Supporting Information (SI).

### 2.1 Density Measurements

Aqueous LiCl solution density was measured at 20.0 °C using a borosilicate glass pycnometer with a calibrated volume of 53.372 ± 0.002 mL. A NANO pure water system (Barnstead) and a Milli-Q-UF-Plus water purifier (Millipore) were used to obtain 18.2 MΩ·cm water, which was, in turn, employed to prepare LiCl solutions. To begin an experiment, LiCl (ACS reagent, >99%) was baked at 580 °C for 14 h. The baked salt was then used to prepare electrolyte solutions. Impurities and particulates were removed from the nascently formed solutions by two rounds of syringe filtration. The combined uncertainty in the overall procedure for making density measurements from 0.1009 to 9.5184 M was less than ±0.0005 g/mL. The partial molar volumes were subsequently derived from the density profiles as described in the Results section. For additional details on the density measurements and extraction of the PMVs, see Section S1 in the SI.

### 2.2 Raman MCR Analysis

A home-built instrument was used to measure Raman spectra. Changes in the water structure in the OH stretch regime were observed between 3000 cm^–1^ and 3800 cm^–1^ as a function of LiCl concentration. MCR analysis was employed to deconvolute individual spectra into a linear combination of solute-correlated $\left(C_{S}S_{S}^{T}\right)$ and bulk water $\left(C_{W}S_{W}^{T}\right)$ contributions as well as residual noise (**E**), starting from an input Raman spectrum matrix (**D**). The *C*_*i*_ and $S_{i}^{T}$ terms denote the concentration profile and resolved component spectra of the Raman signal, respectively, for the solute-correlated and bulk-like water components of the spectra. Further details on the Raman measurements and analysis of the various modes are in Section S2 in the SI.

### 2.3 Computational Methods

Classical molecular dynamics (MD) simulations with empirical potentials were used to study the behavior of LiCl solutions as a function of concentration. Specifically, we focused on optimizing the Lennard-Jones (LJ) parameters for Li^+^ and Cl^–^ starting from the Joung-Cheatham^42^ ion force field. This builds on earlier studies that combined ensemble-based methods with force field refinement techniques.^43–48^ TIP4*P*/2005 ^31,49^ was chosen as the water model and left unmodified. The optimized ion parameters reproduced the experimental densities and PMVs. Next, microsecond long MD simulations over a range of concentrations from 0.5 to 10 M were conducted. The GROMACS 2023.3 ^50^ package was used for all MD simulations. Temperature and pressure were maintained via the Stochastic Velocity^51^ and Cell^52^ Rescaling recipes, respectively. The results were harvested to perform structural analyses using a combination of Python scripts. The libraries used are referenced herein.^53–57^ Raman calculations were conducted on model ion–water clusters using the ORCA^58^ package with DFT.^59–70^ All representations of molecular geometries were generated using Visual Molecular Dynamics^71^ (VMD). Unless stated otherwise, details of the computational methodologies employed, including optimization protocols and simulation parameters, are described in Section S3 in the SI.

## 3 Results

### 3.1 Density and Partial Molar Volumes

The experimental density profile for LiCl in water from 0 to 9.5 M is shown in Figure 2a (black data points). In the absence of salt, the density of pure water at 1 bar and 20 °C was 0.99818 ± 0.00008 g/mL. The density increased roughly linearly at low LiCl concentrations but began to bend downward at higher salt concentrations. Fitting the data required a virial expansion up to third order in the density (see Figure S1.1 and accompanying discussion in the SI). The Lennard-Jones parameters for LiCl in water system were then optimized to reproduce the experimental results (red data points and curve, see SI Section S3.1 for details). Next, $V_{m}^{LiCl}$ and $V_{m}^{H_{2}O}$ were derived from the experimental and simulation data in Figure 2a by using eqs 2 and 3, respectively:

2a $$V_{m}^{LiCl}=\frac{M_{LiCl}}{\rho }-\frac{1}{\rho^{2}}\left(1000+mM_{LiCl}\right)\left(\frac{\partial \rho }{\partial m}\right)$$

2b $$V_{m}^{H_{2}O}=\frac{M_{H_{2}O}}{\rho }+\left(\frac{mM_{H_{2}O}}{\rho^{2}}\right)\left(1+\frac{mM_{LiCl}}{1000}\right)\left(\frac{\partial \rho }{\partial m}\right)$$

**Figure 2:**
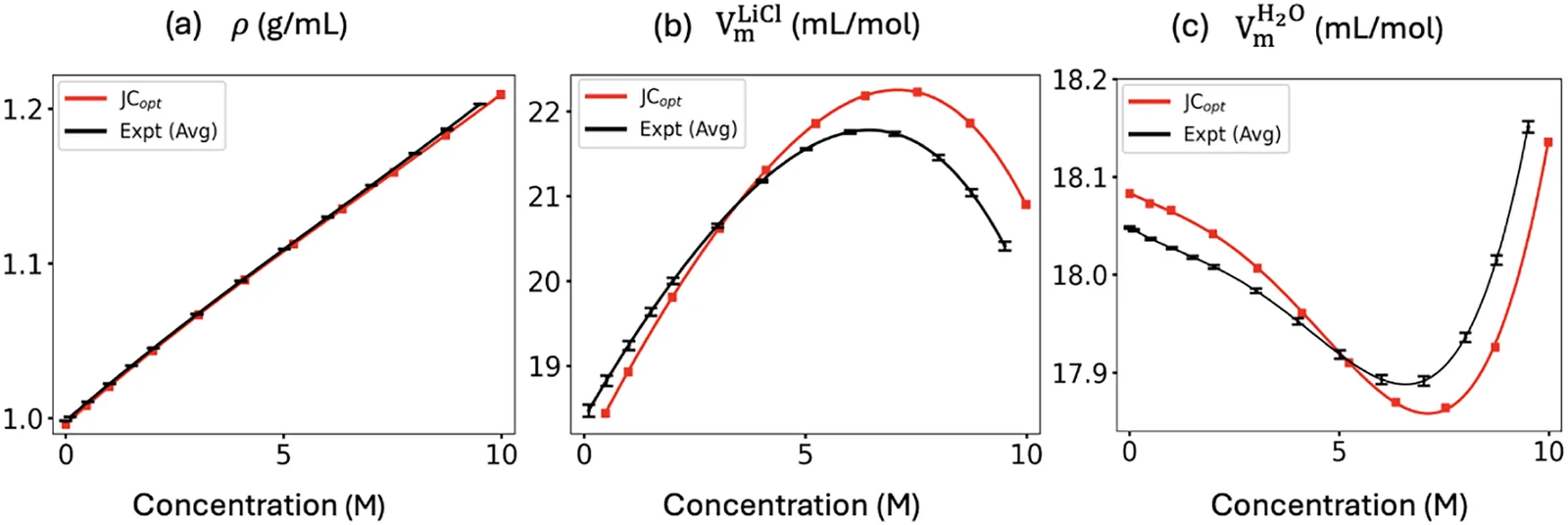
Experimental (black lines) and simulation (red lines) profiles for (a) density as well as the partial molar volumes of (b) LiCl and (c) water. The experimental data points are drawn with error bars that come from the standard deviation from three independent trials. Uncertainties in the theoretical estimates are smaller than the data points.

Here, the *M*_*i*_ parameters represent the molar mass of LiCl and water, respectively, while *m* is the molality of the solution (see Section S1.2.A in the SI for the derivation of the PMV). The term, ∂ρ/∂*m*, is the derivative of the solution density with respect to molality. Note that while molality must be employed to generate the PMV curves, the data are converted to units of molarity in Figure 2 to align with the constant-volume basis of density measurements. The thermodynamic PMV employed here differs fundamentally from the apparent molar volume commonly reported in the literature,^72^ especially at high salt concentrations (see Sections S1.2.A and S1.4 in the SI for additional discussion on this point).

Changes in $V_{m}^{LiCl}$ and $V_{m}^{H_{2}O}$ as a function of the LiCl concentration are provided in Figure 2b and 2c, respectively. The black points and curves are from experimental measurements. Significantly, $V_{m}^{LiCl}$ rose continuously from low concentration to ∼6.7 M, where it reached a maximum. Above this value, it began to decrease. $V_{m}^{H_{2}O}$ showed the opposite trend with a minimum at ∼6.7 M. Such reciprocal behavior is a direct consequence of the Gibbs–Duhem relation,^73^ which thermodynamically couples the PMV values for multicomponent systems. The pronounced nonlinearity observed in both PMV curves underscores the significance of higher-order interactions in concentrated solutions. Indeed, fitting the PMV data required terms up to fourth order from eq 1 for both the salt and water to accurately model the data over the entire concentration range (see Section S1.2.B in the SI for details).

A microscopic interpretation of the PMV trends requires theoretical insight from MD simulations employing force fields capable of reproducing the trends. In density-driven parametrization, it is standard practice to refine the Lennard-Jones (LJ) parameters, ϵ and σ, associated with the components (here Li^+^ and Cl^–^) to match experimental data. This could be done equally well for the density and the PMV by using interaction potentials having either scaled^28^ or unscaled charges^42^ (see Section S3.4 in the SI). In the ensuing analysis, unscaled charges were selected. The resulting trends for solution density and the PMV with the optimized FF (**JC**_opt_) are shown as red data points and curves in Figure 2. The level of agreement between the experiment and simulation is striking and cannot be achieved with off-the-shelf force-fields (see Section S3.4.A in the SI). Moreover, **JC**_opt_ semiquantitatively captured both the maximum in the PMV for salt as well as the minimum with water at approximately 7 M, which was only ∼4.5% higher than the experimental values. As described below, these features arose from structural changes involving correlated ions, such as those produced by ion-pairing interactions.

### 3.2 Evolution of Ion Networks

One of the most widely used approaches for characterizing liquid structure is the analysis of the local coordination environment. To this end, 1 μs NPT simulations were employed to determine the radial distribution functions (RDFs: g­(r)) between different species, as well as their corresponding radial integrals—the coordination numbers (CNs).^74^ For each ion–ion and ion–water pair, CN was evaluated by integrating g­(r) up to the first minimum, thereby including the first hydration shell of the ions.

Figure 3a and 3b illustrate the CNs for Li–O and Li–Cl, respectively, which follow the changes in local structure around the cation with increasing salt concentration. As expected, the CN_Li–O_ falls, while the corresponding value for Li–Cl rises as water is displaced around Li^+^ by Cl^–^ at higher salt concentration. The structural evolution in CN is illustrated by snapshots that show how a hydrated Li^+^ coordinated by 4 water molecules evolves into structures consisting of contact ion pairs and solvent-shared ion pairs^3^ (CIP and SIP). Significantly, CN_Li–O_ and CN_Li–Cl_ both present rapid changes beyond 5 M after which the maximum/minimum in the PMVs are also observed (Figure 2b and 2c, ∼6.7 M). For CN_Li–Cl_, one begins to see pairs of CIPs sharing water molecules at higher LiCl concentrations (upper-right schematic in Figure 3b). Structures and networks can be more precisely quantified by examining the CNs associated with the Li–Li and Cl–Cl g­(r)­s (see Figures S3.5 and S3.6 in Section S3.4.B in the Supporting Information). Beyond the inflection point at ∼7 M, a strong tendency is observed for like-ions to come into close proximity.

**Figure 3:**
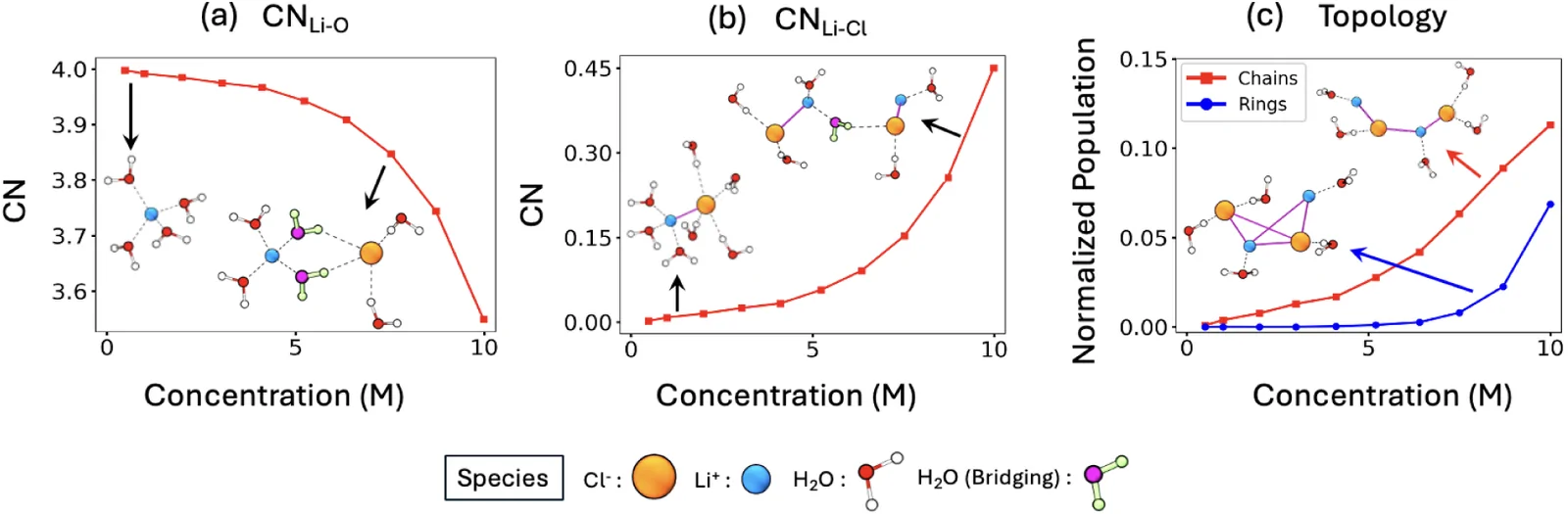
Ion network reorganization with concentration: (a) Li–O first-shell CN, showing the evolution from isolated, fully solvated ions to ion-pair configurations; (b) Li–Cl first-shell CN, highlighting reduced separations between ion pairs facilitating higher-order aggregate formation; (c) Ring and chain populations in the ion network with representative examples in the insets. The populations are normalized against the number of ions at the corresponding concentration. The cutoff distances for defining these structures are provided in Table S3.4.

A more quantitative framework for analyzing structural changes requires moving beyond simple pairwise correlations. A useful strategy is to map atomic configurations into a network of connected nodes and then apply graph-theoretical analysis.^75–78^ To this end, we built chemical graphs, treating ions (Li^+^ and Cl^–^) as nodes and introducing edges between them based on their physical proximity, determined from the RDFs (see Section S3.5.A in the SI). The advantage of a graph-based representation is that it allows nonlocal connectivity patterns to be identified. In contrast to RDFs or CNs, graphs inherently capture nonlocal associations beyond simple pairwise interactions, making it possible to directly visualize the emergence of extended clusters and networks within a defined cutoff distance. Once connectivity is defined, the configuration is naturally partitioned into ion clusters and aggregates of various sizes. For instance, an ion pair consisting of Li^+^ and Cl^–^ in direct contact corresponds to a cluster of size *i* = 2, while larger aggregates reflect an increasing extent of ion aggregation, forming motifs that involve multiple interconnected ion pairs (see Section S3.5 in the SI for further discussion).

Two of the most common topological structures examined in graph-theory are the formation of closed rings^79,80^ (primitive) and open connected chains^81,82^ (paths/walks). The propensity to form these structures as a function of concentration is plotted in Figure 3c. Chain and ring motifs are rare below 6 M; however, above this threshold their concentrations rise sharply. The schematics in Figure 3c illustrate a ring made from two trimer-like structures and a chain, consisting of two ion pairs. In both cases, the creation of these topological motifs is facilitated by attractive forces between oppositely charged ions that offset local electrostatic repulsions between like-charged ions.

In addition to elucidating the topology of the evolving ion networks, the systematic classification of ions into clusters/aggregates of different sizes provides a rigorous microscopic basis for interpreting experimentally measured PMVs, thereby linking molecular-level structure and organization with macroscopic thermodynamic properties. This idea is explored in detail in the next section.

### 3.3 Microscopic Origins of the PMV

The PMV virial expansion shown in eq 1 is typically interpreted as a perturbative correction to an ideal reference system. Figure 4a decomposes the experimental salt PMV (black data points and curve) into separate contributions from an initial value at infinite dilution (B_0_) plus the linear (B_1_), quadratic (B_2_), cubic (B_3_) and quartic (B_4_) virial terms. In contrast to the case for density, where the intercept term of the virial expansion would represent the density of pure water (independent of ion population), the B_0_ term for the PMV represents a first-order (one-body) ion contribution. In other words, it reflects the volume change associated with introducing an infinitesimal amount of LiCl into pure water—capturing both the intrinsic van der Waals volume of the ions and the net electrostriction of the surrounding water molecules. In fact, B_0_ is directly related to the ratio of the zero-order, A_0_, and first-order, A_1_, virial coefficients for the density (see Section S1.2.C in the SI for details):

3 $$B_{0}=\frac{1}{A_{0}}\left\{M_{LiCl}-1000\left(\frac{A_{1}}{A_{0}}\right)\right\}$$

**Figure 4:**
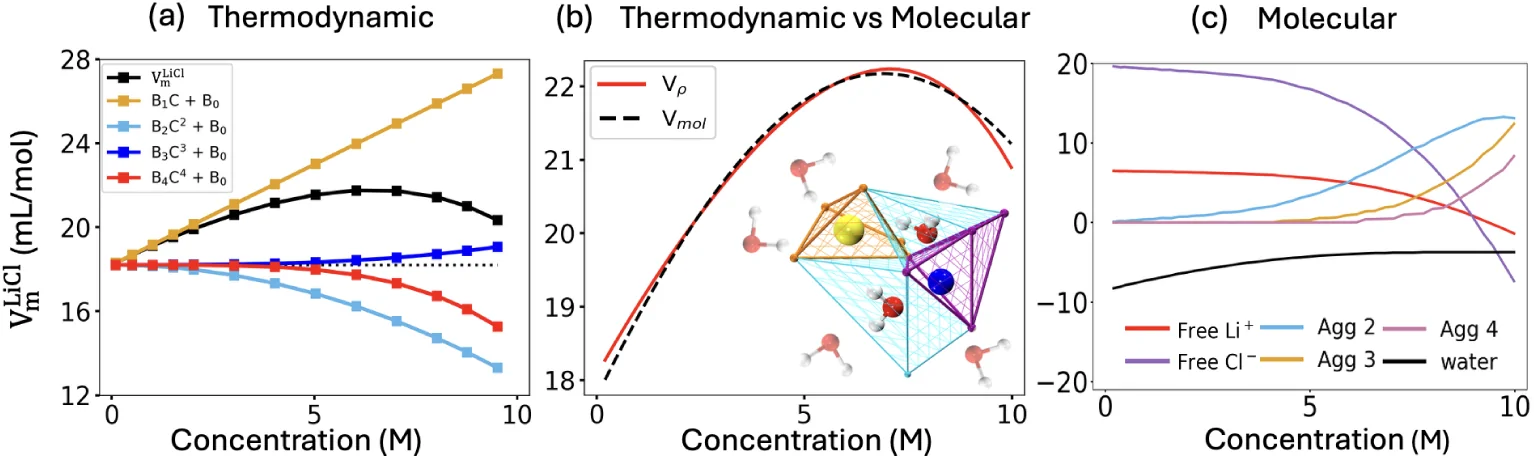
(a) Evolution of the thermodynamic virial expansion components for $V_{m}^{LiCl}$ with concentration starting from B_0_; (b) Comparison between $V_{m}^{LiCl}$ curves from density derivatives and the Laguerre polyhedral volumes (inset: example of said polyhedron); (c) $V_{m}^{LiCl}$ contributions from aggregates of different sizes (solvated ions up to a size of 4): ion cluster trends from the bracketed terms in eq 5, water from the final term.

The higher-order coefficients (B_1_–B_4_) have alternating signs as can be seen in Table 1 and Figure 4a. This reflects a competition between expansion and contraction effects as the concentration of ions increases. Specifically, B_1_ (dark yellow points and line, a 2-body effect) has a positive sign representing an increase in the measured ionic volume. The expansion arises from a competition for individual water molecules between two ions as depicted by the multicolored water spheres (dark blue/navy blue spheres) in Figure 1c. In a virial expansion, this term increases the volume continuously with concentration, so that a negative quadratic term (Figure 4a, B_2_, light blue points and curve reflecting 3-body effects) is required to offset $V_{m}^{LiCl}$ back to the measured result (black data points and curve). As can be seen, B_2_ becomes significant near 2.5 M. Next, the positive B_3_ and negative B_4_ contributions (dark blue and red points and curves in Figure 4a) are much less pronounced before ∼6.7 M. These trends underscore the fact that higher-order contributions (beyond 3-body effects) only become significant at substantial salt concentrations. Curiously, although the value of B_3_ is larger than B_4_ (Table 1), the PMV contribution for the latter dominates due to the difference in cubic and quartic concentration dependence.

**Table 1:** Average Fit Coefficients

Coefficient	B_0_	B_1_	B_2_	B_3_	B_4_
Value	18.3815	0.9077	–0.0506	0.0009	–0.0003

These coefficients cannot directly elucidate the microscopic origins of the PMV. Specifically, they fail to succinctly capture how molecular effects such as ion-pairing and the evolution of the ion–water network manifest themselves at different length scales. Schematics like those shown in Figure 1 depicting nonlocal effects involving both ion and solvent reorganization do not provide a quantitative probe for how the PMV evolves at the molecular level. Furthermore, changes in the volume of water as a function of concentration are only implicitly accounted for through the B_*n*_ coefficients in the virial expansion.

To develop a molecular level model for the behavior of the PMV as a function of salt concentration, it is necessary to quantify the molecular volumes of individual water molecules and ions in solution. This can be done by analyzing configurations produced by MD simulations with spatial decomposition methods, such as Voronoi tessellation. This technique seamlessly partitions space into nonoverlapping polyhedral cells associated with individual particles and has been widely used to define local geometric volumes in molecular systems.^37,83–87^ Here, we employed a weighted variant, radical Voronoi (or Laguerre) tessellation,^36,37,85^ to account for atomic radii as weights. This procedure partitions the total volume into individual subregions which represent local volumes for ions and water molecules, assigning larger volumes to species with larger radii and vice versa (see Section S3.6 in the SI).

Using Laguerre tessellation, it is possible to reconstruct the PMV from isolated ions, water molecules, and ion clusters that emerged from the construction of the chemical graphs introduced in the last section. Dissecting the system into contributions arising from ion clusters of different sizes provides a chemically intuitive means to rationalize the PMV in terms of multiple ion-pairs. In contrast, the definitions of clusters in the virial expansion^32^ incorporate both direct and indirect correlations and are therefore not limited to groups of particles within a geometric contact distance, which make them challenging to interpret.

The total volume can be written as a sum of the microscopic volume contributions of the individual components in the mixture: ions (Li^+^ and Cl^–^) plus water molecules. As such, it can be shown (see Section S3.6.A in the SI) that the PMV for salt is equivalently given by the following expression (eq 5):

4 $$V_{m}^{LiCl}=\underset{\mathrm{Ion Contribution}}{\underset{︸}{\underset{i\in \left[Agg\right]}{\sum }\left[f_{i}\left(m\right){\langle V_{\mathrm{mol}}\left(m\right)\rangle }_{i}+m\frac{\partial }{\partial m}\left\{f_{i}\left(m\right){\langle V_{\mathrm{mol}}\left(m\right)\rangle }_{i}\right\}\right]}}+\underset{\mathrm{Water Contribution}}{\underset{︸}{\frac{1}{M_{H_{2}O}}\frac{\partial {\langle V_{\mathrm{mol}}\left(m\right)\rangle }_{H_{2}O}}{\partial m}}}$$

where *m* is the solution molality, $M_{H_{2}O}$ is the molar mass of water (in kg), *f*_*i*_ denotes the population fraction of species *i*, normalized by the total count of LiCl formula units, and ⟨*V*_mol_⟩_*i*_ represents the corresponding average microscopic volumes. The index *i* spans all aggregate types present at a given concentration starting with isolated (solvated) Li^+^ and Cl^–^ that dominate at low concentration. Ion contributions to the PMV, partitioned into different cluster sizes, are controlled by the product of *f*_*i*_ and ⟨*V*_mol_⟩_*i*_. While *f*_*i*_ values for single ions drop as a function of concentration, the contributions from dimers, trimers, and tetramers all grow appreciably beyond 5 M as shown in Figure S3.11 in the SI.

The microscopic volume terms, ⟨*V*_mol_⟩_*i*_, vary only modestly as a function of salt concentration (see Figure S3.13 in the SI), while changes in *f*_*i*_ are often more substantial. Equipped with both *f*_*i*_ and ⟨*V*_mol_⟩_*i*_, we can construct the PMV. Figure 4b compares the PMV trends obtained from simulations of the macroscopic density (solid red line), with those derived from the Voronoi-like molecular volumes (black dashed line). The PMV built from clusters ranging from size 1 (isolated ions) to 4 (rings and chains) closely reproduces the thermodynamic PMV. Although models restricted to smaller aggregates qualitatively capture the concentration dependence of the PMV, inclusion of clusters up to size 4 yields a more physically consistent and quantitatively accurate description of the thermodynamic PMV (Section S3.6.C in the SI).

Having established a microscopic model of molecular volumes that quantitatively match the thermodynamic PMV, we can now analyze how clusters of varying sizes contribute to the total volume as a function of concentration. Figure 4c breaks down the PMV by cluster size (1–4) as well as the total contribution arising from the water. Strikingly, the net PMV involves compensating contributions arising from the various-sized clusters, all of which change their volume contribution as a function of salt concentration. For example, the hydrated free Cl^–^ contribution decreases markedly from 19.6 mL/mol at infinite dilution to 13.0 mL/mol at 6.7 M, while Li^+^ only shows a modest reduction from 6.5 to 4.4 mL/mol (Figure 4c, purple and red curves). The two-body contribution, which corresponds to single ion-pairs, grows continuously above 0.1 M, increasing to a value of ∼7.0 mL/mol close to 6.7 M (Figure 4c, blue curve). Thus, the decrease in hydrated ion contributions is almost exactly offset by the increase of the two-body contribution below 6.7 M. These features are also fully consistent with the structural adjustments associated with ion solvation and ion-pairing (see Figure 3a,b).

For the water contribution in eq 5, all water molecules in the simulation box are treated as a single collective entity to circumvent combinatorial complexity. The simulation results show that the ∼4.4 mL/mol increase in $V_{m}^{LiCl}$ from 0 to 6.7 M (Figure 4b) originates from competitive electrostriction of water molecules (Figure 4c, black curve). Indeed, electrostriction weakens with increasing salt concentration, and its attenuation is primarily responsible for the rise in $V_{m}^{LiCl}$ up to the maximum. Beyond 6.7 M, electrostriction saturates, and the water contribution remains constant. In the high salt concentration regime, water molecules are shared among multiple ions, and further addition of salt promotes ion pairing rather than solvation.

Above the maximum, $V_{m}^{LiCl}$ decreases by approximately 1.4 mL/mol between 6.7 and 10.0 M (Figure 4b). Over this concentration range, the contributions of individual Cl^–^ and Li^+^ ions decrease substantially, by 20.4 and 5.8 mL/mol, respectively. As noted above, this behavior is driven by the diminishing population of isolated, fully hydrated ions. By contrast, the emergence of higher-order correlations is reflected in the growth of 3- and 4-body cluster contributions beyond 6.7 M, which increase by 10.6 and 8.0 mL/mol, respectively. These trends indicate that, at elevated concentrations, the electrolyte undergoes a pronounced structural reorganization involving clusters of all sizes.

The Voronoi tessellation approach described above underscores the fact that a molecular-scale decomposition is not accessible from the magnitudes, signs, or even cumulative contributions of the virial coefficients (B_1_–B_4_) alone. This represents a serious limitation to interpreting the virial expansion without explicit structural resolution.

### 3.4 Probing Hydrogen Bond Network through Raman Spectra

The preceding analyses reveal collective synergistic responses of cations and anions across the full concentration range. The substantial water contribution up to the $V_{m}^{LiCl}$ maximum at 6.7 M reflects electrostriction, but this contribution by itself does not directly quantify how the underlying hydrogen-bonding network evolves. Radial distribution functions from our simulations (Figure S3.8 in the SI) point to a distortion of the hydrogen-bonding structure with reduced orientational ordering between pairs of neighboring waters, partially compensated by the donation of hydrogen bonds to Cl^–^ ions. These structural signatures suggest a reorganization of the hydrogen bonding network, consistent with past studies,^5,88,89^ that cannot easily be resolved from volumetric observables alone, motivating a direct spectroscopic probe of the water structure.

Raman spectroscopy measurements were performed to explore the vibrational spectra of water in the OH stretch region (3000–3800 cm^–1^). Specifically, changes in the frequency and intensity of OH stretch resonances reflect the restructuring of the hydrogen bonding network as the salt concentration is increased. As a general rule, interactions that strengthen hydrogen bonds cause the peak to red shift in frequency, whereas weak or broken hydrogen bonds result in blue shifts, closer to the gas-phase (vacuum) frequency.^90–93^

To isolate and quantify changes, Raman spectra were deconvoluted using the multivariate curve resolution (MCR) method.^39,40^ For clarity, the extracted solute-correlated spectral signatures for just 0.5, 4.0, and 8.0 M solutions are presented in Figure 5a, while additional spectra over the entire concentration range are provided in the SI (Section S2.1.B). The OH stretch profiles obtained from MCR exhibit a nearly monotonic increase in the solute-correlated component across the full range. This trend directly reflects the growing population of perturbed water molecules in the hydration shell of the ions compared with unperturbed bulk water. In addition to the enhancement of the dominant central peak at ∼3440 cm^–1^, spectral deconvolution of the solute-correlated signal into Gaussian components reveals two distinct shoulder features: one red-shifted to ∼3270 cm^–1^ and the other blue-shifted to ∼3590 cm^–1^.

**Figure 5:**
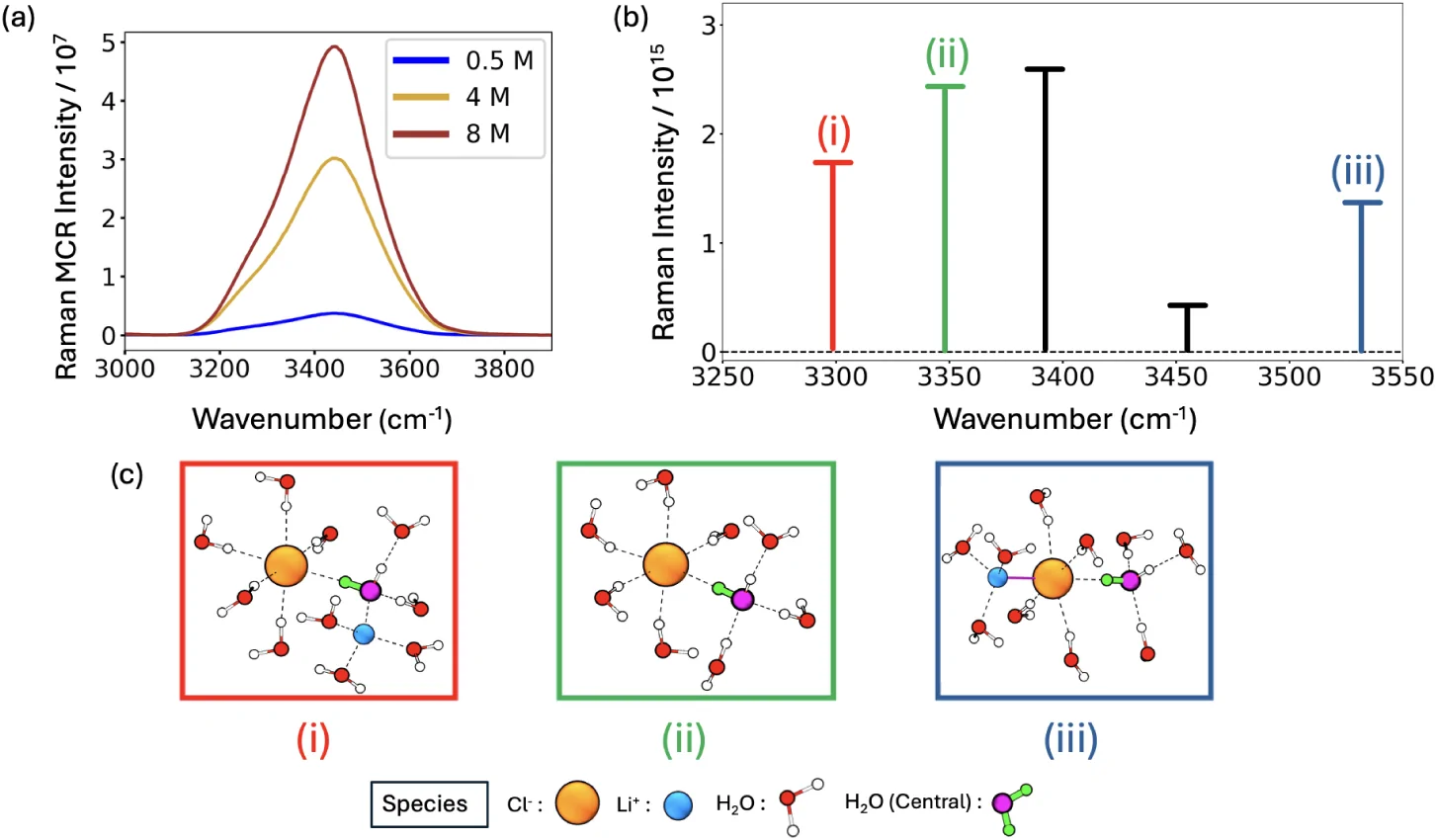
(a) Solute-correlated Raman data obtained by MCR at 0.5, 4.0, and 8.0 M. Note: additional isotopic dilution experiments were performed using a 1:9 H_2_O/D_2_O mixture. After decoupling the OH oscillators, the OH stretch region still gave rise to three distinct peaks upon performing MCR. These experiments confirmed that both the low and high frequency shoulders do not merely arise from intramolecular coupling of the OH stretch modes (see Section S2 in SI). (b) A Raman spectrum from harmonic normal mode calculations. The two black vertical lines denote the OH stretch frequencies of pure water and are provided for reference. The vertical lines at 3294 cm^–1^ (red), 3347 cm^–1^ (green), and 3532 cm^–1^ (blue) correspond to (c) illustrations of (i) a solvent shared ion pair, (ii) a solvated chloride ion and (iii) a contact ion pair, respectively.

In order to elucidate the physical origins of the spectral features from the MCR Raman data, we performed quantum-chemistry calculations on model ion–water clusters inspired by our preceding analysis. The abstracted Raman resonances are provided in Figure 5b, and the corresponding structures are shown in Figure 5c­(i)-(iii). Details on how these clusters were constructed can be found in Section S3.7.B of the SI. In the absence of explicit dynamical effects and the full ion environment, these calculations were intended to capture trends rather than absolute values. We focused on three distinct structural motifs inspired by our ion clusters (color coded in red, green, and blue), which helped rationalize the concentration dependence of the spectra and the putative emergence of the three experimental components observed from their deconvolution. The water molecules that give rise to these spectral features are enlarged and their oxygens are colored violet in Figure 5. The specific OH bonds involved in the corresponding vibrational modes are shown in green.

The black lines shown in Figure 5b at 3383 and 3453 cm^–1^ correspond to the symmetric and asymmetric OH stretch modes of bulk water, respectively. When salt was introduced, a series of resonances with frequencies ranging from 3330 to 3470 cm^–1^ arose which corresponded to water molecules hydrating Cl^–^ ions (represented by the vertical green line in Figure 5b). We therefore assigned the growth of the experimental peak at 3440 cm^–1^ to the first hydration shell around the anions (see the illustration in Figure 5c­(ii)) with the OH bond pointing toward the Cl^–^ anion. This produces a calculated vibrational mode at 3347 cm^–1^, consistent with previous studies.^89^

As the electrolyte concentration increased, ion pairing became a dominant structural feature, as established above through the PMV study. Some of the simplest consequential motifs: water molecules sandwiched between Li^+^ and Cl^–^ ion pairs (solvent shared ion pairs, see Figure 5c­(i)) generally resulted in the most red-shifted calculated OH resonances with frequencies ranging between 3100 and 3300 cm^–1^ (red vertical line in Figure 5b). This pronounced red shift can be understood in terms of a cooperative interaction between the cation and anion with Li^+^ acting as a Lewis acid, enhancing the hydrogen-bond–donating ability of the sandwiched water molecule, while Cl^–^ gave rise to a strong hydrogen-bond–accepting interaction. The combined effect substantially weakened the covalent OH stretch bond, leading to the experimentally observed red shift at 3270 cm^–1^.

Finally, a Raman active mode close to the experimental peak at 3590 cm^–1^ led to the identification of a cluster where water molecules solvated a contact ion pair (see Figure 5c­(iii)). More specifically, it was found that water molecules solvating Cl^–^ involved in contact ion pairing with Li^+^ contributed to a calculated vibrational signature near 3532 cm^–1^ (Figure 5b). The presence of Li^+^ in direct contact with Cl^–^ effectively reduced its ability to accept a hydrogen bond from surrounding water molecules.

## 4 Discussion

### 4.1 Electrolyte Models

Several theoretical frameworks have been formulated to interpret the virial expansions typically observed in thermodynamic measurements of electrolyte properties. For example, McMillan-Mayer (MM) theory is a statistical mechanical model that can be applied to liquids with dissolved solutes.^32,33^ MM integrates out the solvent and treats the remaining solutes as if they were gas molecules confined inside a container. In this model, ion interactions are described by a potential of mean force (PMF) that averages over all possible solvent configurations. This approach is only valid at low salt concentrations. As such, it is difficult to employ MM to glean molecular-level insights into ion structures and clustering. Instead, many investigators employ Kirkwood–Buff (KB) theory^94–98^ and the associated Kirkwood–Buff integrals (KBI) to relate pair correlation functions to thermodynamic properties at a given concentration. KB theory provides a powerful and general framework for connecting two-body correlations and solution thermodynamics. However, the thermodynamic quantities it yields are expressed in terms of integrals over radial distribution functions and do not, by construction, distinguish between specific real-space motifs such as isolated ions, ion pairs, chains, and rings. In the present work, we therefore pursue a complementary, explicitly real-space approach in which the PMV is decomposed into contributions from identifiable structural motifs and local volumes.

In addition to rigorous theories, empirical models have been developed to predict the behavior of electrolyte solutions. This includes work by Pitzer^99,100^ who employed a Debye–Hückel term to describe electrostatic interactions and screening at low salt concentrations in addition to a virial expansion. Curiously, the Pitzer model sets the linear term (B_1_ in our notation) equal to zero and therefore describes pairwise interactions between ions strictly with the B_2_ coefficient. Unfortunately, eliminating the linear term discards the key role of competitive water electrostriction in changing $V_{m}^{salt}$ . Another more recent empirical model is the three-characteristic parameter correlation^101,102^ (TCPC). However, the performance of TCPC has typically been found to be poorer than Pitzer theory.^103^ Also, Schwaab and Pezzotti have recently developed a theory of electrolyte solutions where the volume-environment interactions are incorporated through a generalized multipole expansion.^104^

By contrast with previous theories and methods, the present framework treats the PMV as a molecular observable that directly reflects the collective structural organization of ions in electrolyte solutions. As described above, this method works by experimentally obtaining $V_{m}^{salt}$ over a wide range of salt concentrations. These values are then used to fine-tune force fields for all-atom MD simulations. Even by employing this procedure, the power series contributions to $V_{m}^{salt}$ (Figure 4a) are still challenging to interpret because ion–ion interactions involve a mixture of local and nonlocal contributions. For example, it is difficult to elucidate the relative contributions of solvent separated, solvent shared, and contact ion interactions in the B_*n*_ terms. This obstacle to abstracting chemically useful information can be circumvented by using conformations generated with MD simulations and defining isolated ion, two-body, three-body, and four-body interactions strictly in terms of direct contact interactions. Figure 4c does precisely that. From this construction, it is straightforward to visualize the evolution of ion clustering as a function of salt concentration.

Defining ion clusters through contact interactions is not a unique choice. In fact, there should be numerous useful definitions that can be employed to understand electrolyte behavior. For example, one could place solvent shared, solvent separated and contact pairing interactions into separate categories.^105,106^ This might even be a judicious means of unraveling the relative contributions from each category that lead to the B_*n*_ terms shown in Figure 4a. In this regard, the use of unsupervised learning approaches leveraging local-atomic descriptors^107–111^ to identify complex patterns of ions and water molecules would be an interesting avenue to explore in the future.

### 4.2 The Evolution of Ion Clustering

Figure 4c is a useful starting point for elucidating the molecular-level details of many-body interactions. For example, clusters of size 3 (Agg 3) consist of two different species: ${\mathrm{LiCl}}_{2}^{-}$ and Li_2_Cl^+^ (Figure 6a, top). These aggregates not only have opposite charges, but our MD simulations reveal that the anionic cluster is more abundant than the cationic one (see Figure S3.9 in the SI). This is what ultimately leads to the faster decrease in free Cl^–^ ions compared to free Li^+^ ions at higher salt concentrations (Figure 4c). The origin of this asymmetry is likely rooted in the fact that Cl^–^ accepts more oriented hydrogen bonds compared to Li^+^ leading to more charge-dipole interactions for the anionic cluster. By contrast, clusters of size 4 are predominantly neutral (Li_2_Cl_2_, Figure 6a, bottom), while the other four possible combinations (Li_3_Cl^2+^, ${\mathrm{LiCl}}_{3}^{2-}$ , ${\mathrm{Li}}_{4}^{4+}$ , or ${\mathrm{Cl}}_{4}^{4-}$ ) are found with very small probability (Figure S3.9 in the SI). Both Agg 3 and Agg 4 clusters become increasingly important at higher concentrations (Figure 6b).

**Figure 6:**
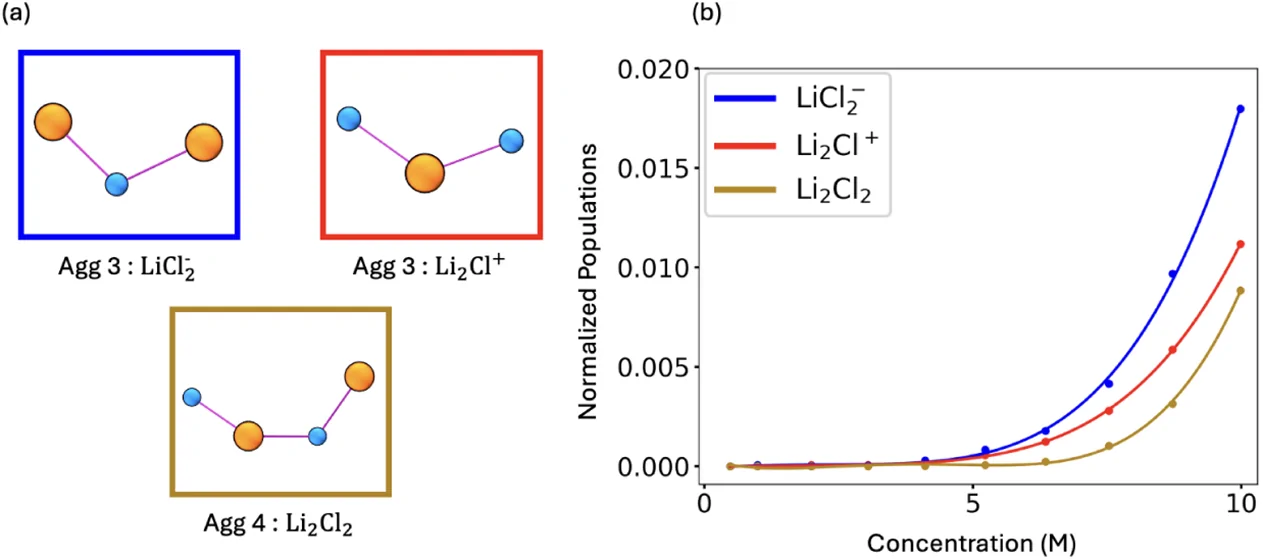
(a) Example of clusters of formula unit: ${\mathrm{LiCl}}_{2}^{-}$ , Li_2_Cl^+^, and Li_2_Cl_2_. (b) The evolution of the time averaged populations of the corresponding species with concentration. Populations are normalized by the total number of ions at the respective concentrations.

As can be seen, the populations of the cationic and anionic Agg 3 cluster are both individually larger than the combined populations of all Agg 4 clusters (chains + rings). This is significant as the thermodynamic data in Figure 4a clearly show that B_4_[LiCl]^4^ becomes more significant than B_3_[LiCl]^3^ at higher salt concentrations. As such, looking at the population of Agg 4 made strictly from contact ion interactions underweights 4-body interactions while simultaneously overweighting 3-body interactions. This interpretation is consistent with the recent work from Dinpajooh and coworkers^112^ in which molecular dynamics-derived short-range ion interactions provide the microscopic basis for the emergence of larger-scale charge correlations in concentrated electrolytes.

As noted in the introduction, accurate $V_{m}^{salt}$ and $V_{m}^{H_{2}O}$ curves were not available before the current study. The reason for this is 2-fold. First, many previous volume^113^ and density measurements^114–116^ of electrolyte solutions did not have sufficient precision and accuracy to produce reliable PMV curves. Second, one needs to employ density data in combination with eqs 2 and 3 rather than using procedures that yield the apparent molar volume^116–123^ (see Section S1 in the SI for further discussion).

With proper curves in hand, it is possible to compare the data in Figure 2b and 2c to a wide variety of thermodynamic properties for LiCl solutions. Remarkably, the shape of the $V_{m}^{H_{2}O}$ curve is reminiscent of data for the freezing point depression of LiCl solutions as a function of salt concentration (see Figure S1.6 in the SI). In particular, the eutectic point^125^ occurs at 6.8 M, which is quite close to the minimum in $V_{m}^{H_{2}O}$ near 6.7 M, despite the fact that the eutectic point is roughly 100 °C below the temperature at which density measurements are made in the current study (20 °C). This fact suggests that the organization of ion clusters is only weakly dependent on temperature. Moreover, the eutectic point and PMV minimum/maximum occur almost exactly at the same salt concentration where the chemical potential of water falls most rapidly as determined by vapor pressure osmotic (VPO) studies^126^ (see Figure S1.6 in the SI).

The interconnected nature of PMV, VPO, and eutectic point measurements suggests that 6.7 M LiCl represents a pivotal salt concentration. In fact, this is also near the concentration where the second derivative of the cluster size 2 (Agg 2) curve reaches a maximum (see Figure S3.12 in the SI). In addition, clusters of size three and four (Agg 3 and Agg 4) become significantly more prominent above this point. Moreover, the volume contraction of water molecules shrinks until 6.7 M but has a constant contribution to $V_{m}^{LiCl}$ at higher salt concentrations (Figure 4c). Taken together, it appears that the macroscopic thermodynamic properties of the solution are closely linked to microscopic ion pairing behavior at this concentration.

Next, the three Gaussian peaks resolved in the Raman MCR spectra (see Figure 5 and Figure S2.1 in the SI) confirm the idea that water behaves differently beyond 6.7 M LiCl. Figure 7 presents the concentration dependence of the areas for these components, where panels (a), (b), and (c) correspond to the 3270, 3440, and 3590 cm^–1^ peaks, respectively. In particular, the area under the 3440 cm^–1^ peak increases continuously, but it goes up more gradually at higher concentration, with only about 7% of the increase occurring above 6.7 M (Figure 7b). This is direct spectroscopic evidence that complete first hydration shells form around isolated Cl^–^ species at lower concentrations (Figure 5c­(ii)). However, sufficient water is no longer available beyond this point to accommodate complete hydration of additional salt ions.

**Figure 7:**
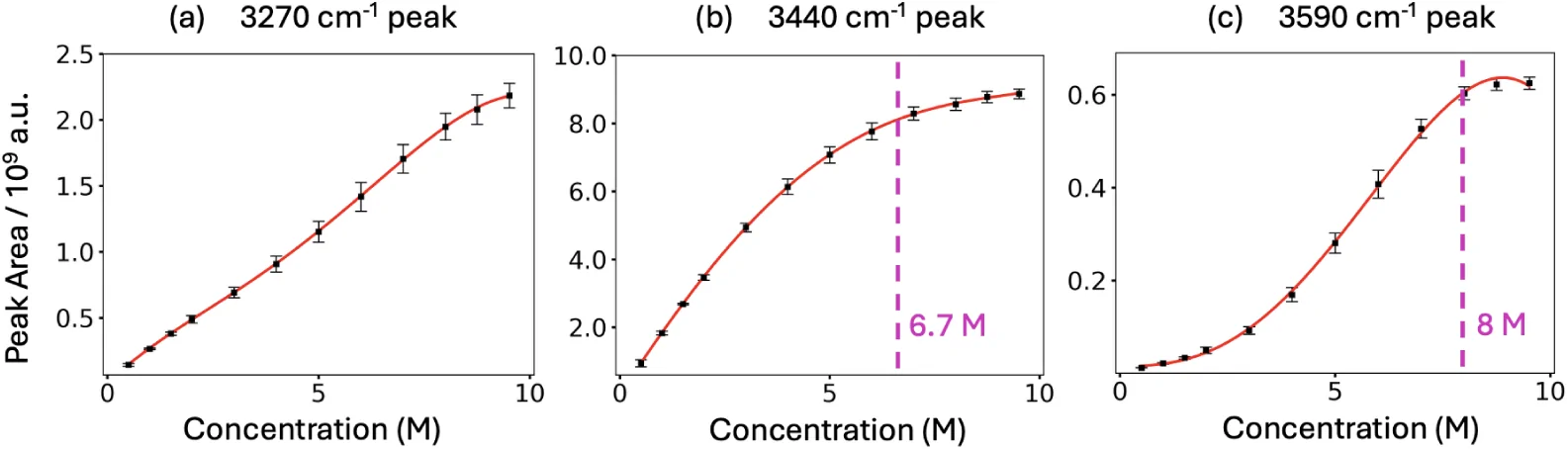
Evolution of the area under the Gaussian components resolved from the solute-correlated part of the Raman signal centered at (a) 3270 cm^–1^, (b) 3440 cm^–1^, and (c) 3590 cm^–1^. The data points are drawn with error bars that come from the standard deviation from three independent trials.

The 3590 cm^–1^ resonance follows a similar trend to the 3440 cm^–1^ peak but does not begin to level off until a somewhat higher salt concentration (>8 M LiCl, Figure 7c). This higher frequency peak represents OH groups that point toward Cl^–^ ions that are in contact pair configurations with Li^+^ on the opposite side of the anion (Figure 5c­(iii)). As such, the electron density around Cl^–^ is distorted by Li^+^, which attenuates the charge density that can be transferred from the anion into the σ* orbital of water molecules that are hydrogen bonded to it.^127^ Moreover, the increase in the 3590 cm^–1^ peak area roughly follows the shape of the Agg 2 cluster curve (Figure 4c), which also levels off near 8 M LiCl. By contrast, the 3270 cm^–1^ resonance continuously grows from low LiCl concentration to 9.5 M (Figure 7a). This is consistent with the fact that the concentration of solvent shared ion pairs continuously increases throughout the entire concentration range (Figure 5c­(i)). At relatively low salt concentrations, water molecules that interact with both a cation and an anion largely represent solvent-shared ion pairs without other ions in the vicinity. At higher salt concentrations, these same water molecules are increasingly shared between adjacent chains and rings.

### 4.3 Toward Reliable Electrolyte Models

The refinement of classical empirical potentials for modeling electrolyte solutions is an active area of current research. Specifically, the scaling of charges on ions is considered to be an important means for modeling electronic screening effects. We show that LJ parameters can be optimized for both scaled and unscaled charges to reproduce experimental PMV values. This provides an option to compensate for very different partial charges consistent with recent reports by Fan and coworkers.^128^ However, simultaneously reproducing surface tension, viscosity, VPO, conductivity, and PMV measurements across diverse salts and concentrations may require the use of a fixed charge scaling value. Moreover, as more precise electrolyte work is done, relaxation of the Lennard-Jones combination rules and/or the incorporation of additional functional forms, such as empirical charge–dipole interactions, may be required. However, it is becoming clearer that highly accurate experimental measurements are a necessary prerequisite for generating reliable ion force fields for use in simulations. Specifically, the use of 20th century data for parametrizing ion force fields in water should be done with caution. Only properly measured and curated data sets^129–131^ should be considered (see Section S1.4.B in the SI for additional discussion on this point).

Beyond the optimization of force fields, renewed attention to the generation of improved electrolyte models should have broad ramifications for understanding ion-specific phenomena across chemistry, biology, and materials science. Indeed, the Hofmeister series has been known for 138 years, yet the underlying molecular-level reasons for the recurring ranking of ions across a wide variety of physical phenomena still remain the subject of intense debate. We suggest that the lack of high-quality electrolyte data represents a crucial reason why Hofmeister chemistry has not yet been better understood. In fact, deeper insight will require the continued integration of thermodynamics, spectroscopy, and molecular simulation for electrolyte studies, as exemplified by the present work.

## Supplementary Material





## Notes

The experimental density and PMV data (CSV)
Section S1: Experimental density and partial molar volume; Section S2: Raman MCR measurements; and Section S3: Computational methods (PDF)
