# Supplementary material for: Beyond the Virial Expansion: Microscopic Origins of Partial Molar Volumes in LiCl Solutions
Source: J Am Chem Soc. 2026 Sep 24;148(39):41978–90. doi: 10.1021/jacs.6c08519 (PMC13651509; doi:10.1021/jacs.6c08519)
Supplement: Supplementary file 1 [file ja6c08519_si_002.pdf]

Supporting Information for

Beyond the Virial Expansion: Microscopic Origins of Partial Molar Volumes in LiCl Solutions

Chun-Ting Lin,[†,⊥] Diganta Dasgupta,[‡,¶,⊥] Tinglu Yang,[†] Cesare Malosso,[§] Giulia Sormani,[¶] Colin Egan,[‖] Giovanni Bussi,[¶] Ali Hassanali,[*,‡] Paul S. Cremer[*,†]

[†] Department of Chemistry, The Pennsylvania State University, University Park, Pennsylvania 16802, United States of America

[‡] Condensed Matter and Statistical Physics, The Abdus Salam International Centre for Theoretical Physics (ICTP), Trieste 34151, Italy

[¶] Scuola Internazionale Superiore di Studi Avanzati (SISSA), Trieste 34136, Italy

[§] Laboratory of Computational Science and Modelling, IMX, École Polytechnique Fédérale de Lausanne, Lausanne CH-1015, Switzerland

[‖] Initiative for Computational Catalysis, Flatiron Institute, New York 10010, United States of America

[⊥] These authors contributed equally

[*] Email: ahassana@ictp.it; psc11@psu.edu
Phone: +39 040 2240 4175; (814) 865-6259

Contents

S1 Experimental Density and Partial Molar Volume **S3**
S1.1 Density Measurement and Analysis for LiCl solution . . . S3
S1.2 Derivation and analysis of Partial Molar Volumes . . . S4
S1.2.A Derivation of PMV . . . S4
S1.2.B PMV from Measured Densities and Error Analysis . . . S7
S1.2.C Connecting the Density and PMV Coefficients . . . S8
S1.3 Experimental Datasets . . . S9
S1.4 Comparison of the PMV and Density with Available Literature . . . S11
S1.4.A Partial Molar Volume . . . S11
S1.4.B Density . . . S12
S1.5 PMV of Water, Eutectic Point and Vapor Pressure of LiCl Solutions . . . S13

S2 Raman MCR Measurements **S15**
S2.1 Raman Spectra of LiCl solutions and MCR analysis . . . S15
S2.1.A Instrumentation . . . S15
S2.1.B Theory and Spectral Analysis . . . S15

S3 Computational Methods **S19**
S3.1 Force-Field Optimization . . . S19
S3.1.A Background . . . S19

S3.1.B Loss function . . . S19
S3.1.C Gradient of the Loss . . . S21
S3.1.D Workflow . . . S24
S3.2 Force Fields . . . S26
S3.3 MD Simulation . . . S26
S3.4 Comparison of Force Fields . . . S29
S3.4.A Density and PMV . . . S29
S3.4.B RDF and Coordination Numbers . . . S30
S3.5 Chemical Graphs . . . S34
S3.5.A Construction of Chemical Graphs . . . S34
S3.5.B Intracluster Statistics . . . S35
S3.5.C Topology of Clusters . . . S36
S3.6 Laguerre Tessellation . . . S37
S3.6.A Background . . . S37
S3.6.B PMV For Clusters . . . S38
S3.6.C Sensitivity to the choice of Hyperparameters . . . S42
S3.7 Normal Mode Raman Spectra . . . S45
S3.7.A Evolution of the Hydrogen Bond Network for Water . . . S45
S3.7.B Clusters for Raman Calculations . . . S47
S3.7.C Frequency Calibration . . . S47

References **S48**

S1 Experimental Density and Partial Molar Volume

The experimental procedures employed, along with the associated error analysis for density and partial molar volume (PMV) measurements of LiCl aqueous solutions, are summarized below.

S1.1 Density Measurement and Analysis for LiCl solution

Density measurements of aqueous LiCl solutions were performed using a nominal 50 mL pycnometer, calibrated to an effective volume of 53.372 ± 0.002 mL at 20.0°C. Ultrapure water (18.2 MΩ·cm) was obtained from a NANOpure Ultrapure Water System (Barnstead) as well as from a Milli-Q-UF-Plus system (Millipore) for solution preparation. LiCl (ACS reagent, ≥ 99%) was treated by baking at 580°C for 14 hours prior to use. Prepared solutions were filtered twice using syringe filters (VWR, 25 mm/0.22 μm) before measurements. All density measurements were conducted at 20.0°C, with the temperature monitored by a thermometer inside the pycnometer. This procedure minimized uncertainties associated with the volume calibration, concentration determination, temperature control, and salt purity. The overall uncertainty in the measured densities was less than ± 0.0005 g/mL across 14 data points obtained from three independent trials.

To determine the derivative of the density with respect to molality, $\left(\frac{\partial \rho}{\partial m}\right)$, polynomial fits of increasing order were applied to the density-molality profile and the residuals were systematically analyzed (Figure. S1.1). A linear fit (Figure. S1.1a) produced residuals exhibiting a pronounced quadratic trend exceeding the experimental uncertainty. Inclusion of a quadratic term (Figure. S1.1b) reduced this deviation but revealed a cubic feature that remained larger than the uncertainty. Upon incorporating a cubic term (Figure. S1.1c), the residuals fell entirely within the experimental uncertainty (± 0.0005 g/mL). Model selection based on the adjusted R^2 and the Bayesian Information Criterion (BIC)[1] further supported the necessity of at least a third-order polynomial to adequately describe the data. Both criteria penalize model complexity; the increase in the adjusted R^2 and the decrease in BIC upon inclusion of a degree of freedom indicates a genuine improvement in the fit that justifies the additional parameter. Although higher-order polynomials yielded marginal improvements in the statistical fit, these gains were smaller than the experimental uncertainty and were accompanied by increasing multicollinearity among polynomial terms, which inflates parameter uncertainties and compromises the reliability of statistical inference. Consequently, higher-order models were not considered further. The resulting cubic fit, comprised of the intercept, a linear, a quadratic, and a cubic term, was therefore used to evaluate the molality derivative for subsequent PMV determination. Notably, limiting the fit to a quadratic form - due to insufficient density precision - leads to inaccurate $\left(\frac{\partial \rho}{\partial m}\right)$ and PMV profiles (see Section S1.4 for comparison with previous experimental measurements from the literature).

The density profiles were fitted to a third order polynomial in molality, m, to determine the PMV :

$$\rho = A_0 + A_1 m + A_2 m^2 + A_3 m^3 \tag{S1.1}$$

The A_i parameters are summarized in Table S1.1.

Table S1.1: Average Fit Coefficients for the density profile with respect to molality

**Coefficient**	**$A_0 \times 10^{-1}$**	**$A_1 \times 10^{-2}$**	**$A_2 \times 10^{-4}$**	**$A_3 \times 10^{-5}$**
Value	9.985 ± 0.001	2.400 ± 0.009	−8.911 ± 0.120	2.701 ± 0.054

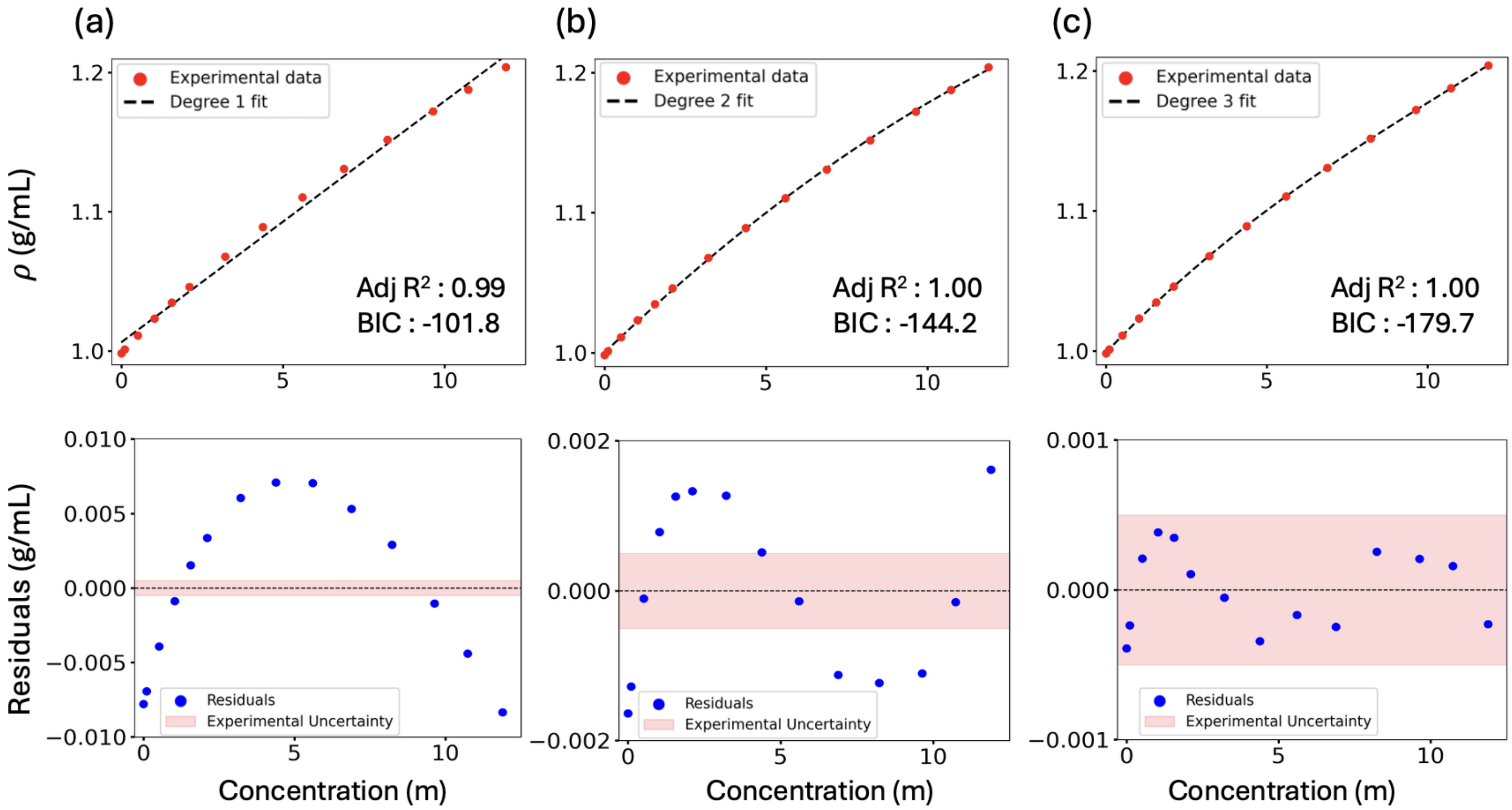

Figure S1.1: *The fitting and residual plots (top and bottom panels respectively) of LiCl solution densities with polynomials (a) linear , (b) quadratic , and (c) cubic in molality (left to right).*

The density profile can also be fitted to a third order polynomial in molarity [c] ($\rho = A'_0 + A'_1[c]^2 + A'_2[c]^2 + A'_3[c]^3$). The A'_i parameters are summarized in Table S1.2.

Table S1.2: Average Fit Coefficients for the density profile with respect to molarity

**Coefficient**	$\mathbf{A'_0 \times 10^{-1}}$	$\mathbf{A'_1 \times 10^{-2}}$	$\mathbf{A'_2 \times 10^{-4}}$	$\mathbf{A'_3 \times 10^{-5}}$
Value	9.984 ± 0.001	2.449 ± 0.009	-6.394 ± 0.136	3.469 ± 0.075

Polynomial fitting was performed independently for each of the three experimental datasets tabulated in Section S1.3. The reported coefficients correspond to the mean of the three independent fits, with uncertainties given as the standard error of the mean (SEM).

S1.2 Derivation and analysis of Partial Molar Volumes

The formal derivation of the Partial Molar Volume (PMV) using the thermodynamic density of a solution is outlined below.

S1.2.A Derivation of PMV

For a binary salt-water solution, the decompositions of volume contributions can be carried out in two ways: using the apparent molar volume or the PMV[2,3]. The more commonly reported apparent molar volume of the salt, (ϕ^{salt}), is defined as the effective per-mole volume contribution of the salt such that, when added to pure water (whose

molar volume, $\bar{V}_m^{H_2O}$, is multiplied by the number of moles of water) yields the total volume of a mixture at a given concentration (V_{sys}^{conc}) as:

$$V_{sys}^{conc} = n_{salt}\phi^{salt} + n_{H_2O}\bar{V}^{H_2O} \tag{S1.2}$$

where n_{H_2O} and n_{salt} are the number of moles of water and salt present in the solution at that concentration, respectively.

While convenient, the apparent molar volume is not the correct thermodynamic quantity. Its definition explicitly assumes that the molar volume of water remains fixed at all concentrations. This assumption is inaccurate as it neglects the strong ion-water interactions that lead to electrostriction and substantially modify the local structure and intrinsic molar volume of water.

To properly account for such concentration-dependent effects, one must consider the PMV, which is defined thermodynamically as the derivative of the total system volume with respect to the number of moles of a given component, taken at constant temperature, pressure, and fixed amounts of all other components. Unlike the apparent molar volume, the PMV does not assume an invariant solvent contribution; rather, it rigorously captures how the total volume responds to the incremental addition of a component within an interacting mixture. Mathematically, the partial molar volume of component i, V_m^i, is defined thermodynamically as:

$$V_m^i = \left(\frac{\partial V_{\text{sys}}}{\partial n_i}\right)_{T,P,n_{j\neq i}} \tag{S1.3}$$

and an equivalent equation to Equation S1.2 can be written for the PMV:

$$V_{sys}^{conc} = n_{salt}V_m^{salt} + n_{H_2O}V_m^{H_2O} \tag{S1.4}$$

with the distinction of $V_m^{H_2O}$ denoting the partial molar volume of water, which is itself a concentration dependent quantity. From these definitions, the partial molar volume of the salt and its apparent molar volume are therefore related through:

$$V_m^{salt} = \phi^{salt} + n_{salt}\left(\frac{\partial \phi^{salt}}{\partial n_{salt}}\right)_{T,P,n_{H_2O}} \tag{S1.5}$$

From this point onward, we focus exclusively on the thermodynamically rigorous PMV and express the concentration in terms of molality, m, defined as:

$$m = \frac{1000.n_{salt}}{n_{H_2O}M_{H_2O}} \tag{S1.6}$$

where the molar mass of water M_{H_2O} expressed in g/mol.

Given the total mass of the solution ($M_{sys}^{m} = n_{H_2O}M_{H_2O} + n_{salt}M_{salt}$), with the molar masses M_i of the components, and the corresponding density ($\rho = M_{sys}^{m}/V_{sys}^{m}$), where the superscript 'conc' are replaced with m to indicate the use of molality as the concentration scale, the PMV of the salt can be related to these measurable quantities through Equation S1.3 :

$$\begin{aligned} V_m^{salt} &= \frac{\partial}{\partial n_{salt}}\left[\frac{n_{salt}M_{salt} + n_{H_2O}M_{H_2O}}{\rho}\right]_{T,P,n_{H_2O}} \\ &= \frac{M_{salt}}{\rho} - \frac{(n_{salt}M_{salt} + n_{H_2O}M_{H_2O})}{\rho^2}\left(\frac{\partial \rho}{\partial n_{salt}}\right)_{T,P,n_{H_2O}} \end{aligned} \tag{S1.7}$$

Now, using the chain rule of derivatives, one can redefine the derivative $\left(\frac{\partial}{\partial n_{salt}}\right)$ as

$$\left(\frac{\partial}{\partial n_{salt}}\right) = \left(\frac{\partial m}{\partial n_{salt}}\right) . \left(\frac{\partial}{\partial m}\right) = \frac{1000}{n_{H_2O}M_{H_2O}}\left(\frac{\partial}{\partial m}\right) \tag{S1.8}$$

where by definition of the partial molar volume of the salt, the derivative is taken at constant n_{H_2O}. Substituting the corresponding expression for n_{salt} from inverting Equation S1.6 into Equation S1.7, and performing the change of variable from n_{salt} to m in the derivative term using Equation S1.8 yields:

$$\begin{aligned} V_m^{salt} &= \frac{M_{salt}}{\rho} - \frac{1000}{n_{H_2O}M_{H_2O}}\frac{(n_{salt}M_{salt} + n_{H_2O}M_{H_2O})}{\rho^2}\left(\frac{\partial \rho}{\partial m}\right) \\ &= \frac{M_{salt}}{\rho} - \frac{1000}{n_{H_2O}M_{H_2O}}\frac{(\frac{n_{H_2O}M_{H_2O}m}{1000}M_{salt} + n_{H_2O}M_{H_2O})}{\rho^2}\left(\frac{\partial \rho}{\partial m}\right) \\ &= \frac{M_{salt}}{\rho} - \frac{(1000 + mM_{salt})}{\rho^2}\left(\frac{\partial \rho}{\partial m}\right) \end{aligned} \tag{S1.9}$$

Similarly, the PMV for water, $V_m^{H_2O}$, follows:

$$\begin{aligned} V_m^{H_2O} &= \frac{\partial}{\partial n_{H_2O}}\left[\frac{n_{salt}M_{salt} + n_{H_2O}M_{H_2O}}{\rho}\right]_{T,P,n_{salt}} \\ &= \frac{M_{H_2O}}{\rho} - \frac{(n_{salt}M_{salt} + n_{H_2O}M_{H_2O})}{\rho^2}\left(\frac{\partial \rho}{\partial n_{H_2O}}\right)_{T,P,n_{salt}} \end{aligned} \tag{S1.10}$$

This can again straightforwardly be obtained by substituting the corresponding chain derivative:

$$\left(\frac{\partial}{\partial n_{H_2O}}\right) = \left(\frac{\partial m}{\partial n_{H_2O}}\right) . \left(\frac{\partial}{\partial m}\right) = -\frac{1000.n_{salt}}{n_{H_2O}^2 M_{H_2O}}\left(\frac{\partial}{\partial m}\right) = -\frac{m}{n_{H_2O}}\left(\frac{\partial}{\partial m}\right) \tag{S1.11}$$

into Equation S1.10 yielding the expression analogous to Equation S1.9 for water:

$$
\begin{aligned}
V_m^{H_2O} &= \frac{M_{H_2O}}{\rho} + \frac{m}{n_{H_2O}} \frac{(n_{salt} M_{salt} + n_{H_2O} M_{H_2O})}{\rho^2} \left(\frac{\partial \rho}{\partial m}\right) \\
&= \frac{M_{H_2O}}{\rho} + \frac{m}{n_{H_2O}} \frac{(\frac{n_{H_2O} M_{H_2O} m}{1000} M_{salt} + n_{H_2O} M_{H_2O})}{\rho^2} \left(\frac{\partial \rho}{\partial m}\right) \\
&= \frac{M_{H_2O}}{\rho} + \left(\frac{m M_{H2O}}{\rho^2}\right)\left(1 + \frac{m M_{salt}}{1000}\right)\left(\frac{\partial \rho}{\partial m}\right)
\end{aligned}
\tag{S1.12}
$$

The expressions above provide direct connections between the PMV of water and salt to the density of the solution at the given concentration. It is notable that these relationships are not exclusive to the LiCl aqueous solution and can be extended to any homogeneous binary solution to determine the PMV of each component. Moreover, note that the derivative of the density $(\partial\rho/\partial m)$ appears in both Equations S1.9 and S1.12 with a large pre-factor. Therefore, accurately capturing the curvature of the density-molality relationship becomes crucial.

S1.2.B PMV from Measured Densities and Error Analysis

The PMV profiles for both LiCl and water were computed using the Equations described above, and their features were subsequently analyzed by fitting to power series in molarity. As shown in Figure S1.2 and S1.3, truncating the series at the cubic term is insufficient to capture the full behavior of either V_m^{LiCl} or $V_m^{H_2O}$; the residuals from such fits exhibit a clear quartic (fourth-power) trend. Inclusion of a fourth-order term in the fit successfully reproduces the characteristic shape of both profiles, and the residuals are reduced to random fluctuations. As before, the adjusted R^2 and the BIC are provided for comparison.

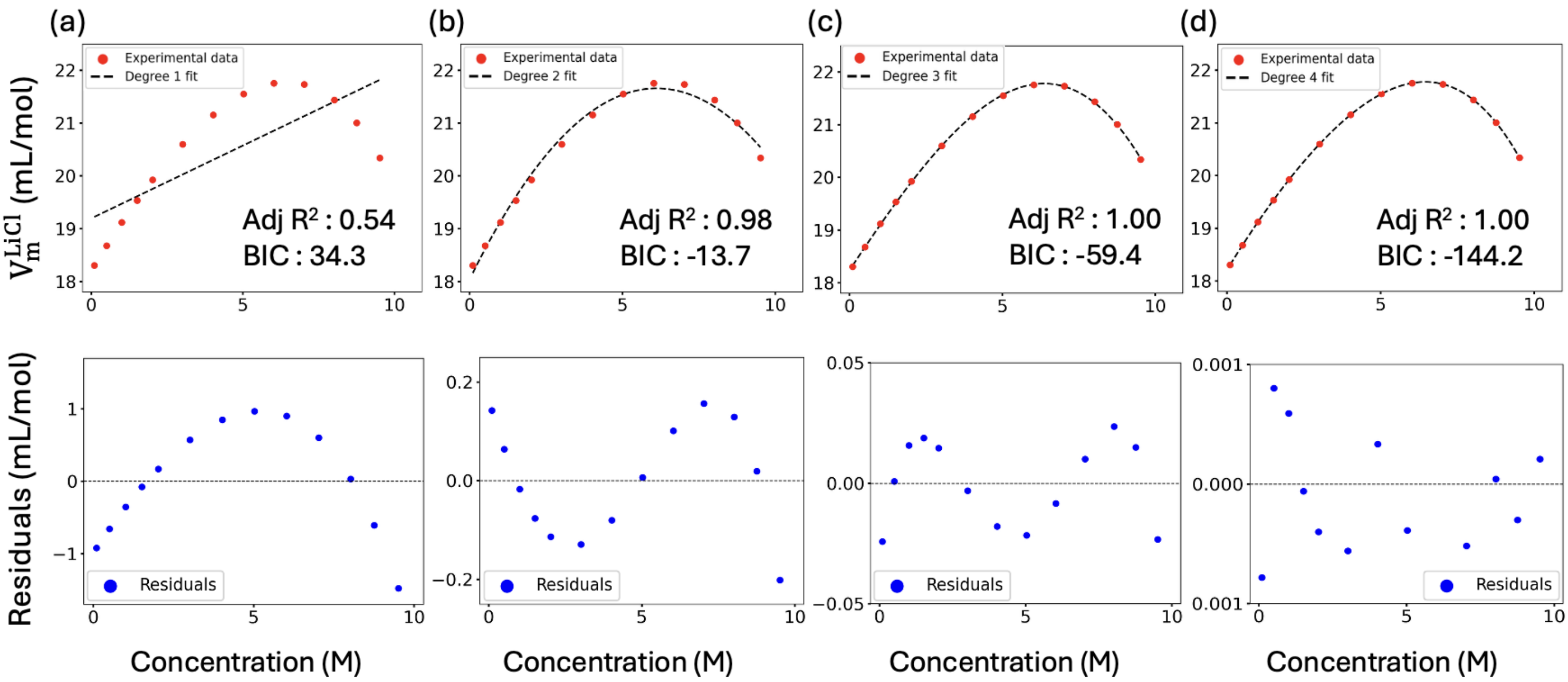

Figure S1.2: *The fitting and residual plots (top and bottom panels respectively) for Partial Molar Volumes of the LiCl with polynomials (a) linear, (b) quadratic, (c) cubic and (d) quartic in molarity (left to right).*

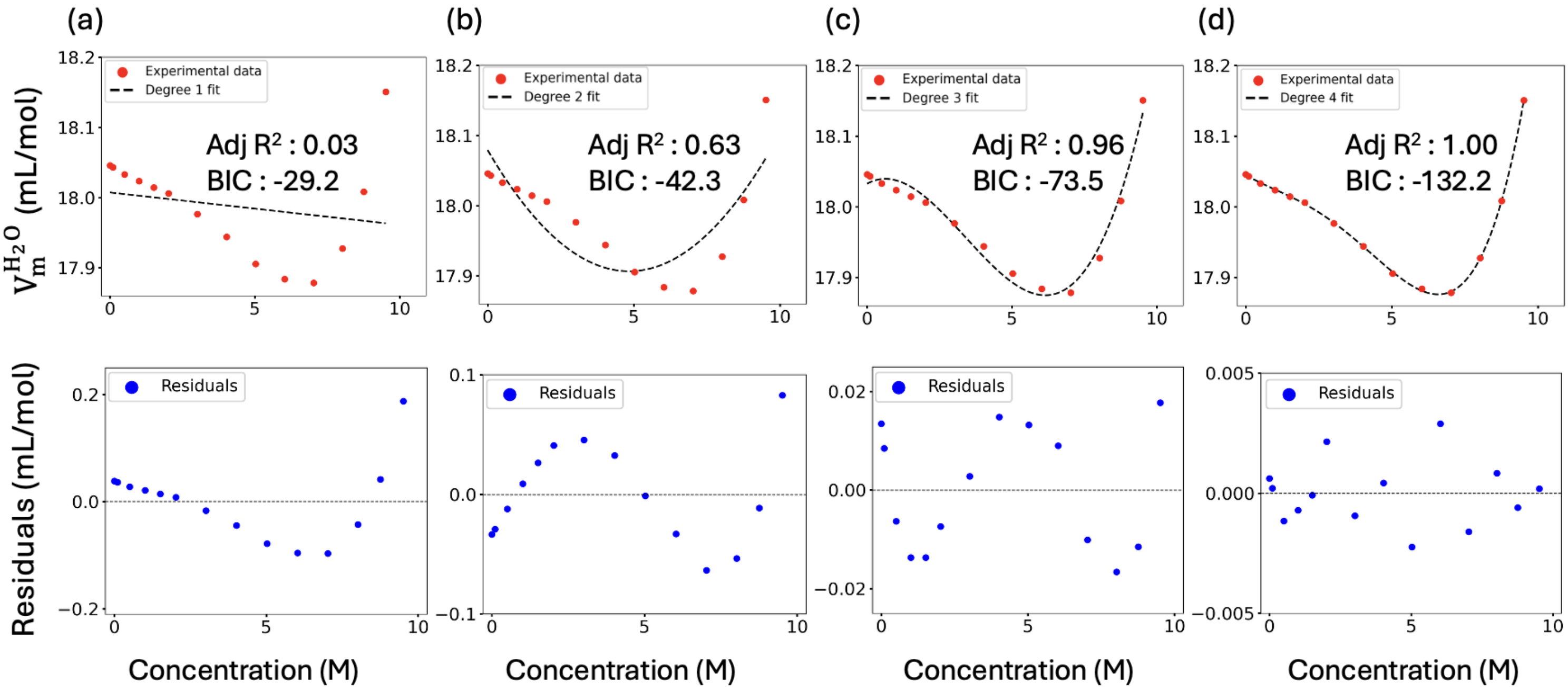

Figure S1.3: *The fitting and residual plots (top and bottom panels respectively) for Partial Molar Volumes of the water with polynomials (a) linear, (b) quadratic, (c) cubic and (d) quartic in molarity (left to right).*

Note that the signs of the fitting coefficients for the linear, quadratic, cubic, and quartic terms are opposite between V_m^{LiCl} and $V_m^{H_2O}$. This observation directly reflects the Gibbs-Duhem relationship in a binary system, which requires that the changes in the partial molar volume of one component is compensated by opposite changes in the other, maintaining the thermodynamic consistency of the system.

The PMV profiles were fit to a fourth-degree polynomial in molarity, as given in Equation. S1.13:

$$V_m = B_0 + B_1[c] + B_2[c]^2 + B_3[c]^3 + B_4[c]^4 \tag{S1.13}$$

The B_i parameters are summarized in Table S1.3.

Table S1.3: Average Fit Coefficients for the V_m profiles with respect to molarity

Coefficient	B_0	$B_1 \times 10^{-1}$	$B_2 \times 10^{-2}$	$B_3 \times 10^{-4}$	$B_4 \times 10^{-4}$
Value (V_m^{LiCl})	18.381 ± 0.088	9.077 ± 0.248	−5.056 ± 0.179	8.784 ± 0.809	−3.407 ± 0.086
Value ($V_m^{H_2O}$)	18.048 ± 0.002	−0.260 ± 0.007	0.743 ± 0.053	−27.067 ± 0.578	2.454 ± 0.023

Once again, polynomial fitting was performed using the PMV values computed independently for each of the three experimental datasets tabulated in Section S1.3. The reported coefficients correspond to the mean of the three independent fits, with uncertainties reported as the standard error of the mean (SEM).

S1.2.C Connecting the Density and PMV Coefficients

The coefficients A_i (from the density expansion) and B_i (from the V_m^{LiCl} expansion) can be interpreted analogously to a generalized virial expansion. In this framework, each term reflects the collective effect of particles

approaching one another and interacting either directly or indirectly through solvent-mediated perturbations of the local environment[4,5]. A key distinction arises from the fact that the density is a property of the system as a whole, whereas the PMV is a component-specific, derivative property that describes how the system's volume changes in response to variations in composition[2]. In the density expansion, the nth-order coefficient A_n represents the contribution arising from the simultaneous perturbation of n ions in solution, mediated by the solvent. For example, A_0 corresponds to the density of pure water (independent of ions), while A_1 captures the ion contribution to the density in the limit of infinite separation (i.e., fully solvated ions that do not interact with one another).

In contrast, because the PMV is defined as a derivative with respect to the number of moles of salt, the nth-order coefficient B_n effectively reflects interactions involving n+1 ions. Conceptually, the PMV can be viewed as a per-particle average: one may imagine selecting a single ion and evaluating how its volumetric contribution is modified by the presence of n additional ions in its vicinity. The derivative operation thus shifts the effective order of interaction by one.

In particular, the connection between the zeroth-order PMV coefficient B_0 (representing the one-body contribution of the salt in the infinite-dilution limit) and the linear density coefficient A_1, discussed in the main text, follows directly from introducing the expansions of the density and salt PMV in Equation S1.9[6]. In the limit of infinite dilution, this relationship can be obtained analytically as:

$$\begin{aligned}\lim_{c\to 0}\left(B_0 + B_1[c] + B_2[c]^2 + B_3[c]^3 + B_4[c]^4\right) = \lim_{m\to 0} & \frac{M_{\mathrm{LiCl}}}{A_0 + A_1[m] + A_2[m]^2 + A_3[m]^3} \\ & - \frac{1000 + mM_{\mathrm{salt}}}{(A_0 + A_1[m] + A_2[m]^2 + A_3[m]^3)^2} \\ & \times \left(\frac{\partial(A_0 + A_1[m] + A_2[m]^2 + A_3[m]^3)}{\partial m}\right)\end{aligned} \tag{S1.14}$$

$$\therefore\ B_0 = \frac{1}{A_0}\left\{M_{LiCl} - 1000\left(\frac{A_1}{A_0}\right)\right\} \tag{S1.15}$$

S1.3 Experimental Datasets

The density measurements from three independent experiments, together with the corresponding derived PMV values, are summarized in Table S1.4.

Table S1.4: Density and PMV data from three independent experiments

Dataset	Molarity (M)	Molality (m)	ρ (g/mL)	V_m^{LiCl} (mL/mol)	$V_m^{H_2O}$ (mL/mol)
A	0.0000	0.0000	0.9983	—	18.0459
	0.1015	0.1019	1.0009	18.3011	18.0430
	0.5056	0.5109	1.0110	18.6763	18.0329
	1.0097	1.0301	1.0230	19.1179	18.0236

Dataset	Molarity	Molality	ρ	V_m^{LiCl}	$V_m^{H_2O}$
	1.5151	1.5612	1.0347	19.5328	18.0146
	2.0244	2.1084	1.0460	19.9231	18.0060
	3.0181	3.2113	1.0678	20.5984	17.9766
	4.0237	4.3807	1.0891	21.1522	17.9442
	5.0294	5.6065	1.1103	21.5485	17.9056
	6.0302	6.8903	1.1308	21.7569	17.8839
	7.0295	8.2341	1.1517	21.7334	17.8784
	8.0232	9.6427	1.1722	21.4361	17.9276
	8.7623	10.7345	1.1877	21.0019	18.0084
	9.5184	11.8927	1.2039	20.3406	18.1507
B	0.0000	0.0000	0.9981	—	18.0488
	0.1009	0.1013	1.0007	18.5626	18.0466
	0.5021	0.5076	1.0105	18.9049	18.0388
	1.0042	1.0249	1.0224	19.3104	18.0287
	1.5071	1.5535	1.0340	19.6910	18.0187
	2.0090	2.0926	1.0452	20.0455	18.0091
	3.0142	3.2093	1.0670	20.6750	17.9854
	4.0160	4.3754	1.0881	21.1813	17.9574
	5.0139	5.5988	1.1093	21.5408	17.9223
	6.0181	6.8791	1.1300	21.7236	17.9002
	7.0156	8.2237	1.1505	21.6919	17.9035
	8.0138	9.6320	1.1717	21.3963	17.9400
	8.7507	10.7248	1.1869	20.9806	18.0255
	9.5133	11.8926	1.2032	20.3419	18.1626
C	0.0000	0.0000	0.9981	—	18.0490
	0.1011	0.1015	1.0006	18.5528	18.0480
	0.5027	0.5082	1.0105	18.8979	18.0389
	1.0051	1.0259	1.0223	19.3063	18.0311
	1.5078	1.5544	1.0339	19.6907	18.0205
	2.0102	2.0943	1.0451	20.0501	18.0123
	3.0142	3.2093	1.0670	20.6895	17.9852
	4.0149	4.3736	1.0882	21.2089	17.9537
	5.0152	5.5953	1.1090	21.5877	17.9224
	6.0116	6.8729	1.1295	21.7937	17.8975

Dataset	Molarity	Molality	ρ	V_m^{LiCl}	$V_m^{H_2O}$
	7.0142	8.2239	1.1503	21.7898	17.8938
	8.0098	9.6341	1.1710	21.5301	17.9317
	8.7503	10.7291	1.1865	21.1399	18.0029
	9.5054	11.8927	1.2022	20.5396	18.1387

S1.4 Comparison of the PMV and Density with Available Literature

The PMV and density profiles in the present work discussed in the main text were also compared below with those available in literature.

S1.4.A Partial Molar Volume

It has long been recognized that the addition of salts to water alters the total volume of the solution. During the late nineteenth and early twentieth centuries, Green and Wade[7] reported apparent molar volumes for sucrose, HCl, and LiCl in aqueous solutions. Volume changes upon adding salt and acid were also discussed by Nernst[8] in his classical treatments of solution thermodynamics. Gregory[9] subsequently initiated more systematic studies of alkali halides, focusing primarily on apparent molar volumes.

Lamer and Gronwall[10] were among the first to attempt analytical determination of partial molar volumes for both salts and water. However, their approach did not derive partial molar volumes from density measurements. Instead, they directly measured solution volumes at different concentrations and calculated V_m^{salt} from its original definition, $\left(\frac{\partial V_{sys}}{\partial n_{salt}}\right)$. Owing to experimental limitations in precise volume measurements and the absence of modern regression techniques, the resulting partial molar volumes were not sufficiently accurate. Studies of partial molar volumes for electrolyte solutions from 1927 to 2000 focused on the extrapolation of molar volumes at infinite dilution[11–14]. Moreover, most of the literature in this era used the apparent molar volume (ϕ^{salt})[15–18], which is different from the V_m^{salt} and does not correctly describe the solution thermodynamics. As illustrated by the black pentagons in Figure S1.4, the ϕ^{salt} derived from our density profile deviates from the real V_m^{salt} and does not exhibit a maximum.

A formally consistent connection between partial molar volume and density was not clearly established until 2001, when Klugman[19] derived expressions yielding comparable functional forms for the partial molar volumes of 1:1 electrolytes and water. However, the density datasets employed in that work lacked sufficient precision for accurate $\left(\frac{\partial \rho}{\partial m}\right)$ determination, leading to large deviations in the calculated V_m^{salt} for LiCl solutions (see the yellow squares in Figure S1.4). In subsequent years, the term *apparent molar water volume* (ϕ^{H_2O}) gained prominence in the study of Hofmeister effect[20] and water-in-salt lithium electrolytes[21]. Nevertheless, ϕ^{H_2O} obtained in this manner is thermodynamically inconsistent for an interacting binary system.

In the present work, we emphasize that the partial molar volumes of both components in a binary electrolyte solution are inherently concentration dependent. Only through rigorous thermodynamic derivation combined with high-precision density measurements, can accurate and physically meaningful PMV profiles for both salt and water be obtained.

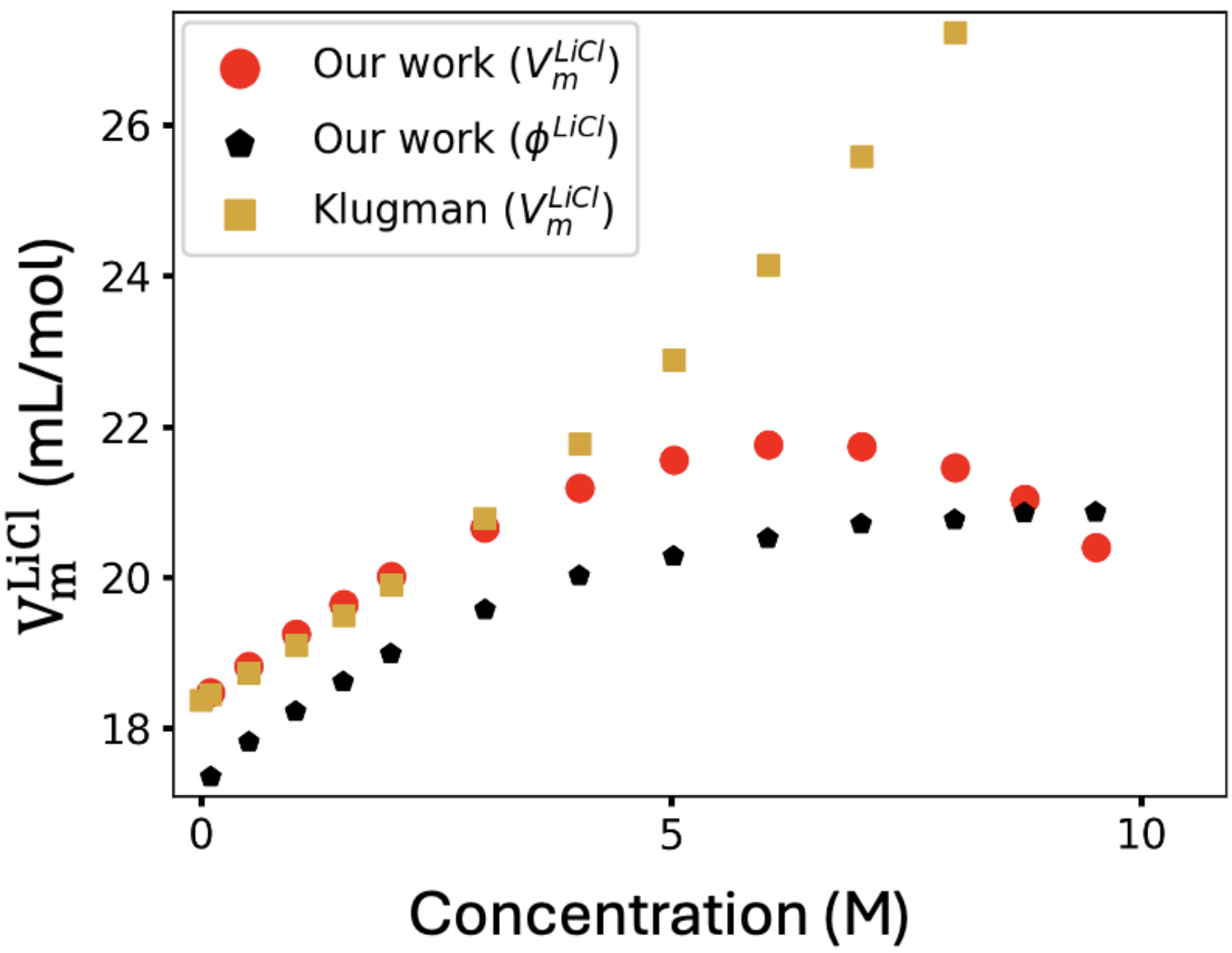

Figure S1.4: *Comparison of the PMV and apparent molar volume for LiCl between the present work and available literature (see text for details).*

S1.4.B Density

Although no accurate PMV profiles for the LiCl solution are available in the literature, we examined whether literature datasets of density could reproduce V_m^{LiCl} profiles comparable to our measurements when the correct thermodynamic derivation is applied. To this end, six different density sources by Wolf (1966)[22], Söhnel (1985)[23], Lobo (1989)[24] , Klugman (2001)[19], Vercher (2003)[25] and David (2005)[26] were selected for comparison. The reported density profiles were used to calculate V_m^{LiCl} values using the formalism described in Section S1.2.A.

As shown in Figure S1.5a, all seven density datasets - including ours - appear very similar upon visual inspection. However, the derived PMV profiles exhibited pronounced differences (Figure S1.5b). The datasets from Lobo and David yielded qualitatively comparable PMV trends. Wolf's dataset is reasonably accurate as well but only contained two data points beyond 4 M. As such, it failed to capture the maximum in V_m^{LiCl} near 6.7 M. It is also notable that Lobo's data systematically deviate below 2 M, while David's data exhibited larger differences above 3 M. In contrast, the V_m^{LiCl} profiles derived from Söhnel, Klugman, and Vercher displayed vastly different trends and magnitudes across the entire concentration range. Specifically, Söhnel's increased monotonically, while Klugman's diverged strongly after 3 M. Moreover, Vercher's profile showed a minimum rather than a maximum. It should be noted that more erroneous data can be found if one also included data from earlier in the 20th century[27,28].

These comparisons demonstrate that accurate density profiles for LiCl are indeed available in the literature. However, one needs to identify properly measured and curated datasets. On the other hand, accurate density profiles alone are insufficient to yield reliable PMV profiles unless the correct thermodynamic derivation is applied. Without this rigorous treatment, discrepancies in PMV can arise despite apparently consistent density values. Furthermore, improper derivation obscures the ability to critically evaluate density datasets, making it difficult to detect systematic errors, identify outliers, and ensure reliable parameterization. In conclusion, a good density profile that is capable for deriving reliable PMV requires (1) preparing the solution at every concentration in terms of mass instead of volume (2) measuring more data points at low concentration (< 2 M) to construct the linear

trend, (3) containing at least a data point at every molar after 2 M, and (4) repeating each data point three times or more to get the error bars at every concentration.

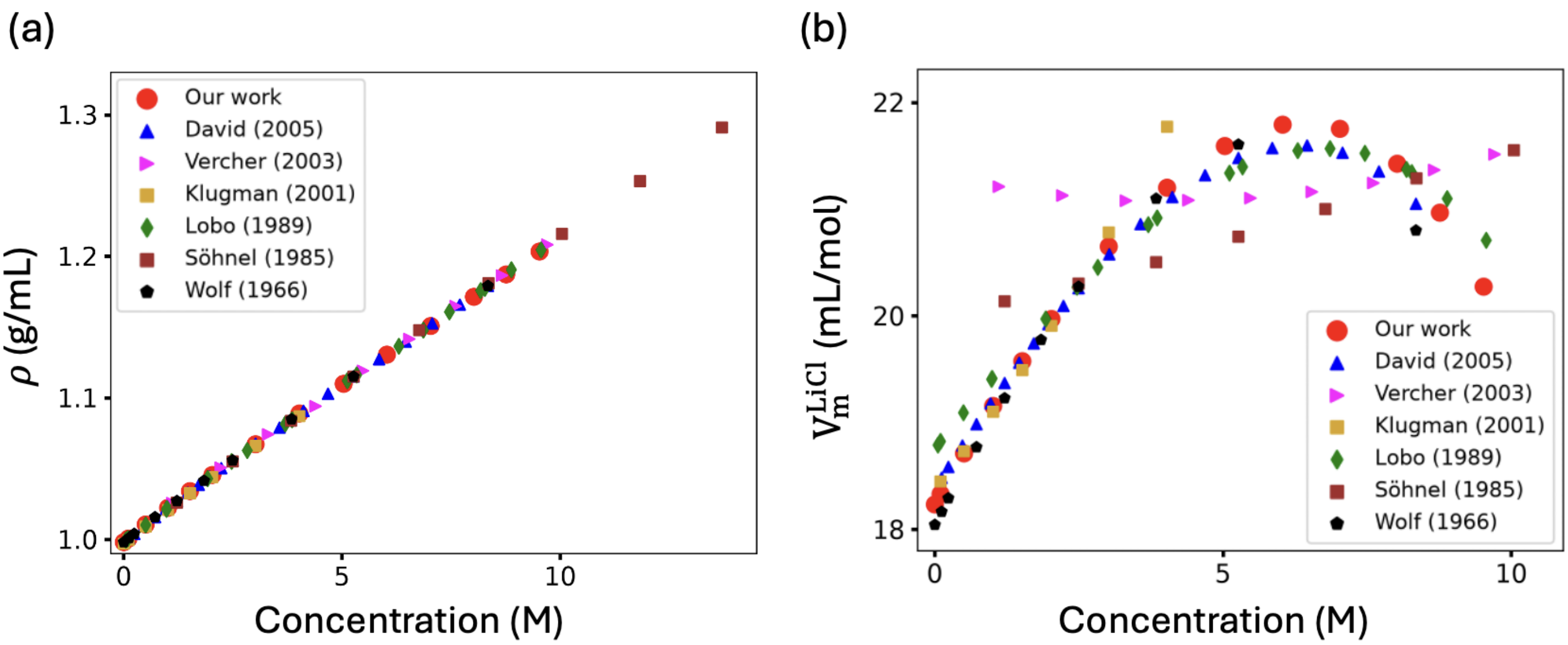

Figure S1.5: *Comparison of the (a) density and (b) PMV for LiCl (derived from the density) between the present work and available literature.*

Finally, when plotting density and PMV profiles versus increasing concentration, molality should be converted to molarity, as molality is not the proper thermodynamic parameter to account for volume changes that occur upon the addition of solute. In the current work, all density and PMV data were acquired/analyzed at constant temperature and pressure. Under these conditions, molarity is the preferred thermodynamic concentration unit. This preference follows from statistical mechanical considerations, as discussed in detail by Arieh Ben-Naim[29]. As he shows, even when pressure and temperature are varied, molarity rather than molality is still the preferred unit. In fact, as he notes, molality is a laboratory convenience.

S1.5 PMV of Water, Eutectic Point and Vapor Pressure of LiCl Solutions

As discussed in the main text, aggregation processes shape both the minimum in $V_m^{H_2O}$ and the characteristic features in the phase diagram of the aqueous LiCl solution. Figure S1.6a shows that the eutectic point in the LiCl(aq) phase diagram occurs at approximately 6.8 M[30], marking a transition from a regime dominated by the decreasing chemical potential of water to one governed by the rising chemical potential of LiCl. To further probe the behavior of water in these solutions, vapor pressure measurements of LiCl solutions across different concentrations from the literature[31] are presented in Figure S1.6b. As the vapor pressures were obtained at constant temperature and pressure, this dataset is directly comparable to $V_m^{H_2O}$. Addition of LiCl increases electrostriction, decreasing the chemical potential of water and its vapor pressure; however, this behavior is not monotonic. Analysis of the first derivative of the vapor pressure fit reveals a minimum near 6.9 M (Figure S1.6c), indicating that beyond this concentration the decrease in the chemical potential of water becomes less steep. This observation aligns with the claim that aggregation of LiCl at high concentration reduces the number of water molecules constrained within free ion hydration shells. Consequently, while the chemical potential of water continues to decrease as the salt concentration increases, the rate of this decrease becomes less pronounced above the aggregation threshold. Similarly, $V_m^{H_2O}$ increases beyond approximately 6.7 M, consistent with reduced electrostriction as more water molecules are liberated from direct interaction with free ions. The eutectic point, $V_m^{H_2O}$, and the vapor pressure

data for $LiCl_{(aq)}$ should be interconnected because each measurement effectively isolates and probes the behavior of the water component within the solution rather than that of the bulk mixture.

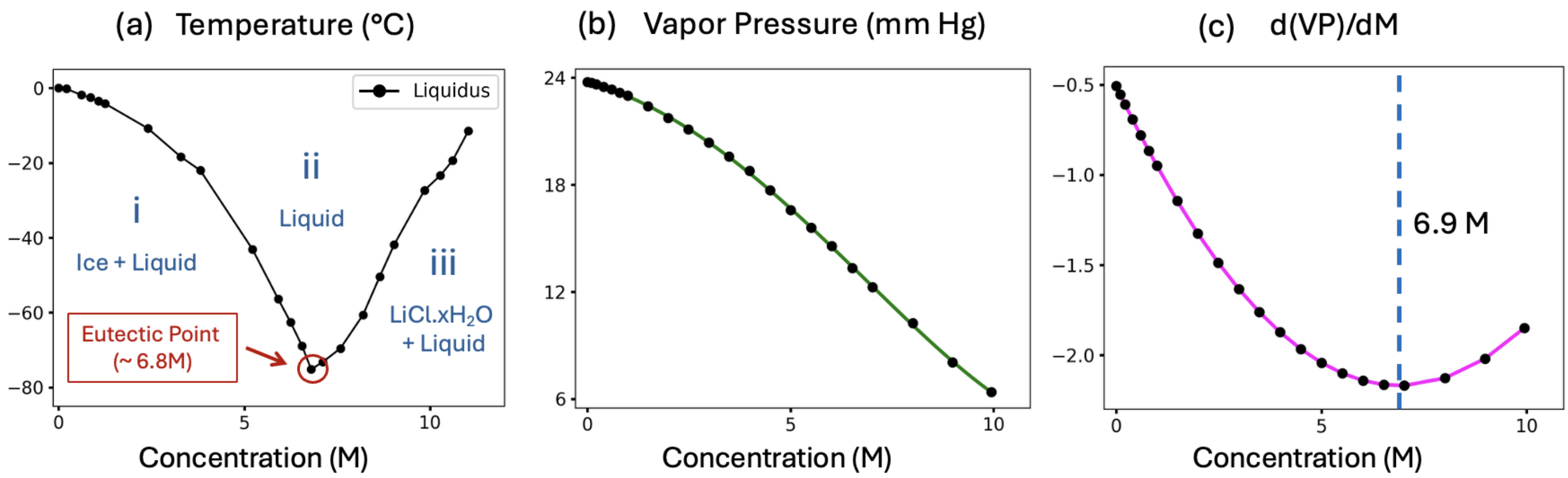

Figure S1.6: *(a) Phase diagram of LiCl in the aqueous solution (temperature vs molarity) demonstrating the eutectic point, (b) vapor pressure of LiCl solutions, and (c) derivative of the vapor pressure vs molarity.*

S2 Raman MCR Measurements

The experimental protocols for Raman measurements and the subsequent analysis of the spectra are described below.

S2.1 Raman Spectra of LiCl solutions and MCR analysis

To probe the structural and vibrational properties of water molucules in LiCl solutions, Raman spectra were collected using a home-built spectrometer. This section outlines the instrument design, experimental conditions, and the theoretical methods employed to extract solute-correlated water contributions from the measured spectra.

S2.1.A Instrumentation

A home-built Raman spectrometer was used to acquire spectra of aqueous LiCl solutions. For spectra in the range from 0 to 9.5184 M LiCl in H_2O, a 640 nm, 100 mW laser (CrystaLaser, Reno, NV, USA) excitation line was used. The power and polarization of the laser line were controlled by two half-wave plates (1200-15; CVI Laser, Albuquerque, NM) positioned on either side of a beam splitter (CCM1-PDBS252; Thor Labs, Newton, NJ). Highly reflective mirrors (BB1-E02P; ThorLabs) and a 638 nm Ultrasteep long-pass filter (LP02-638RE-25; Semrock) directed the beam to the sample. For Raman spectra with H_2O in D_2O and 9.5184 M LiCl/H_2O/D_2O solution, the excitation source was a Cobolt Fandango 515 nm 100 mW laser (HUBNER Photonics Inc. San Jose, USA). The power and polarization of the laser line were controlled by two half-wave plates (1200-15; CVI Laser, Albuquerque, NM) positioned on either side of a beam splitter (CCM1-PDBS252; Thor Labs, Newton, NJ). Highly reflective mirrors (BB1-E02P; ThorLabs) and a 514 nm Stopline single-notch filter (NF03-514E-25; Semrock) directed the beam to the sample.

The laser was focused onto a 1 cm quartz cuvette (5061-3387; Agilent Technologies) housed in a temperature-controlled jacket (Versa 20; Quantum Northwest, Liberty Lake, WA) using a 10x objective (LU Plan, WD = 17.3 mm, NA = 0.3; Nikon, Japan). Backscattered, unpolarized Raman signals were collected and passed through two Ultrasteep long-pass filters (for 640 nm laser) or Stopline Single-notch filters (for 515 nm laser) before being focused by a lens (AC254-050-A; Thor Labs) onto a custom 1 *x* 15 fiber-optic bundle (FiberTech-RoMack, Williamsburg, VA) coupled to a spectrometer (IsoPlane SCT 320; Princeton Instruments, Trenton, NJ). A 300 g/mm grating for 640 nm laser and a 600 g/mm grating for 515 nm laser dispersed the light onto a liquid-nitrogen-cooled 100 *x* 34 CCD array detector (Pylon-100BR; Princeton Instruments).

The power of the incident light at the sample stage was adjusted to 50 mW for all spectra. The exposure time for the CCD camera was 0.1 s, and the number of exposures was 2400 per frame. All measurements were performed at 20.0°C.

S2.1.B Theory and Spectral Analysis

Raman spectra were collected for twelve LiCl concentrations spanning from 0.5000 to 9.5184 M. The spectral contribution associated with "perturbed water" was subsequently extracted using multivariate curve resolution (MCR) analysis. The MCR decomposition was performed with an algorithm developed by Dor Ben-Amotz[32,33], which enables separation of overlapping spectral components based on concentration-dependent variations.

In this approach, the input spectral data matrix (D) is expressed as a linear combination of three components described by a concentration profile matrix (C) and a corresponding Raman signal matrix (S) : the bulk water

contribution ($C_{BW}S^T_{BW}$), the solute-correlated (perturbed water) contribution ($C_{SC}S^T_{SC}$), and a residual noise matrix (E) :

$$D = C_{BW}S^T_{BW} + C_{SC}S^T_{SC} + E \tag{S2.1}$$

The solute-correlated (SC) contribution in the 3000–3800 cm^{-1} region corresponds to OH oscillators whose vibrational frequencies are perturbed by the presence of LiCl in solution. As shown in Figure S2.1a, the intensity of the perturbed water spectrum increases systematically with increasing LiCl concentration. To further elucidate the speciation of OH oscillators in highly concentrated solutions, the SC spectra of LiCl were decomposed into three Gaussian components (Figure S2.1b). These components are assigned to an ensemble of OH oscillator populations: ordered, less ordered, and weakly hydrogen-bonded environments. Strong hydrogen bonding weakens and elongates the OH bond, causing a red shift in the vibrational frequencies, whereas disruption strengthens and shortens the bond, shifting the frequency toward the vacuum-like limit of an isolated OH oscillator[34–36].

To verify that these spectral features arise from physically distinct water species - rather than from inter- or intramolecular OH vibrational coupling - we performed isotopic dilution experiments to minimize OH oscillator coupling. Specifically, a 9.5184 M LiCl solution was prepared using a solvent mixture of H_2O in D_2O (H_2O:D_2O = 1:9 v/v), and the SC spectrum was obtained relative to the corresponding H_2O-in-D_2O solvent baseline. As shown in Figure S2.1c, the SC spectra contain OH (3000-3800 cm^{-1}) and OD (2000-2800 cm^{-1}) stretch bands. Under these dilution conditions, only ∼1% of OH oscillators are statistically likely to couple with another OH oscillator, effectively suppressing the OH vibrational coupling effects.

If any of the peaks observed in Figure S2.1b originated from OH coupling, they would be significantly diminished or absent in the isotopically diluted SC spectrum. However, as shown in Figure S2.1d, the OH stretching region of the diluted system still resolves into three Gaussian components. This confirms that the three Gaussian components in the SC spectrum were not artifacts of vibrational coupling but contained information on distinct ion-induced hydrogen-bonding environments.

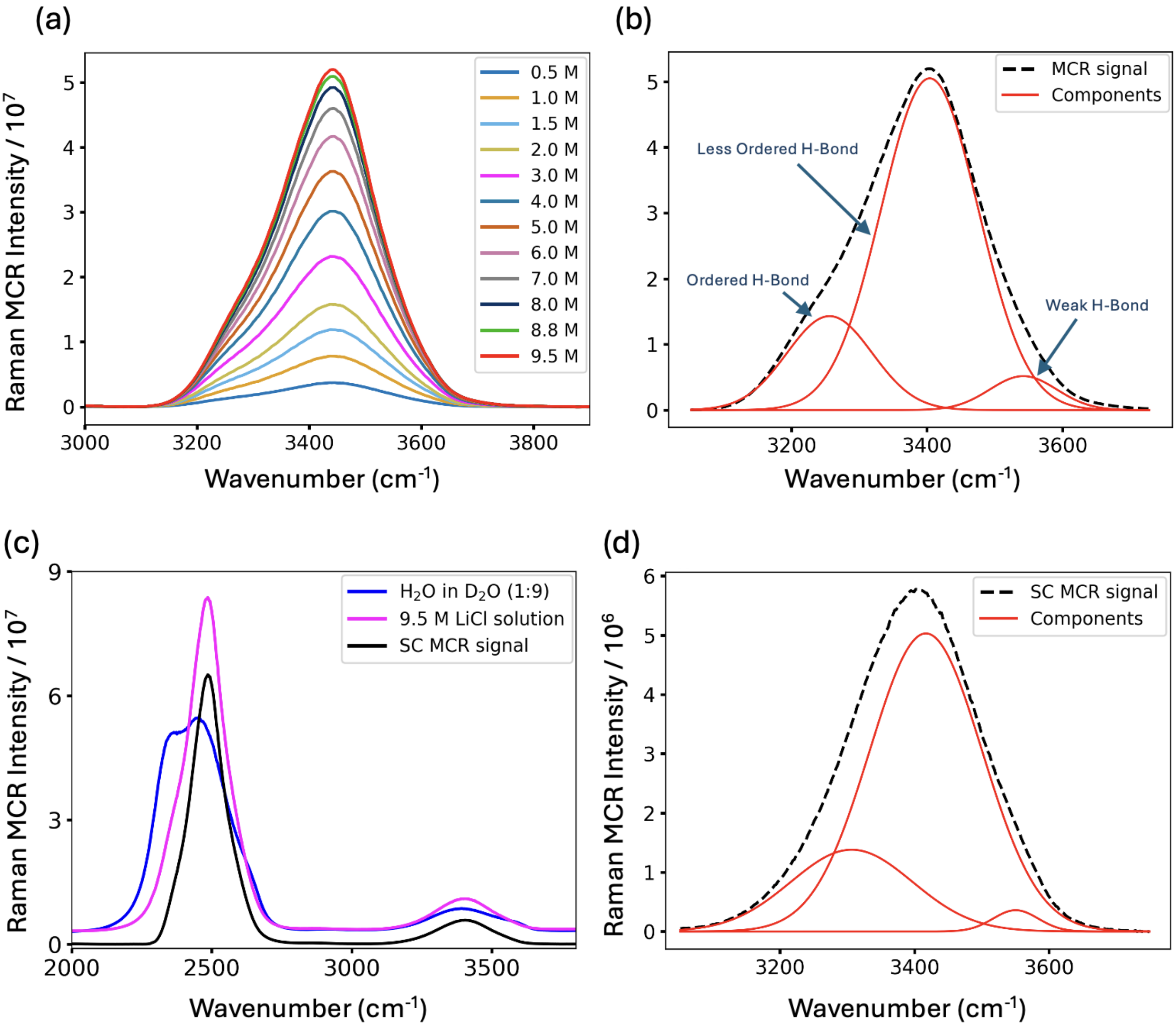

Figure S2.1: *(a) Solute Correlated (SC) spectrum of LiCl solutions across concentrations in H_2O solvent, (b) deconvolution of the SC spectrum into three Gaussian components, (c) SC spectrum of the LiCl solution in a solvent mixture of H_2O in D_2O. The crude Raman spectrum of the solution and the solvent mixture are also shown. (d) deconvolution of the isotopically diluted SC in OH stretching region into Gaussian components. It shows that the three components are preserved.*

A plausible alternative for analyzing spectra is to normalize the Raman-MCR spectra by their concentrations. In Figure S2.2, the concentration-normalized Raman-MCR spectra for 0.5, 4, 8 M LiCl solutions are plotted together for comparison. However, even though both the 3270 and 3590 cm^{-1} peaks grow with concentration (see Figure 7 in the main text), such growth cannot be easily visualized in the concentration-normalized spectra. This is because the intensity changes and band shifts for the 3270 and 3590 cm^{-1} features are dwarfed by the much larger peak at 3440 cm^{-1}. Moreover, the growth in the 3440 cm^{-1} peak levels off after 6.7 M, which causes the concentration-normalized spectrum at 8 M to show the smallest overall intensity. As a consequence, the 3270 and 3590 cm^{-1} peak changes do not appear by eye to represent their actual intensity increases and shifts. As such, one first needs to perform MCR fitting to properly reveal the changes in the two smaller peaks that actually occur.

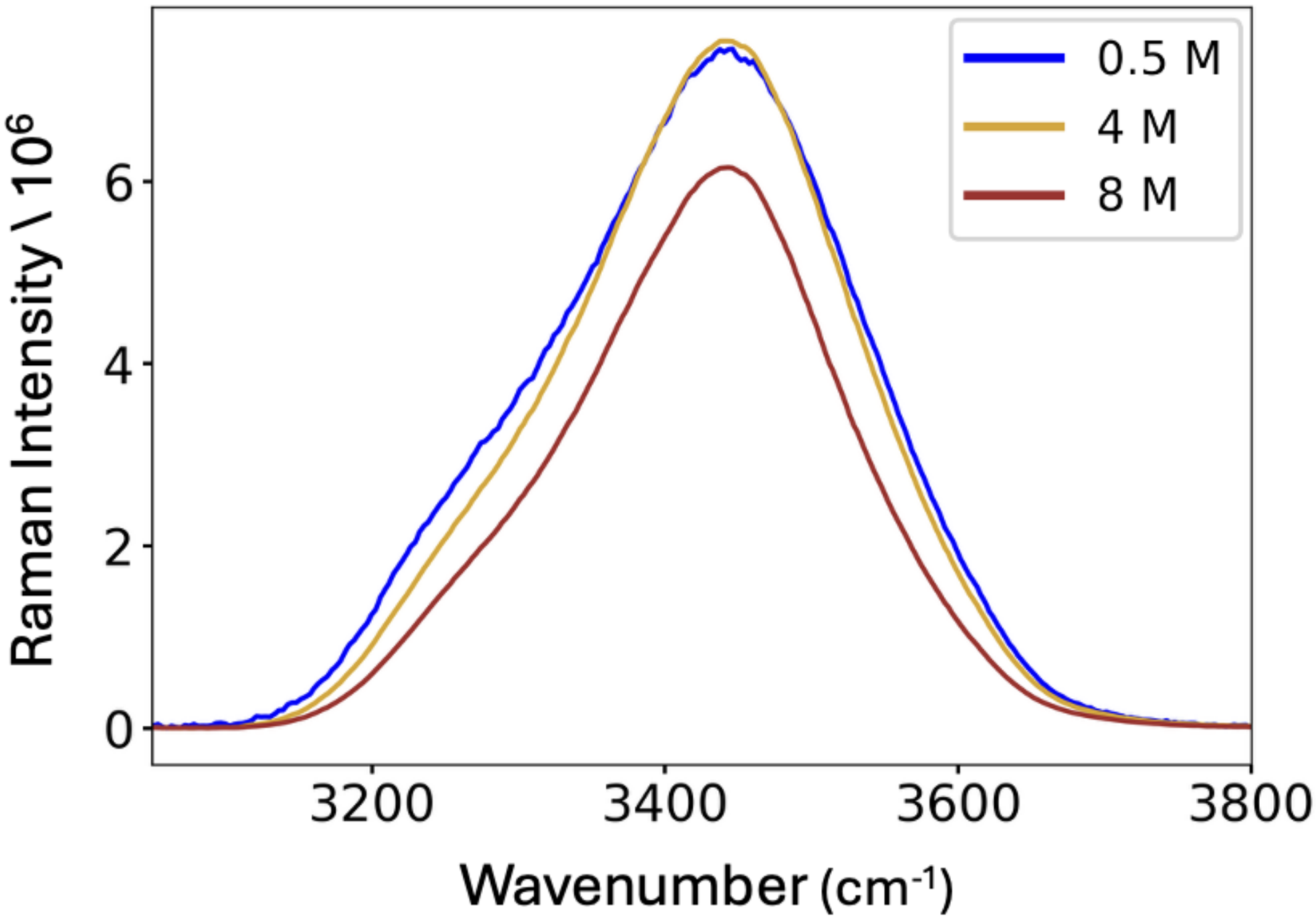

Figure S2.2: *Concentration-normalized Raman Spectrum for 0.5, 4 and 8 M LiCl solutions.*

S3 Computational Methods

This section details the range of computational methods employed including the classical molecular simulations as well as the protocol employed to optimize the force-fields (FF) and finally, the quantum-chemistry calculations of Raman spectra using density functional theory (DFT).

S3.1 Force-Field Optimization

The theoretical framework underlying force-field optimization is outlined below.

S3.1.A Background

This work utilizes the Joung-Cheatham (2008)[37] ion force field parameters - specifically those parameterized for TIP4P-Ew water, shown to also perform well with TIP4P/2005[38] (hereafter referred to as **JC**), and the more recent Madrid[39] (2019) parameters (hereafter referred to as $\mathbf{M_{19}}$) in conjunction with the TIP4P/2005[40] water model. These force-fields were benchmarked and subsequently optimized against the measured experimental density and derived PMV. In these force-field models, the N-particle interaction potential is approximated as a pairwise sum of terms involving a Lennard Jones (LJ) potential - and an electrostatic contribution. For a pair of particles α and β, the potential for the pair $U_{\alpha\beta}$ is given as:

$$\begin{aligned} U_{\alpha\beta}(r_\alpha, r_\beta) \;=\; U_{LJ} + U_{coul} &= 4\epsilon_{\alpha\beta}\left[\left(\frac{\sigma_{\alpha\beta}}{r_{\alpha\beta}}\right)^{12} - \left(\frac{\sigma_{\alpha\beta}}{r_{\alpha\beta}}\right)^{6}\right] + \left[\frac{q_\alpha q_\beta}{4\pi\epsilon_0 r_{\alpha\beta}}\right] \\ &= \left[\left(\frac{C_{12}^{\alpha\beta}}{r_{\alpha\beta}^{12}}\right) - \left(\frac{C_{6}^{\alpha\beta}}{r_{\alpha\beta}^{6}}\right)\right] + \left[\frac{q_\alpha q_\beta}{4\pi\epsilon_0 r_{\alpha\beta}}\right] \end{aligned} \tag{S3.1}$$

Here the parameters $\epsilon_{\alpha\beta}$ denote the LJ potential's energy depth while $\sigma_{\alpha\beta}$ is the distance at which the LJ potential crosses zero. $q_{\alpha\beta}$ are the respective partial charges and ϵ_0 is the vacuum permittivity. Note that the LJ term can equivalently be written in terms of $C_6^{\alpha\beta}(= 4\epsilon_{\alpha\beta}\sigma_{\alpha\beta}^6)$ and $C_{12}^{\alpha\beta}(= 4\epsilon_{\alpha\beta}\sigma_{\alpha\beta}^{12})$, which renders the LJ potential linear in these parameters (as shown in Equation S3.1). This convention is adopted to simplify subsequent derivations.

In the present study, the LJ self-interaction parameters ($\epsilon_{\alpha\alpha}$ and $\sigma_{\alpha\alpha}$) of Li^+ and Cl^- ions were adjusted, while the partial charges and water model parameters were kept unchanged. In addition, while the results in the main text focused on using integer charges for the ions, we also conducted some analysis (see section S3.4) with scaled charges.

S3.1.B Loss function

Previous works by the groups of Hummer[41,42] and Bussi[43–45] have rigorously formulated complete Bayesian frameworks that couple ensemble refinement with systematic force field optimization. In this approach, the Bayesian update scheme incorporates available experimental data as constraints to modify an existing prior distribution of configurations generated from sampling the appropriate ensemble, ω_0.

For the choice of target property, the density rather than the PMV was selected. Since PMV is already obtained from the derivative of the density with respect to concentration (Section S1.2.A), an accurate representation of the density

trend effectively provides PMV without requiring an additional direct fit. Moreover, the expression used to compute the PMV (Equation. S1.9) involves a large derivative coefficient, which can amplify small deviations and lead to numerical instability during optimization if PMV is fitted directly. Accurately reproducing the density therefore offers a more stable and physically transparent route, from which the PMV can be reliably derived without further adjustment.

The simulated densities were obtained for validation from molecular dynamics simulations performed in the NPT ensemble of aqueous LiCl at varying concentrations (details in Section S3.3), generated using the potential being refined. To enable efficient parameter optimization without repeating simulations, ensemble reweighting is employed as discussed previously[41–45]. Although modifications of the force field parameters alter the underlying potential energy landscape and thus the NPT distribution (ω_0) , where ω_0^i represents the weight assigned to the ith frame to recover ensemble averages (and is uniform, $\omega_0 = 1/N_{frames}$, for the unbiased simulations performed here), as long as the change from the initial force field parameters, and hence the resulting change in the potential energy (ΔU) remains small - reweighting allows the estimation of new ensemble averages by recycling the existing trajectory:

$$\langle \rho_{sim}^{conc} \rangle_\Delta = \frac{1}{\sum_{j=1}^{N_{frames}} e^{-\beta \Delta U_j}} \sum_{i=1}^{N_{frames}} \rho_i e^{-\beta \Delta U_i} = \sum_{i=1}^{N_{frames}} \rho_i \frac{e^{-\beta \Delta U_i}}{Z_\Delta} = \sum_{i=1}^{N_{frames}} \omega^i \rho_i \tag{S3.2}$$

Here, Z_Δ is the effective partition function over the perturbed energies, depending only on the force-field parameter changes since the same trajectory is reused. Finally, the terms $\left(\frac{e^{-\beta \Delta U_i}}{Z_\Delta}\right)$ are collected as weights ω^i.

To evaluate ΔU efficiently, we define for a given frame i, the pairwise sum of inverse distance exponents between all particles belonging to a given α-β pair as $\zeta_{6/12}^{\alpha\beta;i} = \sum_{x,y\in[\alpha,\beta]} \frac{1}{r_{x,y;i}^{6/12}}$. The resulting change in the LJ potential energy can then be written straightforwardly in terms of these geometric accumulators and $C_{6/12}^{\alpha\beta}$ from Equation S3.1 as :

$$\Delta U = \sum_{\alpha,\beta} \left[\Delta C_{12}^{\alpha\beta} \zeta_{12}^{\alpha\beta} - \Delta C_6^{\alpha\beta} \zeta_6^{\alpha\beta} \right] \tag{S3.3}$$

The resulting optimization is cast as the minimization of a loss function ($\mathcal{L}$) of the form:

$$\mathcal{L} = \sum_{conc} \left[\frac{(\langle \rho_{sim}^{conc} \rangle - \rho_{expt}^{conc} + \Delta\rho_{pure})^2}{2\sigma_{expt}^2} - \theta S_{rel}^{conc} \right] = \frac{1}{2}\chi^2(\omega) - \theta S_{rel}(\omega|\omega_0) \tag{S3.4}$$

where the 1st (χ^2) term represents the deviation of the simulated density at a given concentration from the experimental value and σ_{expt} is the experimental error. A correction ($\Delta\rho_{pure}$) is included, as changing the ion parameters wouldn't correct for any discrepancy in the density of pure TIP4P/2005 water. The 2nd term, the relative entropy ($S_{rel} = -\sum_i \omega^i log[\omega^i/\omega_0^i]$), denotes the deviation from the prior ensemble. This term constrains the optimization, preventing excessive deviation from the initial force field and ensuring sufficient ensemble overlap - a key requirement for efficient reweighting. Taken together, the optimization of $\mathcal{L}$ moves the ϵ and σ in a direction that reduces χ^2 while remaining anchored to the initial ensemble. The balance between the two is controlled by the hyperparameter θ. The scheme used to obtain θ is discussed in section S3.1.D.

S3.1.C Gradient of the Loss

Equipped with the above relations, the gradient of $\mathcal{L}$ with respect to the independent parameters can be obtained analytically for use in the optimization algorithm. The gradients are evaluated separately for the χ^2 and S_{rel} terms, as illustrated below for C_{12} (an identical form applies to C_6).

- **χ^2 part**:

$$\frac{\partial \chi^2}{\partial C_{12}^{\alpha\beta}} = \sum_{conc}\left[\frac{\langle \rho_{sim}^{conc}\rangle - \rho_{expt}^{conc} + \Delta\rho_{pure}}{\sigma_{expt}^2}\right]\left(\frac{\partial \langle \rho_{sim}^{conc}\rangle}{\partial C_{12}^{\alpha\beta}}\right) \tag{S3.5}$$

$$\text{where} \quad \frac{\partial \langle \rho_{sim}^{conc}\rangle}{\partial C_{12}^{\alpha\beta}} = \sum_{i=1}^{N_{frames}} \rho_i^{conc}\left(\frac{\partial \omega^i}{\partial C_{12}^{\alpha\beta}}\right) \tag{S3.6}$$

Now the derivative of the weights w.r.t the parameters are obtained as:

$$\begin{aligned}\frac{\partial \omega^i}{\partial C_{12}^{\alpha\beta}} &= \frac{1}{Z_\Delta^2}\left[Z_\Delta e^{-\beta\Delta U_i}(-\beta)\left(\frac{\partial \Delta U_i}{\partial C_{12}^{\alpha\beta}}\right) - e^{-\beta\Delta U_i}\sum_{j=1}^{N_{frames}} e^{-\beta\Delta U_j}(-\beta)\left(\frac{\partial \Delta U_j}{\partial C_{12}^{\alpha\beta}}\right)\right] \\ &= -\beta\omega^i\left[\frac{\partial \Delta U_i}{\partial C_{12}^{\alpha\beta}} - \left\langle \frac{\partial \Delta U}{\partial C_{12}^{\alpha\beta}}\right\rangle\right]\end{aligned} \tag{S3.7}$$

Using Equation S3.7 in S3.6 :

$$\frac{\partial \langle \rho_{sim}^{conc}\rangle}{\partial C_{12}^{\alpha\beta}} = -\beta\left[\left\langle \rho\, \frac{\partial \Delta U}{\partial C_{12}^{\alpha\beta}}\right\rangle - \langle\rho\rangle\left\langle \frac{\partial \Delta U}{\partial C_{12}^{\alpha\beta}}\right\rangle\right] \tag{S3.8}$$

- **S_{rel} part**:

$$S_{rel}^{conc} = -\sum_{i=1}^{N_{frames}} \omega^i \log\left(\frac{\omega^i}{\omega_0^i}\right) \tag{S3.9}$$

With a fixed starting distribution (ω_0), one obtains the derivative as:

$$
\begin{aligned}
\frac{\partial S_{rel}^{conc}}{\partial C_{12}^{\alpha\beta}} &= -\sum_{i=1}^{N_{frames}} \left[1 + \log\left(\frac{\omega^i}{\omega_0^i}\right)\right]\left[\frac{\partial \omega^i}{\partial C_{12}^{\alpha\beta}}\right] \\
&= \beta \sum_{i=1}^{N_{frames}} \left[\omega^i + \omega^i \log\left(\frac{\omega^i}{\omega_0^i}\right)\right]\left[\frac{\partial \Delta U_i}{\partial C_{12}^{\alpha\beta}} - \left\langle \frac{\partial \Delta U}{\partial C_{12}^{\alpha\beta}} \right\rangle\right] \\
&= \beta \sum_{i=1}^{N_{frames}} \left[\omega^i \log\left(\frac{\omega^i}{\omega_0^i}\right)\frac{\partial \Delta U_i}{\partial C_{12}^{\alpha\beta}} - \omega^i \log\left(\frac{\omega^i}{\omega_0^i}\right)\left\langle \frac{\partial \Delta U}{\partial C_{12}^{\alpha\beta}} \right\rangle\right] \\
&= \beta \left[\left\langle \log\left(\frac{\omega}{\omega_0}\right)\frac{\partial \Delta U}{\partial C_{12}^{\alpha\beta}} \right\rangle - \left\langle \log\left(\frac{\omega}{\omega_0}\right)\right\rangle \left\langle \frac{\partial \Delta U}{\partial C_{12}^{\alpha\beta}} \right\rangle\right] \qquad \text{(S3.10)}
\end{aligned}
$$

where the simplification leading from the second to the third expression follows from the fact that the mean of a mean-subtracted quantity is zero and the expression for $\partial\omega^i/\partial C_{12}$ is recycled from Equation S3.7.

- **$(\partial\Delta U/\partial C_{12})$ and the Lennard Jones combination rules:**

Derivatives of the form: '$\partial\Delta U/\partial C_{12}$', appear both in Equation S3.8 and S3.10. Although ΔU is linear in the $C_{6/12}$ parameters (Equation S3.3), the explicit form of these derivatives depends on whether the Lennard–Jones combination rules (e.g., Lorentz–Berthelot[46]) are enforced. Maintaining or relaxing these rules changes both the number of independent parameters and the complexity of the resulting derivatives.

Combination rules are commonly employed in force fields to extend their transferability beyond the systems used for parameterization, by estimating cross LJ parameters ($\epsilon_{\alpha\beta}$ and $\sigma_{\alpha\beta}$) from the corresponding self-terms ($\epsilon_{\alpha\alpha/\beta\beta}$ and $\sigma_{\alpha\alpha/\beta\beta}$). A widely used choice is the Lorentz–Berthelot rule which is what we enforce in our optimization procedure:

$$
\epsilon_{\alpha\beta} = \sqrt{\epsilon_{\alpha\alpha}\epsilon_{\beta\beta}} \quad \text{and} \quad \sigma_{\alpha\beta} = \frac{\sigma_{\alpha\alpha} + \sigma_{\beta\beta}}{2} \qquad \text{(S3.11)}
$$

Enforcing these combining rules for $\alpha, \beta \in [Li^+, Cl^-, O]$ leads to a 4-dimensional parameter space in the $C_{6/12}$ of the pairs Li-Li and Cl-Cl (self-terms only). However, the derivatives now include energy changes from cross terms. The corresponding Lorentz-Berthelot rules in $C_{6/12}$ may be obtained by inverting the relation between LJ parameters giving the self ϵ and σ as a function of the self $C_{6/12}$:

$$
\epsilon_{\alpha\alpha} = \frac{(C_6^{\alpha\alpha})^2}{4C_{12}^{\alpha\alpha}} \quad \text{and} \quad \sigma_{\alpha\alpha} = \left(\frac{C_{12}^{\alpha\alpha}}{C_6^{\alpha\alpha}}\right)^{1/6} \qquad \text{(S3.12)}
$$

Using these in the Lorentz-Berthelot equations (Equation S3.11) yields the cross-terms:

$$C_6^{\alpha\beta} = 4\epsilon_{\alpha\beta}(\sigma_{\alpha\beta})^6 = \frac{C_6^{\alpha\alpha}C_6^{\beta\beta}}{2^6\sqrt{C_{12}^{\alpha\alpha}C_{12}^{\beta\beta}}}\left[\left(\frac{C_{12}^{\alpha\alpha}}{C_6^{\alpha\alpha}}\right)^{1/6} + \left(\frac{C_{12}^{\beta\beta}}{C_6^{\beta\beta}}\right)^{1/6}\right]^6$$

$$= \frac{1}{2^6\sqrt{C_{12}^{\alpha\alpha}C_{12}^{\beta\beta}}}\left[\left(C_{12}^{\alpha\alpha}C_6^{\beta\beta}\right)^{1/6} + \left(C_{12}^{\beta\beta}C_6^{\alpha\alpha}\right)^{1/6}\right]^6 \quad \text{(S3.13)}$$

$$\text{and} \quad C_{12}^{\alpha\beta} = 4\epsilon_{\alpha\beta}(\sigma_{\alpha\beta})^{12} = \frac{C_6^{\alpha\alpha}C_6^{\beta\beta}}{2^{12}\sqrt{C_{12}^{\alpha\alpha}C_{12}^{\beta\beta}}}\left[\left(\frac{C_{12}^{\alpha\alpha}}{C_6^{\alpha\alpha}}\right)^{1/6} + \left(\frac{C_{12}^{\beta\beta}}{C_6^{\beta\beta}}\right)^{1/6}\right]^{12}$$

$$= \frac{1}{2^{12}(C_6^{\alpha\alpha}C_6^{\beta\beta})\sqrt{C_{12}^{\alpha\alpha}C_{12}^{\beta\beta}}}\left[\left(C_{12}^{\alpha\alpha}C_6^{\beta\beta}\right)^{1/6} + \left(C_{12}^{\beta\beta}C_6^{\alpha\alpha}\right)^{1/6}\right]^{12} \quad \text{(S3.14)}$$

Note that $C_{12}^{\alpha\beta}$ and $C_6^{\alpha\beta}$ each depend on the self-interaction parameters of both species, α and β : $C_{6/12}^{\alpha\alpha}$ and $C_{6/12}^{\beta\beta}$. Hence changing (say) C_{12}^{LiLi} also changes $C_{6/12}^{LiCl}$ and $C_{6/12}^{LiO}$. The energy derivative for a frame i then gets:

$$\frac{\partial \Delta U_i}{\partial C_{12}^{LiLi}} = \zeta_{12}^{LiLi,i} + \left(\frac{\partial C_{12}^{LiCl}}{\partial C_{12}^{LiLi}}\right)\zeta_{12}^{LiCl,i} - \left(\frac{\partial C_6^{LiCl}}{\partial C_{12}^{LiLi}}\right)\zeta_6^{LiCl,i} + \left(\frac{\partial C_{12}^{LiO}}{\partial C_{12}^{LiLi}}\right)\zeta_{12}^{LiO,i} - \left(\frac{\partial C_6^{LiO}}{\partial C_{12}^{LiLi}}\right)\zeta_6^{LiO,i} \quad \text{(S3.15)}$$

The final ingredients are the derivatives of the cross $C_{6/12}^{\alpha\beta}$ with respect to the self ones. Defining an auxiliary variable $\tau = \left(C_{12}^{\alpha\alpha}C_6^{\beta\beta}\right)^{1/6} + \left(C_{12}^{\beta\beta}C_6^{\alpha\alpha}\right)^{1/6}$ and its derivatives:

$$\frac{\partial \tau}{\partial C_6^{\alpha\alpha}} = \frac{C_{12}^{\beta\beta}}{6\left(C_6^{\alpha\alpha}C_{12}^{\beta\beta}\right)^{5/6}} \quad \text{and} \quad \frac{\partial \tau}{\partial C_{12}^{\alpha\alpha}} = \frac{C_6^{\beta\beta}}{6\left(C_{12}^{\alpha\alpha}C_6^{\beta\beta}\right)^{5/6}} \quad \text{(S3.16)}$$

simplifies the proceedings:

$$
\begin{aligned}
\frac{\partial C_{12}^{\alpha\beta}}{\partial C_{12}^{\alpha\alpha}} &= \frac{1}{2^{12}\left(C_6^{\alpha\alpha}C_6^{\beta\beta}\right)\sqrt{C_{12}^{\beta\beta}}}\frac{\partial}{\partial C_{12}^{\alpha\alpha}}\left[\frac{\tau^{12}}{\sqrt{C_{12}^{\alpha\alpha}}}\right] \\
&= \frac{1}{2^{12}\left(C_6^{\alpha\alpha}C_6^{\beta\beta}\right)\sqrt{C_{12}^{\beta\beta}}}\left[-\frac{\tau^{12}}{2(C_{12}^{\alpha\alpha})^{3/2}} + \frac{2C_6^{\beta\beta}\tau^{11}}{\sqrt{C_{12}^{\alpha\alpha}}(C_{12}^{\alpha\alpha}C_6^{\beta\beta})^{5/6}}\right] \\
&= C_{12}^{\alpha\beta}\left[-\frac{1}{2C_{12}^{\alpha\alpha}} + \frac{2C_6^{\beta\beta}}{\tau(C_{12}^{\alpha\alpha}C_6^{\beta\beta})^{5/6}}\right] \\
&= \frac{C_{12}^{\alpha\beta}}{C_{12}^{\alpha\alpha}}\left[-\frac{1}{2} + \frac{2(C_{12}^{\alpha\alpha}C_6^{\beta\beta})^{1/6}}{\tau}\right]
\end{aligned}
\tag{S3.17}
$$

Similarly, one may obtain the remaining derivatives as:

$$
\begin{aligned}
\frac{\partial C_6^{\alpha\beta}}{\partial C_6^{\alpha\alpha}} &= \frac{C_6^{\alpha\beta}}{C_6^{\alpha\alpha}}\left[\frac{(C_{12}^{\beta\beta}C_6^{\alpha\alpha})^{1/6}}{\tau}\right] \\
\frac{\partial C_6^{\alpha\beta}}{\partial C_{12}^{\alpha\alpha}} &= \frac{C_6^{\alpha\beta}}{C_{12}^{\alpha\alpha}}\left[-\frac{1}{2} + \frac{(C_{12}^{\alpha\alpha}C_6^{\beta\beta})^{1/6}}{\tau}\right] \\
\frac{\partial C_6^{\alpha\beta}}{\partial C_{12}^{\alpha\alpha}} &= \frac{C_6^{\alpha\beta}}{C_{12}^{\alpha\alpha}}\left[-1 + \frac{2(C_{12}^{\beta\beta}C_6^{\alpha\alpha})^{1/6}}{\tau}\right]
\end{aligned}
\tag{S3.18}
$$

Replacing the appropriate terms in Equation S3.15 gives the required expressions for $\partial\Delta U/\partial C_{12}$.

S3.1.D Workflow

Access to analytical expressions for both the loss function and its gradient enables the use of efficient gradient-based optimization methods. In this work, optimization was performed using the limited-memory BFGS (L-BFGS) algorithm as implemented in SciPy. To maintain simplicity and probe the minimal parameter space required to reproduce the experimental density trends, the Lorentz–Berthelot combination rules were retained, restricting optimization to the self-interaction parameters, $\epsilon_{\alpha\alpha}$ and $\sigma_{\alpha\alpha}$ (and hence $C_{6/12}$), of Li^+ and Cl^-, although recent force fields such as **M₁₉** have chosen to relax these constraints. The optimization followed an iterative scheme consisting of alternating cycles of running loss minimization (Equation. S3.4) and new NPT simulations.

As discussed earlier, the optimization behavior depends critically on the hyperparameter θ. Having it set to 0 permits larger steps toward reducing χ^2, but excessive deviation from the initial force field compromises the reliability of the reweighted averages, as many configurations acquire negligible weights ($\omega^i \sim 0$). Conversely, choosing θ close to 1 imposes a strong penalty on deviations from the reference force field, effectively preventing

meaningful improvement in χ^2. To this end, Kish effective sample size was used to iteratively get an estimate of an optimal θ given the input FF and trajectories. The Kish effective sample size, or simply Kish Size (KS), estimates the number of independent samples that would provide the same statistical precision as the weighted ensemble[45]. Given the Boltzmann weights from changing the parameters, ω^i, KS is defined as:

$$KS = \frac{(\sum_i \omega^i)^2}{\sum_i (\omega^i)^2} \tag{S3.19}$$

Having all $\omega^i = 1/N_{frames}$, the KS naturally becomes equal to N_{frames}. Thus, it effectively represents the fraction of the total sample that meaningfully contributes to the ensemble average as the ω^i values vary. In this work, θ was chosen as the smallest value that yielded a Kish size of at least 50% of the total number of input frames. A new set of simulations was then performed using the force field optimized with this θ, and the process was iterated using the resulting trajectory as the new starting point. The overall procedure is summarized in the flowchart below (Figure S3.1) that combines all the various steps elucidated earlier.

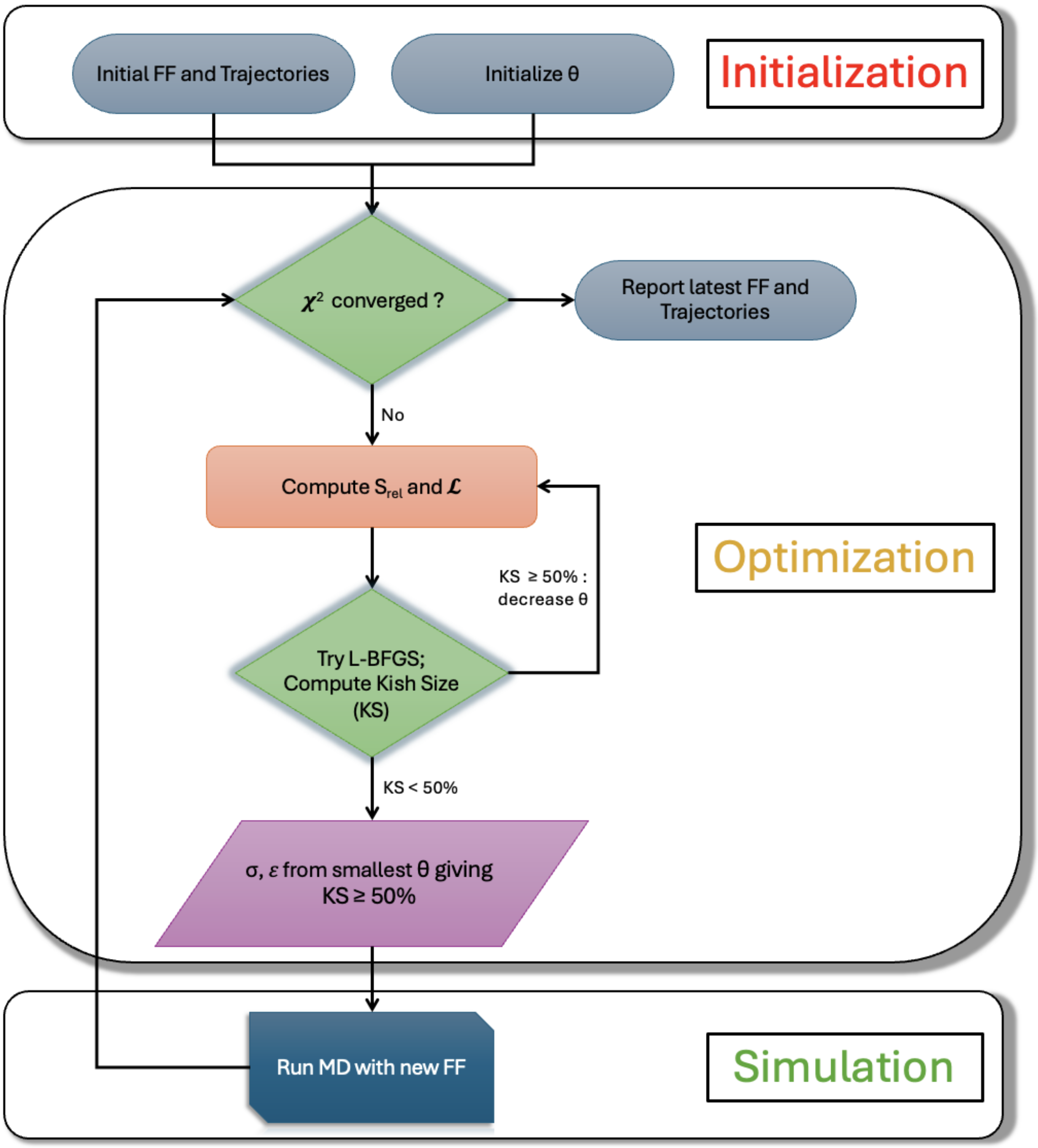

Figure S3.1: *Flowchart of the FF optimization protocol summarizing the key steps*

S3.2 Force Fields

The parameters for the **JC** and the charge scaled $\mathbf{M_{19}}$ force fields are summarized below, together with the final parameter sets obtained from separate optimizations of each. The resulting optimized models are referred to as $\mathbf{JC_{opt}}$ and $\mathbf{M_{19}^{opt}}$ respectively. An important distinction between $\mathbf{M_{19}}$ and $\mathbf{M_{19}^{opt}}$ is that the former breaks the LB combination rules and is characterized by separate ion-counter ion and ion-water parameters (which are available in[39]), while $\mathbf{M_{19}^{opt}}$ enforces them.

Table S3.1: Force-field parameters

**Parameters**	**JC**		$\mathbf{JC_{opt}}$		$\mathbf{M_{19}}$		$\mathbf{M_{19}^{opt}}$	
	Li^+	Cl^-	Li^+	Cl^-	Li^+	Cl^-	Li^+	Cl^-
q (e)	1.00	-1.00	1.00	-1.00	0.85	-0.85	0.85	-0.85
ε (kJ/mol)	0.435	0.049	0.374	0.059	0.435	0.077	3.534	0.244
σ (nm)	0.144	0.492	0.117	0.482	0.144	0.470	0.102	0.417

S3.3 MD Simulation

All simulations have been performed using GROMACS 2023.3[47]. Two independent sets of trajectories are involved: one for force field optimization and another for statistical analysis. Each system consisted of aqueous LiCl contained in cubic boxes of initial side-length 4.0 nm corresponding to initial concentrations of 0 M, 0.5 M, 1 M, 2 M, 3 M, 4 M, 5 M, 6 M, 7 M, 8 M, and 9 M. All systems were subject to energy minimization followed by 200 ps of NVT equilibration at 300 K. In addition, for the simulations involving the optimization of the force-field parameters, 12 ns of NPT simulations were performed at 300 K and 1 atm, with the first 2 ns treated as equilibration. For the statistical analysis conducted in our simulations including determining the density and all the structural properties like the radial distribution functions as well as the graph-analysis examining clusters of ions, 1 μs NPT simulations were carried out under the same conditions. The first 100 ns were discarded for equilibration, and all analyses were performed on the remaining 900 ns of trajectory. Simulations were performed for the original JC and $\mathbf{M_{19}}$ force-fields, as well as their corresponding optimized variants, $\mathbf{JC_{opt}}$ and $\mathbf{M_{19}^{opt}}$. TIP4P/2005 water was used throughout.

The equations of motion were integrated using the leap-frog algorithm[48] with a 2 fs timestep. Temperature and pressure were maintained using the stochastic velocity-rescaling thermostat[49] (τ_t = 0.1 ps) and isotropic stochastic cell-rescaling barostat[50] (τ_p = 0.5 ps), respectively. Both long-range electrostatic and van-der-Waals interactions were treated with the Particle Mesh Ewald (PME) method[51], and LINCS[52] constraints were applied to preserve the rigid water geometry.

During the NPT simulations, the box volumes fluctuated and equilibrated to values different from the initial ones. Therefore, the molar concentrations were recalculated a posteriori using the time-averaged volumes from the trajectories. This may be avoided by the use of molality (m) as the concentration scale, since it varies with the mass of solvent. Details of the systems are tabulated below.

Table S3.2: Simulated Systems

Force Field	Initial Salt Conc. (M)	Avg. Salt Conc. (M)	Salt Conc. (m)	N_{H_2O}	N_{ions} (Li^+ + Cl^-)	$\langle \rho_{sim}^{conc} \rangle$ (g/mL)	V_m^{LiCl} (mL/mol)	$V_m^{H_2O}$ (mL/mol)
**TIP4P/2005**	0	0.0000	0.0000	2211	0	0.9962 ± 0.0001	—	18.0779 ± 0.0014
**TIP4P/2005 + JC**	0.5	0.4790	0.4850	2173	38	1.0071 ± 0.0001	22.1933 ± 0.0655	18.0704 ± 0.0016
	1	0.9900	1.0150	2133	78	1.0169 ± 0.0001	22.6962 ± 0.0516	18.0636 ± 0.0015
	2	1.9800	2.0780	2057	154	1.0367 ± 0.0001	23.6173 ± 0.0296	18.0381 ± 0.0013
	3	3.0160	3.2540	1979	232	1.0549 ± 0.0001	24.5002 ± 0.0188	17.9960 ± 0.0017
	4	4.0510	4.4920	1903	308	1.0728 ± 0.0001	25.2704 ± 0.0233	17.9426 ± 0.0025
	5	5.1280	5.8700	1825	386	1.0905 ± 0.0001	26.5501 ± 0.0279	17.8329 ± 0.0033
	6	6.2000	7.3310	1749	462	1.1078 ± 0.0001	26.3598 ± 0.0282	17.8308 ± 0.0035
	7	7.3240	8.9690	1671	540	1.1255 ± 0.0001	26.5011 ± 0.0230	17.8110 ± 0.0037
	8	8.4390	10.7190	1595	616	1.1436 ± 0.0001	26.1975 ± 0.0435	17.8662 ± 0.0086
	9	9.6040	12.6970	1517	694	1.1619 ± 0.0001	25.2230 ± 0.1050	18.0738 ± 0.0221
**TIP4P/2005 + JC_{opt}**	0.5	0.4800	0.4850	2173	38	1.0084 ± 0.0001	18.4724 ± 0.0556	18.0759 ± 0.0014
	1	0.9930	1.0150	2133	78	1.0205 ± 0.0001	18.9591 ± 0.0436	18.0693 ± 0.0013
	2	1.9930	2.0780	2057	154	1.0435 ± 0.0001	19.8315 ± 0.0247	18.0452 ± 0.0011
	3	3.0500	3.2540	1979	232	1.0669 ± 0.0001	20.6413 ± 0.0152	18.0066 ± 0.0014
	4	4.1110	4.4920	1903	308	1.0893 ± 0.0001	21.3213 ± 0.0186	17.9595 ± 0.0020
	5	5.2300	5.8700	1825	386	1.1124 ± 0.0001	21.8682 ± 0.0230	17.9089 ± 0.0026
	6	6.3500	7.3310	1749	462	1.1353 ± 0.0001	22.1975 ± 0.0221	17.8703 ± 0.0027
	7	7.5310	8.9690	1671	540	1.1587 ± 0.0001	22.2420 ± 0.0183	17.8647 ± 0.0029
	8	8.7190	10.7190	1595	616	1.1829 ± 0.0001	21.8829 ± 0.0345	17.9295 ± 0.0068

Force Field	Initial Salt	Avg. Salt	Salt Conc.	N_{H_2O}	N_{ions}	$\langle\rho_{sim}^{conc}\rangle$	V_m^{LiCl}	$V_m^{H_2O}$
	9	9.9810	12.6970	1517	694	1.2090 ± 0.0001	20.9340 ± 0.0812	18.1314 ± 0.0170
**TIP4P/2005 + M_{19}**	0.5	0.5060	0.4850	2173	38	1.0070 ± 0.0001	20.3828 ± 0.0681	18.0823 ± 0.0014
	1	1.0210	1.0150	2133	78	1.0180 ± 0.0001	20.4048 ± 0.0558	18.0822 ± 0.0014
	2	2.0910	2.0780	2057	154	1.0400 ± 0.0001	20.4246 ± 0.0438	18.0820 ± 0.0013
	3	3.1800	3.2540	1979	232	1.0630 ± 0.0001	20.4489 ± 0.0249	18.0813 ± 0.0011
	4	4.3240	4.4920	1903	308	1.0870 ± 0.0001	20.4473 ± 0.0156	18.0815 ± 0.0014
	5	5.4890	5.8700	1825	386	1.1120 ± 0.0001	20.4071 ± 0.0191	18.0844 ± 0.0021
	6	6.6990	7.3310	1749	462	1.1370 ± 0.0001	20.3084 ± 0.0233	18.0937 ± 0.0027
	7	7.9740	8.9690	1671	540	1.1640 ± 0.0001	20.1328 ± 0.0220	18.1147 ± 0.0027
	8	9.2820	10.7190	1595	616	1.1920 ± 0.0001	19.8380 ± 0.0178	18.1583 ± 0.0029
	9	10.6730	12.6970	1517	694	1.2230 ± 0.0001	18.7215 ± 0.0802	18.3799 ± 0.0168
**TIP4P/2005 + M_{19}^{opt}**	0.5	0.4790	0.4850	2173	38	1.0077 ± 0.0001	19.1870 ± 0.0557	18.0780 ± 0.0014
	1	0.9920	1.0150	2133	78	1.0198 ± 0.0001	19.5048 ± 0.0437	18.0738 ± 0.0013
	2	1.9900	2.0780	2057	154	1.0424 ± 0.0001	20.0777 ± 0.0247	18.0579 ± 0.0011
	3	3.0460	3.2540	1979	232	1.0655 ± 0.0001	20.6122 ± 0.0154	18.0325 ± 0.0014
	4	4.1060	4.4920	1903	308	1.0882 ± 0.0001	21.0615 ± 0.0190	18.0013 ± 0.0021
	5	5.2260	5.8700	1825	386	1.1118 ± 0.0001	21.4204 ± 0.0234	17.9681 ± 0.0027
	6	6.3490	7.3310	1749	462	1.1351 ± 0.0001	21.6302 ± 0.0223	17.9436 ± 0.0028
	7	7.5380	8.9690	1671	540	1.1599 ± 0.0001	21.6420 ± 0.0178	17.9425 ± 0.0029
	8	8.7330	10.7190	1595	616	1.1850 ± 0.0001	21.3735 ± 0.0336	17.9909 ± 0.0067
	9	10.0010	12.6970	1517	694	1.2116 ± 0.0001	20.6922 ± 0.0802	18.1358 ± 0.0169

As shown in Table S3.2 above, the final molarities can change for systems simulated with different force fields. When comparing corresponding systems across FF, concentrations are hence expressed in terms of molality for simplicity.

Uncertainties in the simulated densities were estimated by non-parametric bootstrap resampling of the per-frame density (10^4 resamples) at each LiCl molality. 3000 frames were extracted from the remaining 900 ns of each concentration trajectory, with a separation of 300 ps between consecutive frames. This spacing is significantly larger than the largest density correlation time estimated to be ~13 ps at the highest concentrations, ensuring statistical independence of the sampled configurations. Third-order polynomials (Equation S1.1) were independently fitted to each of the 10^4 resampled density sets, and ρ and $\left(\frac{\partial\rho}{\partial m}\right)$ obtained from each fit were used to compute V_m^{LiCl} and $V_m^{H_2O}$ at each molality. The reported PMVs and their uncertainties correspond to the mean and standard deviation across bootstrap replicates. The reported densities and associated uncertainties are given by the bootstrap mean and standard error, independent of the polynomial fitting procedure.
As a cross-check, a single third-order polynomial was fitted to the mean bootstrap density using weighted least squares, with weights given by the bootstrap standard error. The resulting coefficient covariance was propagated to the PMV uncertainties using standard first-order error propagation[53],

$$\sigma_{\bar{V}}^2 = \left(\frac{\partial\bar{V}}{\partial\rho}\right)^2 \sigma_\rho^2 + \left(\frac{\partial\bar{V}}{\partial(\partial\rho/\partial m)}\right)^2 \sigma_{(\partial\rho/\partial m)}^2 + 2\left(\frac{\partial\bar{V}}{\partial\rho}\right)\left(\frac{\partial\bar{V}}{\partial(\partial\rho/\partial m)}\right)\mathrm{Cov}(\rho, (\partial\rho/\partial m)) \tag{S3.20}$$

where $\partial\bar{V}/\partial\rho$ and $\partial\bar{V}/\partial(\partial\rho/\partial m)$ were obtained analytically from the polynomial expressions in Equation S1.13. The uncertainties σ_ρ, $\sigma_{(\partial\rho/\partial m)}$, and $\mathrm{Cov}(\rho, (\partial\rho/\partial m))$ were obtained from the covariance matrix of the fitted polynomial coefficients using the corresponding linear error propagation relations. The two approaches were found to agree within ~1% for both partial molar volumes across the full concentration range.

S3.4 Comparison of Force Fields

The following section presents a comparative analysis of the force fields presented in the main text against other models in the literature. The ensuing analyses utilized NumPy, scikit-learn, SciPy, MDAnalysis and GROMACS 2023.3.

S3.4.A Density and PMV

The density and PMV trends obtained from the **JC**, $\mathbf{M_{19}}$, $\mathbf{JC_{opt}}$ and $\mathbf{M_{19}^{opt}}$ parameter sets are compared in Figure S3.2. While the density profile of **JC** shows clear deviations from the present experimental data, $\mathbf{M_{19}}$ performs considerably better - consistent with the fact that density information from earlier studies was incorporated into its parameterization. However, the PMV trends predicted by both models are still relatively inaccurate. This discrepancy likely arises from the large coefficient associated with the density derivative in the PMV expressions (Equations S1.9 and S1.12), which amplifies even small deviations in the underlying density. The RMSE comparison for the different models is shown in Table S3.3.

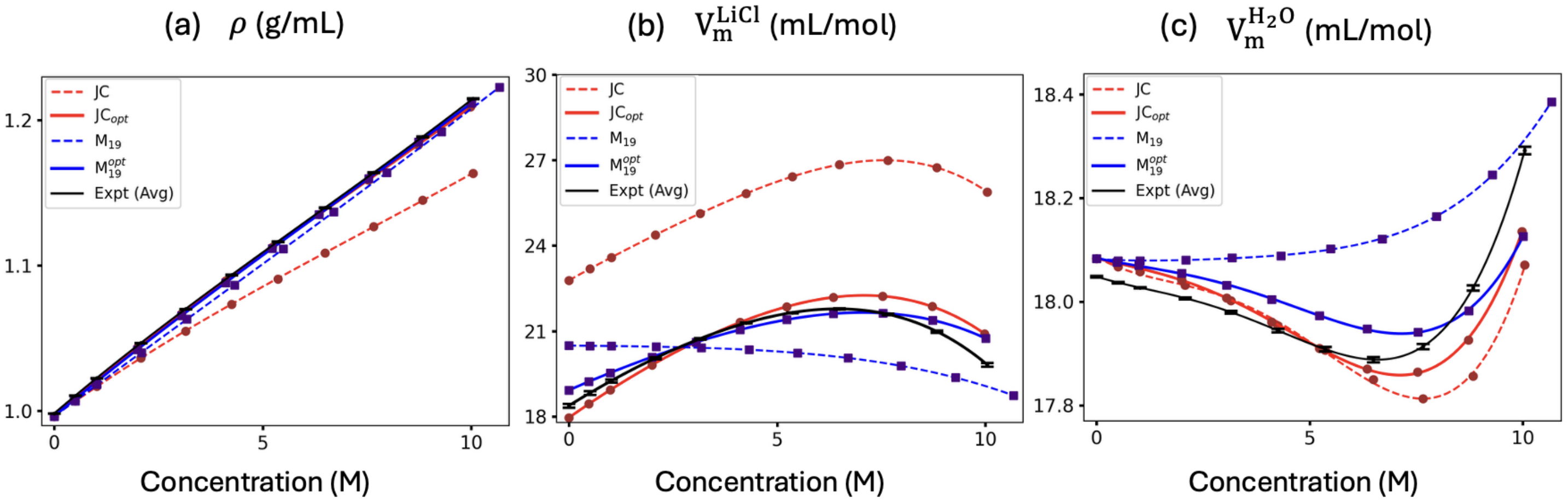

Figure S3.2: *Comparison of force-field performance across concentration for (a) solution density, (b) LiCl PMV, and (c) water PMV. LiCl PMV at 0 M concentration are obtained via extrapolation*

Table S3.3: RMSE comparison between simulation and experimental trends across force fields

Set	JC	JC_{opt}	M_{19}	M_{19}^{opt}
RMSE ρ (g/mL)	0.028	0.003	0.004	0.002
RMSE V_m^{LiCl} (mL/mol)	4.309	0.508	1.045	0.330
RMSE $V_m^{H_2O}$ (mL/mol)	0.066	0.057	0.130	0.062

S3.4.B RDF and Coordination Numbers

Radial distribution functions (RDFs) for the pairs: Li^+-Li^+, Cl^--Cl^-, Li^+-Cl^-, Li^+-O, Cl^--O and O-O are shown for **JC** and $\mathbf{JC_{opt}}$ force fields, with the corresponding running coordination numbers (CN) displayed as insets. For comparison across force fields, corresponding systems are labeled by their concentration in molality. In addition, the LiCl RDF for the $\mathbf{M_{19}}$ and $\mathbf{M_{19}^{opt}}$ models are presented to compare the degree of ion pairing in each model.

- **Li^+-Cl^-**:

  A stark contrast is observed in the Li+-Cl^- pairing behavior of **JC** and $\mathbf{M_{19}}$ (Figure S3.3 a and c respectively). The concentration-dependent, compact ion-pair peak at 0.23 nm is strongly pronounced in **JC**, yielding a coordination number of 0.8 at 0.3 nm for 12.69 m, whereas it is essentially absent in $\mathbf{M_{19}}$ (CN ~ 0 at the same concentration). This may be attributed partly to the reduced charges of the ions and the large Cl^- σ, 0.47 nm, which is reduced to 0.42 nm upon refinement in $\mathbf{M_{19}^{opt}}$ (Table S3.1). The weak ion association in M19 is consistent with previous studies[54,55]. By comparison, both the optimized FF - $\mathbf{JC_{opt}}$ and $\mathbf{M_{19}^{opt}}$ (Figure S3.3 b and d respectively) exhibit intermediate behavior, with coordination numbers of 0.41 at the same distance and concentration - falling between the two extremes defined by **JC** and $\mathbf{M_{19}}$. All force fields exhibit an additional peak at 0.44 nm, corresponding to the second layer of counter ions (which serves as the first layer for $\mathbf{M_{19}}$).

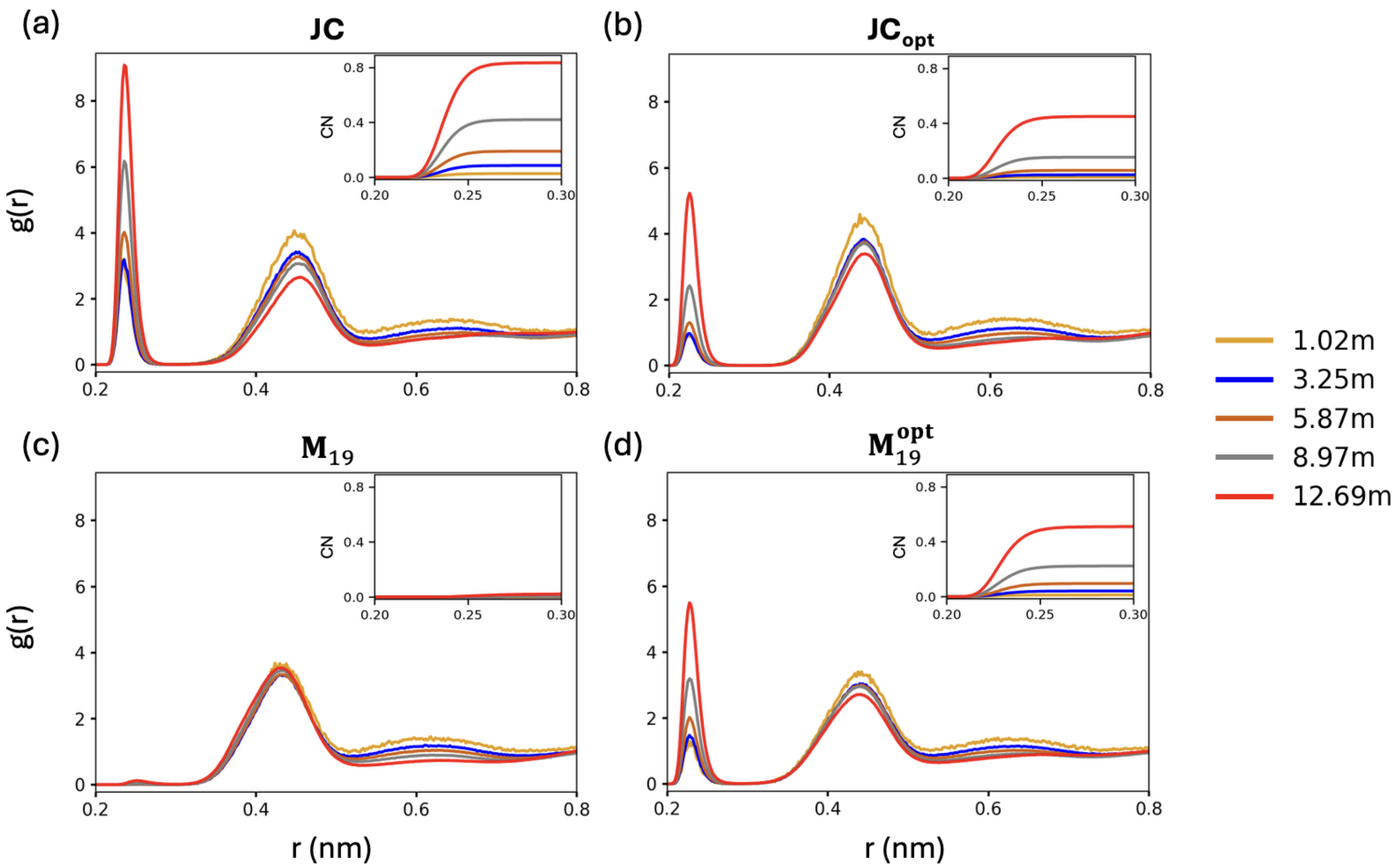

Figure S3.3: *Comparison of the Li^+-Cl^- RDF for the (a)* ***JC****, (b)* ***JC$_{opt}$****, (c)* ***M$_{19}$*** *and (d)* ***M$_{19}$*** *FF sets. The insets show the running Coordination Number for the same up to the first minimum distance of the RDF.*

The similarity in the Li^+-Cl^- trends for the optimized force fields suggests that the degree of ion pairing is closely linked to the PMV behavior that these force fields were tuned to reproduce. This consistency also supports the accuracy of the underlying structural correlations - since PMV reflects the ion–ion interactions governed by distance-dependent potentials, its correct reproduction indicates that the resulting RDF is well captured, irrespective of differences in parametrization such as the partial charges.

- **Li^+-O:**

The Li^+-O RDF and CN trends are complementary to the Li^+-Cl^-. For **JC** (Figure S3.4 a), strong ion pairing leads to a steady decrease in the first-shell Li^+-O coordination (0.19 nm), dropping from 3.8 at 1.02 m to 3.0 at 12.69 m. In contrast, **JC$_{opt}$** (Figure S3.4 b) retains higher coordination (3.4) at the same concentration. A second shell peak may be seen in all RDFs at 0.41 nm.

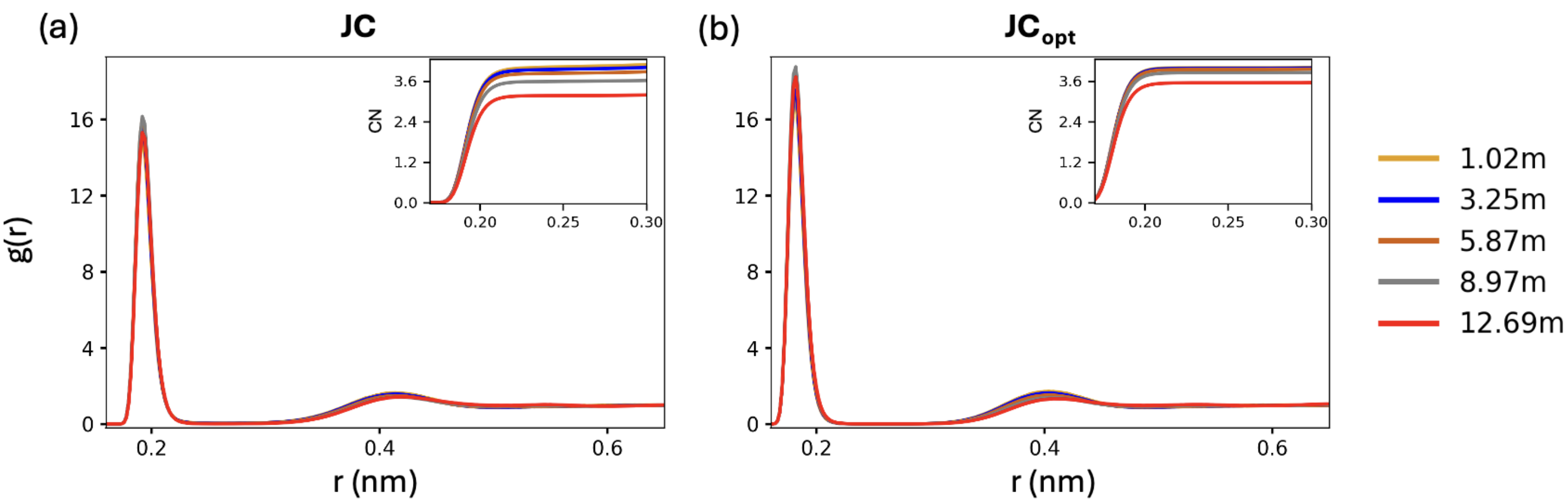

Figure S3.4: *Comparison of the Li^+-O RDF for the (a)* ***JC*** *and (b)* ***JC_{opt}*** *FF sets. The insets show the running Coordination Number for the same up to the first minimum distance of the RDF.*

- **Li^+-Li^+:**

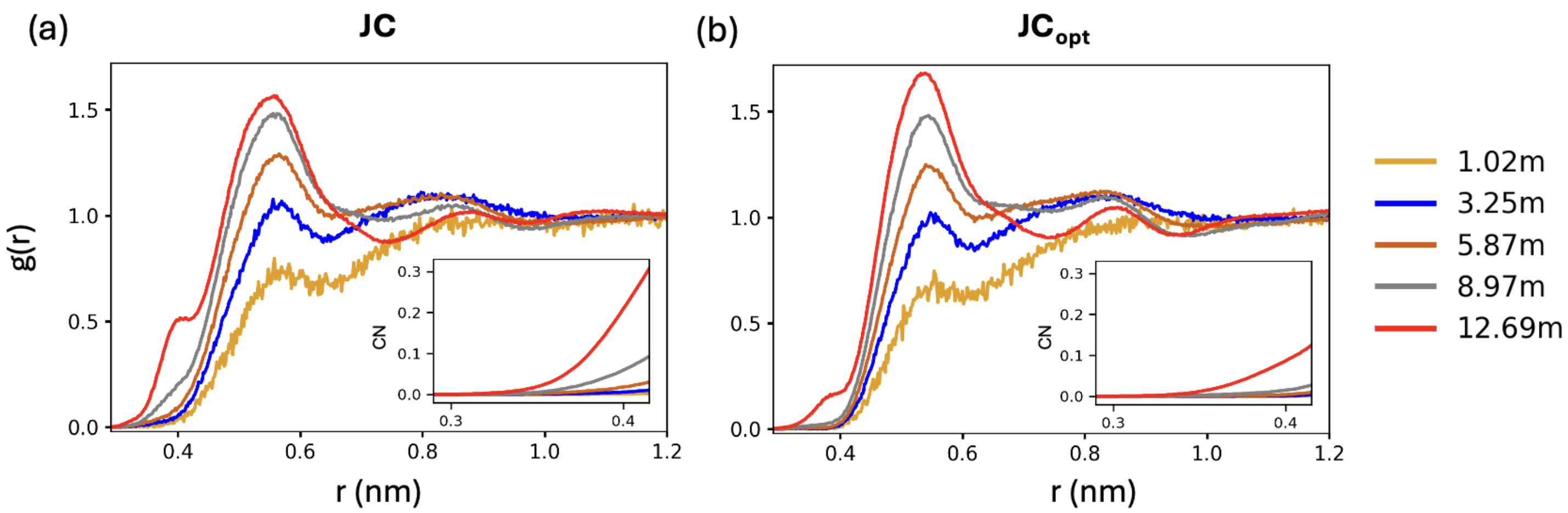

Figure S3.5: *Comparison of the Li^+-Li^+ RDF for the (a)* ***JC*** *and (b)* ***JC_{opt}*** *FF sets. The insets show the running Coordination Number for the same up to the first minimum distance of the RDF.*

The most prominent feature in all Li^+-Li^+ RDFs is the primary peak at 0.56 nm. The height of this peak increases systematically with concentration, reflecting the enhanced likelihood of Li^+-Li^+ encounters in more crowded solutions. In addition, both force fields exhibit the emergence of a secondary feature at distances $<$ 0.4 nm at high concentrations. This short-range signal indicates a growing propensity for like-ion proximity, enabled by counter-ion–bridged structures that temporarily mitigate direct Coulombic repulsion. This is more pronounced in **JC** (Figure S3.5 a). In contrast, the LJ optimization applied in **JC_{opt}** (Figure S3.5 b) appears to suppress the magnitude of this short-range feature, indicating reduced stabilization of these bridged, contact-like arrangements.

- **Cl^--Cl^-:**

The Cl^--Cl^- RDF and CN exhibit trends similar to those of Li^+-Li^+. Across both force fields, the two main concentration-dependent peaks appear at 0.49 and 0.75 nm. As with the short-range feature in the Li^+-Li^+, both **JC** and **JC_{opt}** (Figure S3.6 a and b respectively) display an additional peak near 0.39 nm, indicating

compact Cl$^-$ aggregation. This effect is strongest in **JC** (CN 0.57 at 0.42 nm at 12.69 m). The optimized FF (**JC$_{opt}$**) again shows diminished ion-pairing behavior, with CN 0.23 at 0.42 nm. Finally, a weak peak develops around 0.62 nm at the highest concentration in both FFs, reflecting the enhanced populations of ion rings and chains.

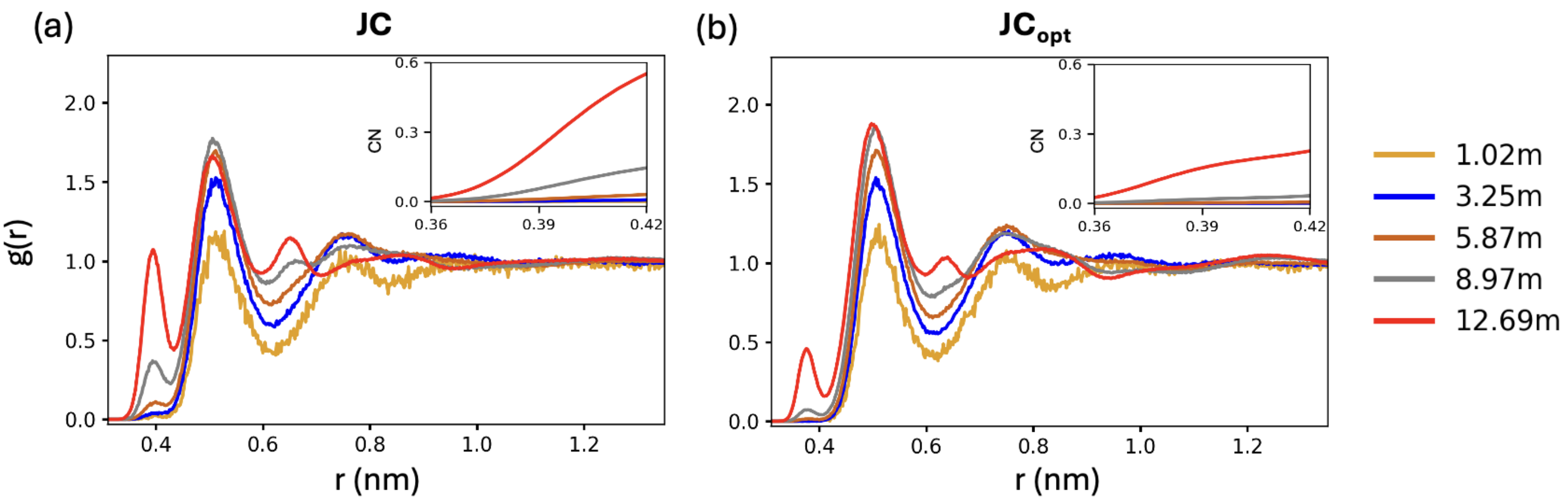

Figure S3.6: *Comparison of the Cl$^-$-Cl$^-$ RDF for the (a)* ***JC*** *and (b)* ***JC$_{opt}$*** *FF sets. The insets show the running Coordination Number for the same up to the first minimum distance of the RDF.*

- **Cl$^-$-O:**

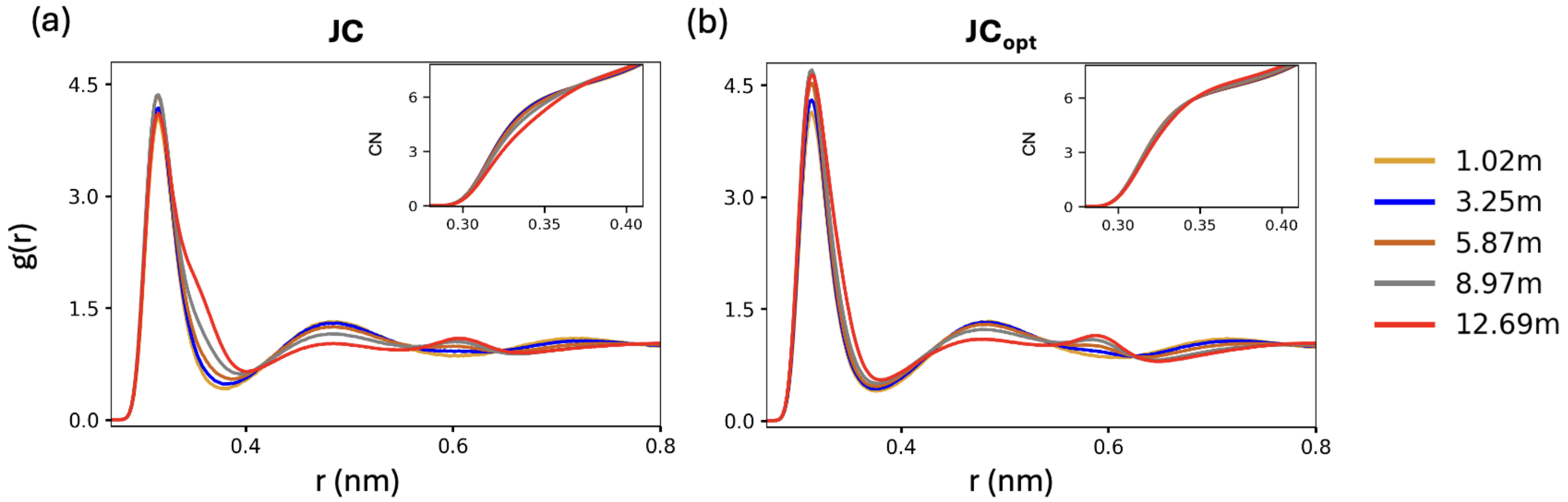

Figure S3.7: *Comparison of the Cl$^-$-O RDF for the (a)* ***JC*** *and (b)* ***JC$_{opt}$*** *FF sets. The insets show the running Coordination Number for the same up to the first minimum distance of the RDF.*

The Cl$^-$-O RDF and CN are similar across both the force fields, showing two major peaks at 0.32 and 0.48 nm, with coordination numbers reaching 7.2 at 0.4 nm. In addition, **JC** (Figure S3.7 a) shows some broadening of the first shell at the highest concentration. Cl$^-$ hydration structure does not differ substantially across force fields.

- **O-O:**

The first (0.29 nm) and second (0.45 nm) peaks for the O–O RDF exhibit broadly similar trends across force fields. The first-shell peak progressively shifts to 0.315 nm at higher LiCl concentrations, reflecting

distortion of the hydrogen-bond network due to intercalation of ion aggregates, consistent with past studies[56]. This distortion initially appears as a broadening and reduction in peak height for both force fields. Beyond this stage, **JC** (Figure S3.8 a) develops a broad feature spanning 0.29–0.32 nm, whereas $\mathbf{JC_{opt}}$ (Figure S3.8 b) forms a more distinct peak at 0.315 nm. The second-shell peak systematically weakens with increasing concentration, indicating the gradual breakdown of the ordered water hydrogen-bond network as the ionic environment becomes more crowded.

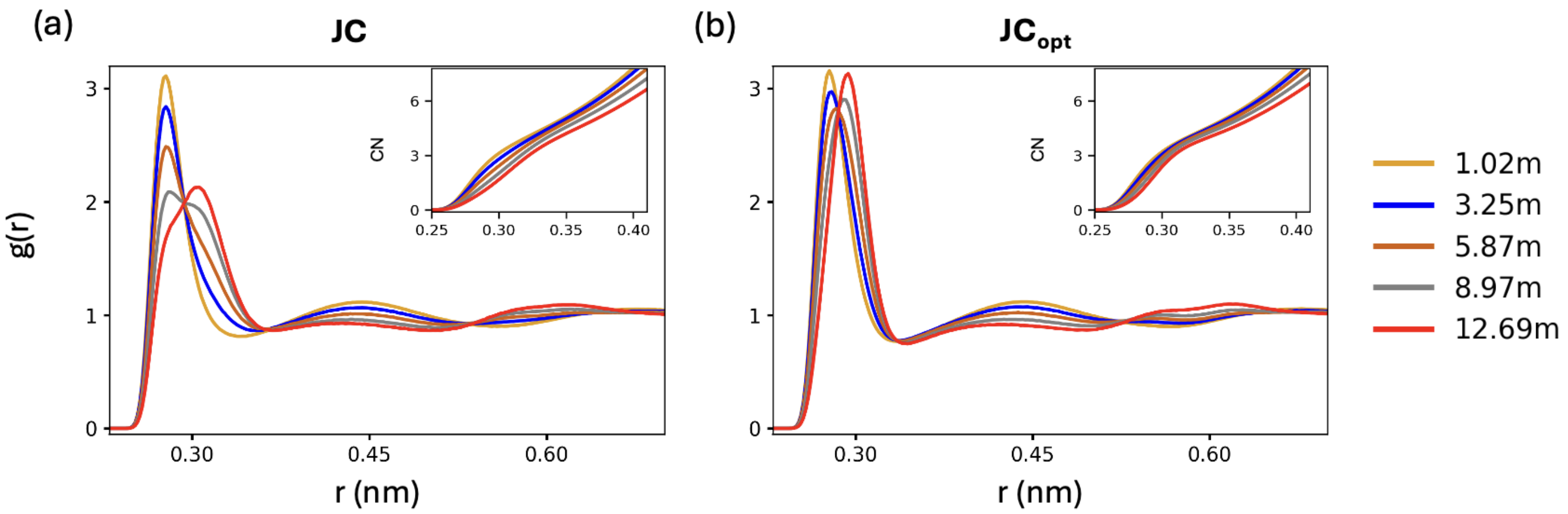

Figure S3.8: *Comparison of the O-O RDF for the (a)* ***JC*** *and (b)* $\mathbf{JC_{opt}}$ *FF sets. The insets show the running Coordination Number for the same up to the first minimum distance of the RDF.*

This concludes the comparative study of results obtained using different force fields. Below, all analyses using classical MD are restricted to simulations performed with the JCopt parameter set.

S3.5 Chemical Graphs

In the present work, we use graphs to quantify changes in the ion-network as a function of concentration. The procedure for their construction, along with the subsequent analyses, is outlined below. Libraries used for this analysis were MDAnalysis and NetworkX.

S3.5.A Construction of Chemical Graphs

In the present work, the protocol described by Choi et. al.[57–59] was adapted to construct geometric chemical graphs. Ions (excluding water molecules) were treated as nodes, and edges were assigned between node pairs that lay within a prescribed cutoff distance for each trajectory frame. Following Choi et al., the cutoff was taken as the position of the first minimum in the corresponding ion-ion RDF (the first-shell boundary) for the pair of interest. Since three ion pairs are possible - Li^+-Li^+, Cl^--Cl^- and Li^+-Cl^- - three cutoffs were required.

To estimate the population of compact ion clusters, the sub-0.4 nm features observed in the Li^+-Li^+ and Cl^--Cl^- RDFs (Figure. S3.5 and Figure. S3.6 respectively) were used as the relevant first-shell distances. For $\mathbf{JC_{opt}}$, these short-range peaks emerge at concentrations around 8.9 m and above - coinciding with the turning points in the PMV trends - and therefore the corresponding distances (r_{cut}) at 12.69 m were selected uniformly for all ion pairs. The resulting cutoffs are listed in table S3.4 below.

Table S3.4: Distance Cutoff for Constructing Graphs

Pair	Li^+-Li^+	Cl^--Cl^-	Li^+-Cl^-
r_{cut} (nm)	0.412	0.410	0.296

The edges generated in each frame are stored in an adjacency matrix $A_{\alpha\beta}$, where the element $a_{\alpha\beta} = 1$, if an edge exists between ions α and β, otherwise, $a_{\alpha\beta} = 0$. Once constructed, connected clusters can be readily identified as contiguous sets of linked nodes. Cluster populations were extracted from 3000 frames sampled uniformly over the final 900 ns of the NPT simulations at each concentration and were then used for further analysis.

S3.5.B Intracluster Statistics

As noted above, clusters of size greater than 1 can be formed from multiple combinations of Li+ and Cl- ions, which can be readily distinguished by their net charge. For clusters of size 2, the three possible combinations: Li^+-Li^+, Cl^--Cl^- and Li^+-Cl^- carry net charges of 2e, -2e and 0 respectively.

Similarly, clusters of size 3 include the combinations: Li^+-Li^+-Li^+, Li^+-Li^+-Cl^-, Li^+-Cl^--Cl^- and Cl^--Cl^--Cl^- with corresponding net charges of +3e, +e, -e, and -3e. The Probability Mass Functions (PMF) of these cluster types, categorized by their net charge, are compared in Figure S3.9 To reduce statistical uncertainty in the PMF calculations, histograms are constructed for a given cluster size only if the total number of occurrences of that cluster across all trajectory frames at a given concentration is at least 100.

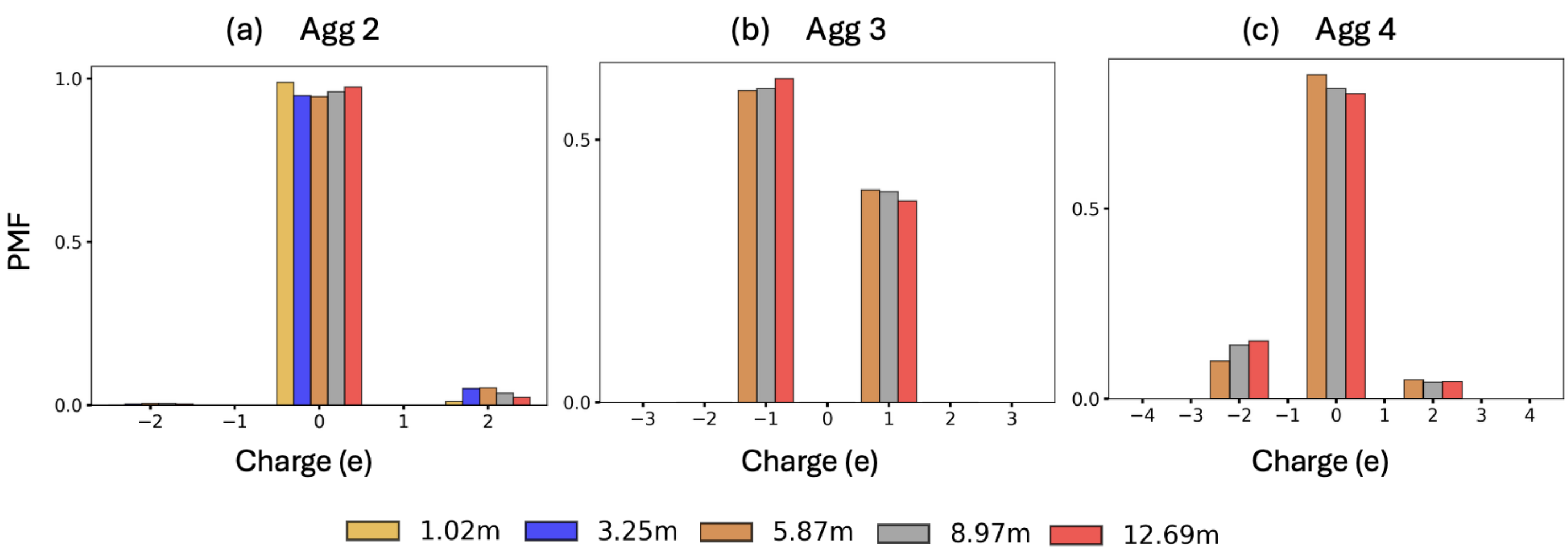

Figure S3.9: *Probability Mass Functions of clusters with different net charges within a given cluster size, representing the various ionic combinations for clusters of size (a) 2, (b) 3 and (c) 4.*

As evident from Figure S3.9, for clusters containing an even number of ions, the combinations comprising equal numbers of cations and anions, forming neutral clusters, are the dominant species. This behavior is expected, as charge neutrality minimizes electrostatic repulsion. More interestingly, for clusters containing an odd number of ions (with size > 1), the combinations featuring one excess Cl^- ion, corresponding to a net charge of -1e, are more populous than the analogous clusters containing one excess Li^-. In the specific case of clusters of size 3 (Figure. S3.9b), clusters with one additional Cl^- are nearly 50% more abundant than those with an extra Li^+.

S3.5.C Topology of Clusters

Beyond the statistical analysis of ionic compositions within clusters of a given size, the topology of the evolving ion networks constitutes an important aspect of electrolyte-water systems. Constructing geometric graphs from the instantaneous ion configurations enables the identification of spatial correlations that extend beyond simple pairwise effects. In the present work, the characterization of chains and rings formed by these connected graphs (clusters) was focused on, providing additional insight into the structural organization of ions in solution.

In this context, a chain is defined as a contiguous set of connected ions with two (linear chain) or more (branched chain) terminal nodes, where terminal nodes are ions connected to no more than one other ion. Chains are required to contain no closed loops along their length. The smallest possible chain is an ion-pair. Examples are shown in Figure. S3.10a, b and f. A ring, or cycle in graph-theoretical terms, is defined as a set of connected nodes that begins and ends at the same node without repeating edges or nodes. While several classes of cycles may exist in a graph, the present analysis focuses on primitive cycles, i.e., cycles that cannot be decomposed into smaller cyclic components and that form the cycle basis of a connected graph. By this definition, the simplest possible rings are 3-membered cycles. Examples are shown in Figure. S3.10c, d and e.

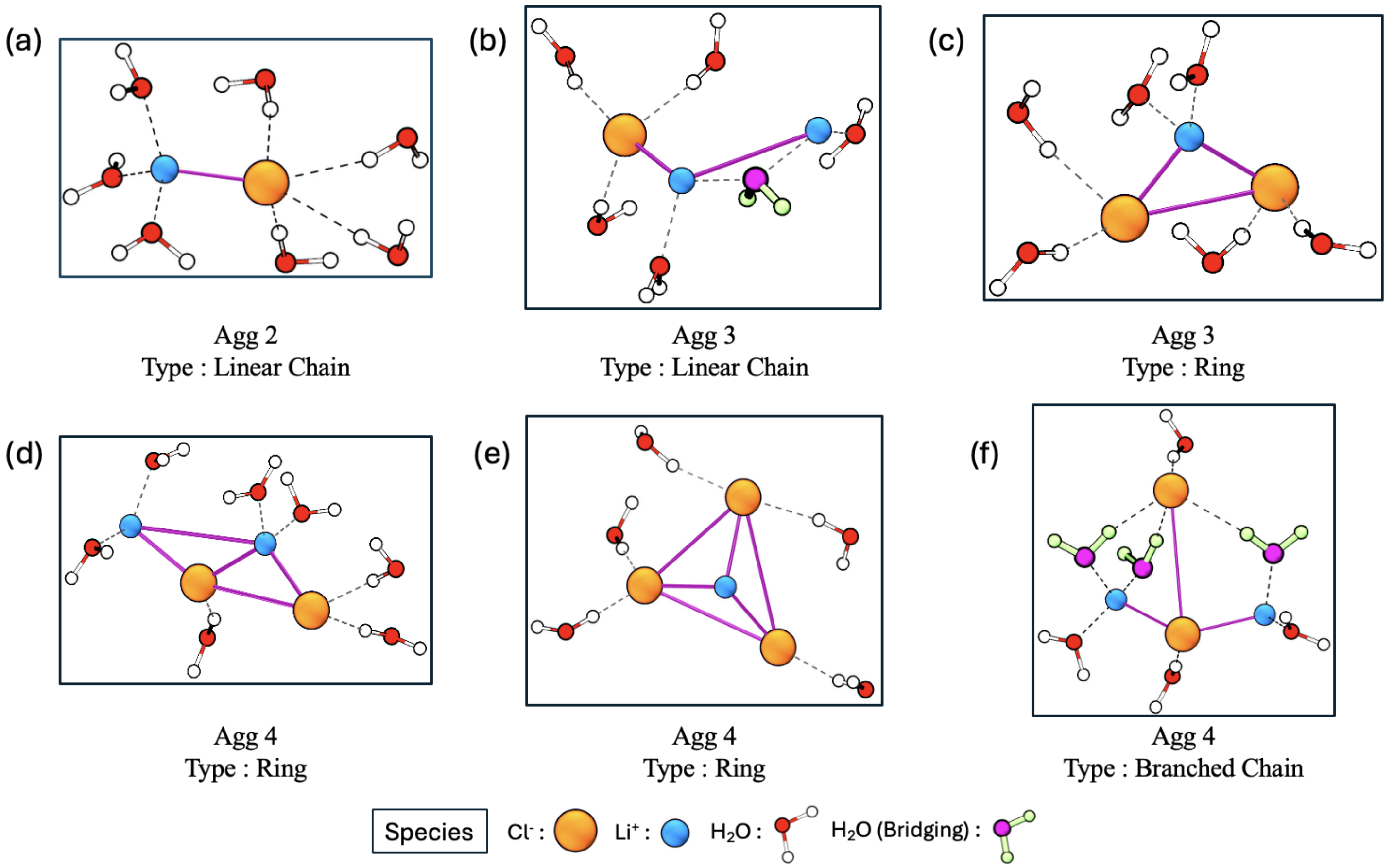

Figure S3.10: *Examples of chains and rings of different cluster sizes : (a) linear chain of size 2, (b) linear chain of size 3, (c) 3-membered ring, (d) two 3-membered rings in a cluster of size 4, (e) three 3-membered rings 3 in a cluster of size 4 and (f) branched chain of size 4.*

S3.6 Laguerre Tessellation

The partial molar volume (V_m) can be constructed from a bottom-up perspective, based on the volumes occupied by individual molecules. The theoretical framework underlying this connection, along with the computational protocol adopted in this work, is described below. Molecular volumes were estimated via tessellation[60,61] using pyvoro, and subsequent data fitting was performed with NumPy. To ensure robust statistical analysis and avoid overfitting, the trajectory dataset was augmented with 30 additional concentrations spanning 0.2-10 M LiCl.

S3.6.A Background

- **System Volume and Partial Molar Volume:**

  Building on the theory from Section S1.2.A, recall that for a binary mixture of LiCl and water at a given molality, the total system volume at a specific concentration (V_{sys}^{conc}) can be expressed as the sum of contributions from each component:

$$V_{sys}^{conc} = n_{LiCl} \left\langle V_{mol}^{conc} \right\rangle_{LiCl} + n_{H_2O} \left\langle V_{mol}^{conc} \right\rangle_{H_2O} \tag{S3.21}$$

  where n_i denotes the number of moles of component i and $\langle V_{mol} \rangle_i$ represents a measure of average microscopic volume associated with that component at the given concentration. By expressing concentrations in terms of molality and applying the variable transformation from Equation S1.8 in the derivative of the PMV definition (Equation S1.3), as outlined in Section S1.2.A, one straightforwardly obtains:

$$V_m^{LiCl} = \left(\frac{1}{n_{H_2O} M_{H_2O}} \right) \frac{\partial}{\partial m} \left[m n_{H_2O} M_{H_2O} \langle V_{\mathrm{mol}}^{\mathrm{m}} \rangle_{LiCl} + n_{H_2O} \langle V_{\mathrm{mol}}^{\mathrm{m}} \rangle_{H_2O} \right] \tag{S3.22}$$

  where M_{H_2O} is expressed in kg/mol which takes out the factor of 1000. Since the moles of water is held constant in the definition of the V_m^{LiCl}, this expression simplifies to:

$$V_m^{LiCl} = \langle V_{\mathrm{mol}}^{\mathrm{m}} \rangle_{LiCl} + m \frac{\partial \langle V_{\mathrm{mol}}^{\mathrm{m}} \rangle_{LiCl}}{\partial m} + \frac{1}{M_{H_2O}} \frac{\partial \langle V_{\mathrm{mol}}^{\mathrm{m}} \rangle_{H_2O}}{\partial m} \tag{S3.23}$$

  This equation provides a direct link between the partial molar volume and the concentration dependence of the microscopic volume contributions of both the solute and the solvent. The three terms on the right-hand side of Equation S3.23 allow for clear physical interpretations. Upon the addition of salt in the form of LiCl formula units, the first term represents the volume that is excluded by the additional inserted ions. The second term, which scales with the molality, accounts for the average change in the local volumes associated with LiCl units already present in the solution; as expected, this contribution vanishes in the limit of infinite dilution. The third term captures the change in the solvent volume induced by the addition of salt. This contribution merits special attention, as the partial molar volume has often been mistakenly interpreted as being associated solely with volume changes of the added component. In fact, the partial molar volume reflects the change in the total volume of the solution upon a change in concentration, and therefore inherently includes contributions from all components of the system[2].

- **Decomposition into Ion Aggregates:**

  The microscopic volume associated with LiCl can be further decomposed into contributions from ionic species and aggregates of different sizes. These include solvated ions (monomers) as well as ion pairs and higher-order clusters. Since large aggregates are rarely observed over most of the concentration range studied here, the expansion can be truncated at a finite cluster size without significant loss of accuracy. Accordingly, the average microscopic volume of LiCl is written as:

$$\langle V^{\mathrm{m}}_{\mathrm{mol}}\rangle_{LiCl} = \sum_{i\in[Agg]} f_i \langle V^{\mathrm{m}}_{\mathrm{mol}}\rangle_i \tag{S3.24}$$

  where the index i runs over the set of cluster sizes considered, f_i denotes the fractional population of clusters of type i, normalized by the number of LiCl formula units taken up to construct the clusters (explained below) and $\langle V_{mol}\rangle_i$ is the time-averaged microscopic volume of such clusters. Substituting this decomposition into the expression for the partial molar volume gives:

$$V^{LiCl}_m = \sum_{i\in[Agg]} \left[f_i \langle V^{\mathrm{m}}_{\mathrm{mol}}\rangle_i + m\left(f_i \frac{\partial \langle V^{\mathrm{m}}_{\mathrm{mol}}\rangle_i}{\partial m} + \langle V^{\mathrm{m}}_{\mathrm{mol}}\rangle_i \frac{\partial f_i}{\partial m}\right)\right] + \frac{1}{M_{H_2O}} \frac{\partial \langle V^{\mathrm{m}}_{\mathrm{mol}}\rangle_{H_2O}}{\partial m} \tag{S3.25}$$

S3.6.B PMV For Clusters

The key components to be obtained via simulations are the average local volumes $\langle V_{mol}\rangle_i$ and the population fractions f_i. The procedure followed in the present work to extract these is outlined below.

- **Population Fractions:**

  The concentration-dependent average cluster populations (F_i) were obtained by counting the total number of clusters for sizes ranging from 1 to 4 in every trajectory frame across the concentration range and subsequently performing a time average of these counts. Although larger aggregates form at the highest concentrations, their populations are negligibly small. Size-1 clusters correspond to fully solvated Li^+ and Cl^- ions (hereafter referred to as Free Li^+ and Free Cl^-), i.e., ions not classified as aggregates according to the cutoff criteria in Table S3.4. Size-2 clusters (denoted Agg 2) comprise all three possible ion pairs: Li^+-Li^+, Cl^--Cl^-, and Li^+-Cl^-. Similarly, size-3 and size-4 clusters (Agg 3 and Agg 4) include all possible triplet and quartet combinations of Li^+ and Cl^-, without distinguishing specific compositions at a given size.

  The population fractions (f_i) can then be obtained from the F_i by dividing them with an estimate of the number of LiCl formula units that are used up by clusters upto a chosen size, i.e., if N_{LiCl} corresponds to the average number of LiCl units consumed in generating a set of populations of cluster up to the size k $F_i, i \in$ [1,2,...k], then :

$$f_i = \frac{F_i}{N_{LiCl}} \tag{S3.26}$$

  If all cluster sizes present in the system, including the largest aggregates, N_{LiCl} reduces to the total number of Li^+ or Cl^- ions in the 1:1 electrolyte LiCl and either of these numbers can act as a proxy for the same. In prac-

tice, however, clusters larger than size four were excluded from the analysis due to their negligible populations. The fraction of clusters of size five and larger constitute only 1.5% of the total LiCl content. However, neglecting this small fraction means that N_{LiCl} can no longer be directly equated to the total number of Li^+ or Cl^-. This issue also pertains to the odd-sized clusters (e.g., Li^+-Li^+-Cl^- and Li^+-Cl^--Cl^+– trimers), which contain unequal numbers of Li^+ and Cl^- ions. As shown in section S3.5.B, the relative populations of these trimers differ, resulting in an asymmetry in the number of Li^+ or Cl^- ions accounted for within the analysed cluster set.

To address these issues, the total number of Li^+ and Cl^- ions accounted for upon truncation of the series in Equation S3.25 were explicitly accumulated at each concentration. For example, a Li^+-Li^+-Cl^- cluster contributes two 2 Li^+ and one Cl^- ion to the respective totals. The effective N_{LiCl} was then defined as the arithmetic mean of the Li^+-based and Cl^--based counts, thereby providing a balanced estimate of the number of ion pairs represented within the truncated cluster population.

Results are shown in Figure S3.11 below.

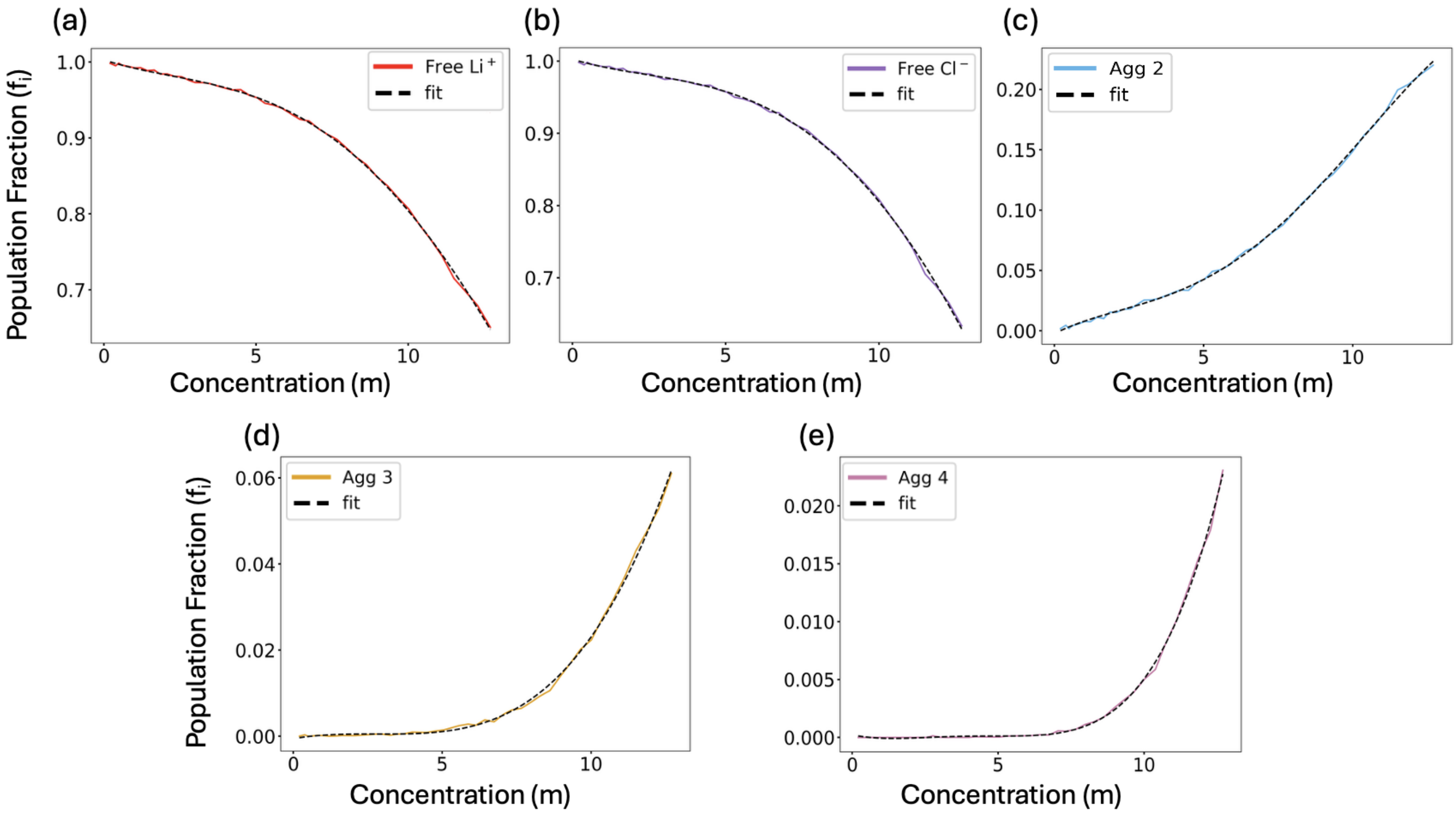

Figure S3.11: *Population Fractions (f_i) for different clusters: (a) Free Li^+, (b) Free Cl^-, (c) Agg 2 (d) Agg 3 and (e) Agg 4.*

In addition, the second derivative of the population fraction for Agg 2 exhibits a maximum at 7.2 M (Figure S3.12), close to the maximum of V_m^{LiCl} at 6.7 M. Such correlation, combined with the discussion in S1.5, indicates the interconnected nature of microscopic structures and thermodynamic properties.

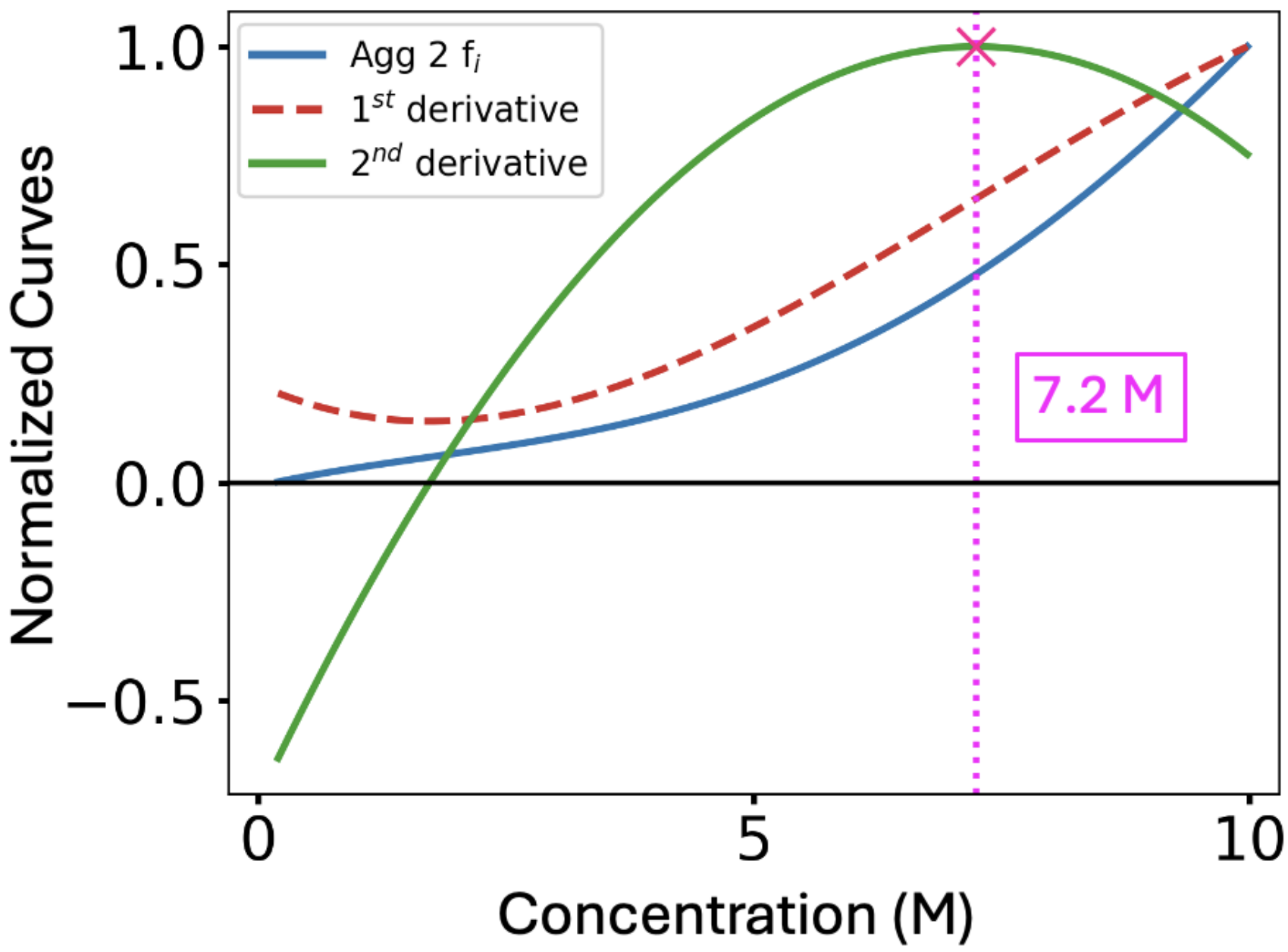

Figure S3.12: *1st and 2nd derivative of the Population Fractions (f_i) of Agg 2 in molarity. Curves are normalized.*

- **Microscopic Volumes and Laguerre-Tessellation:**

Tessellation was chosen as a method to geometrically estimate the local volume of regions in MD simulations. In this approach, space is partitioned into a set of non-overlapping cells, each associated with an individual particle. Laguerre (or radical Voronoi) tessellation was applied to every frame of the trajectories at all concentrations. Unlike standard Voronoi tessellation, Laguerre tessellation partitions space among particles in proportion to the squares of an exclusion radius[62]. A common choice for this radius is half the distance corresponding to the minimum r_{min} of the Lennard-Jones potential, which approximates the Van der Waals interaction between a pair of identical particles[63]. For a species α, r_{min} can be readily obtained from the self Lennard-Jones parameter σ as:

$$r_{min}^{\alpha\alpha} = 2^{1/6}\sigma_{\alpha\alpha} \tag{S3.27}$$

Following the **JC$_{\mathbf{opt}}$** convention, r_{min} were obtained for Li^+, Cl^-, and the oxygen atom of water. These are tabulated below.

Table S3.5: Distance Cutoff for Constructing Graphs

**Particle**	**Li^+**	**Cl^-**	**O**
r_{cut} (nm)	0.065	0.270	0.177

With the $\langle V_{mol} \rangle$ of Li^+ and Cl^- determined across all sampled configurations, cluster identities inherited from the graph analysis can be used to partition the total ionic volume into contributions from clusters belonging to the categories introduced in the previous section. The volume of a given cluster 'C' with a set of ions 'I' is obtained in a simple additive fashion:

$$V_{mol,C} = \sum_{j \in I} V_{mol,j} \tag{S3.28}$$

The time-averaged molar volumes $\langle V_{mol} \rangle$ of the clusters exhibit characteristic trends as a function of concentration, as illustrated in Figure S3.13. The slopes of these curves correspond to the derivatives required in Equation S3.25 to construct the thermodynamic PMV. To obtain analytical expressions for these derivatives, the $\langle V_{mol} \rangle$ – molality data were consistently fitted to fourth-order polynomials. The influence of the order of polynomials used in representations of these trends is investigated in the next section.

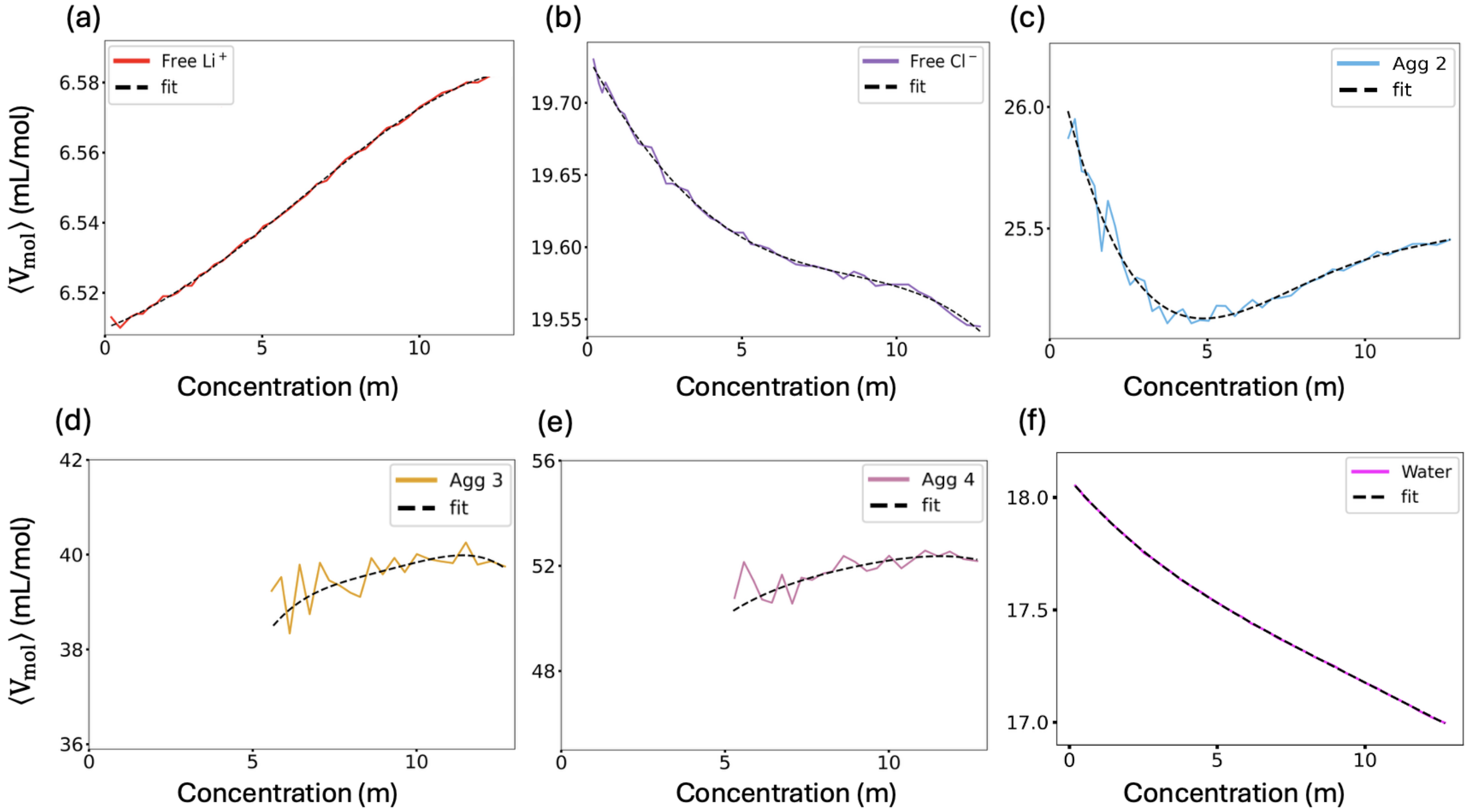

Figure S3.13: *Average Laguerre volumes shown over the concentration ranges used for fitting for different clusters: (a) free Li^+, (b) free Cl^-, (c) Agg 2, (d) Agg 3, (e) Agg 4 and (f) water.*

In addition, for clusters of sizes 2, 3, and 4, the $\langle V_{mol} \rangle$ values at the low-concentration end suffer from poor statistical sampling due to the low probability of aggregate formation, with clusters of size 4 having 0 populations up to 3m concentration, resulting in heteroskedastic behavior (i.e., non-uniform variance across the concentration range). To mitigate the influence of these poorly sampled regions on the fits, the initial 10%, 60%, and 60% of the concentration range were excluded from the polynomial fitting for aggregates of sizes 2, 3, and 4, respectively.

The $\langle V_{mol} \rangle$ curves in Figure S3.13 exhibit a complex dependence on cluster size and concentration. Significantly, single-ion contributions (both $Li^+$and Cl^-) change only by ~1% in opposite directions, consistent with reduced cation solvation and increased anion coordination at higher salt concentrations: Li^+ increasing from 6.51 to 6.58 mL/mol and Cl^- decreasing from 19.73 to 19.55 mL/mol. For ion pairs (Agg 2), $\langle V_{mol} \rangle$ initially decreases, reflecting the reduced solvation inherited from the decreasing trend observed for Cl^- compounded by electrostatic compaction within the pair. However, it increases above 5 M as additional ions approach existing pairs. Aggregates of size 3 and 4, which appear beyond 5 M show a 1-2% increase in

$\langle V_{mol} \rangle$, reflecting the emergence of chain- and ring-like motifs that effectively occupy more volume. On the other hand, the $\langle V_{mol} \rangle$ for water molecules shows a 5% decrease due to the electrostriction.

S3.6.C Sensitivity to the choice of Hyperparameters

The bottom-up molecular framework for computing the PMV involves several methodological choices that may influence the resulting molecular decomposition. Aggregate populations form the basis for the application of Eq. 4 (main text); therefore, it is essential to establish that the PMV decomposition is not an artifact of the graph-construction criteria. The sensitivity of the analysis to variations in the distance cutoffs is examined below.

Beyond graph construction, the PMV framework involves several methodological choices including: (a) the maximum cluster size included in the analysis (Max cluster), (b) the polynomial order used to fit the $\langle V_{mol} \rangle$ trends (poly$_V$), and (c) the polynomial order used to fit the population fractions (poly$_f$).

To optimize these hyperparameters, an empirical error function ($\mathcal{E}$) was constructed using the PMV obtained from the NPT density ($V_m^{(\rho)}$) and molecular volumes ($V_m^{(mol)}$), defined as:

$$\mathcal{E} = \sqrt{\left(\frac{RMSE(V_m^{(\rho)}, V_m^{(mol)})}{\sigma_{V_m}^{Expt}}\right)^2 - \left(\frac{|V_m^{(mol)} - V_m^{(\rho)}|}{\sigma_{Peak}^{Expt}}\right)^2} \tag{S3.29}$$

The first term represents the root-mean-square error between the reference and molecular-model PMVs, while the second term corresponds to the absolute difference in the peak positions predicted by the two models. Each term is normalized by the experimental uncertainty in the respective quantity. This error function balances achieving the overall trends in the PMV relative to the identification of the peak.

- **Geometrical Cutoff for Constructing Graphs:**

  To assess robustness, the distance cutoffs defining graph connectivity were perturbed by ±10% relative to the original values, which correspond to the first minima of the relevant ion RDF (Figures S3.5, S3.6, and S3.3). The three cutoff sets are summarized below:

Table S3.6: Distance Cutoff Sets for Graph Construction Sensitivity Analysis

Cutoff set	Li^+-Li^+ (nm)	Cl^--Cl^+ (nm)	Li^+-Cl^- (nm)
Original	0.412	0.410	0.296
10% increase	0.453	0.451	0.326
10% decrease	0.371	0.369	0.266

The perturbed cutoffs sample different regions of the RDFs, with increased cutoffs extending beyond the first coordination shell and decreased cutoffs remaining near the first RDF maxima.

As expected, cutoff variations redistribute cluster populations : larger cutoffs promote larger aggregates, while smaller cutoffs increase free ions and smaller clusters. The time-averaged populations of the dominant

species at selected concentrations are summarized in Table S3.7 as percentages of the total number of ions. These changes are modest and primarily reflect redistribution between aggregates of different sizes. For example, increasing the cutoff increases the population of ion pairs at intermediate concentration, whereas at higher concentrations the population shifts toward larger aggregates (Agg 3 and Agg 4). These changes are offset by corresponding variations in smaller cluster populations, resulting in only minor changes to the overall cluster distribution.

Table S3.7: Aggregate populations at selected concentrations for different clustering cutoffs.

Aggregate	Concentration (m)	Original Cutoff (%)	Increased Cutoff (%)	Decreased Cutoff (%)
**Free Li**	1.07	49.61	49.32	49.62
	5.72	46.93	43.84	47.18
	12.69	31.09	24.90	31.63
**Free Cl**	1.07	49.61	49.44	49.61
	5.72	47.17	45.48	47.21
	12.69	30.27	25.43	30.57
**Agg 2**	1.07	0.38	0.59	0.38
	5.72	2.72	4.41	2.61
	12.69	10.51	8.69	10.69
**Agg 3**	1.07	0.01	0.02	0.01
	5.72	0.14	0.51	0.12
	12.69	2.92	3.35	2.87
**Agg 4**	1.07	0.00	0.00	0.00
	5.72	0.01	0.07	0.00
	12.69	1.10	1.65	1.03

Proceeding down the pipeline, the PMV contributions were computed from Equation 4 in the main text, for the different cutoff sets and are compared in Figure S3.14. An increased contribution from size 3 and 4 was observed for the larger cutoffs, while the opposite trend was obtained for the decreased cutoffs, consistent with the redistribution of cluster populations. Importantly, only relative contributions were affected, and no qualitative change in the overall PMV decomposition was observed. The robustness of Equation 4 was further confirmed using the error metric defined in Equation S3.29, with comparable values obtained for all cutoffs values (Table S3.8).

These results demonstrate that moderate (±10%) variations in graph-construction criteria produce the expected redistribution of cluster populations but have only small influence on the PMV decomposition. The methodology is therefore shown to be robust with respect to reasonable variations in the choice of the graph-construction cutoff.

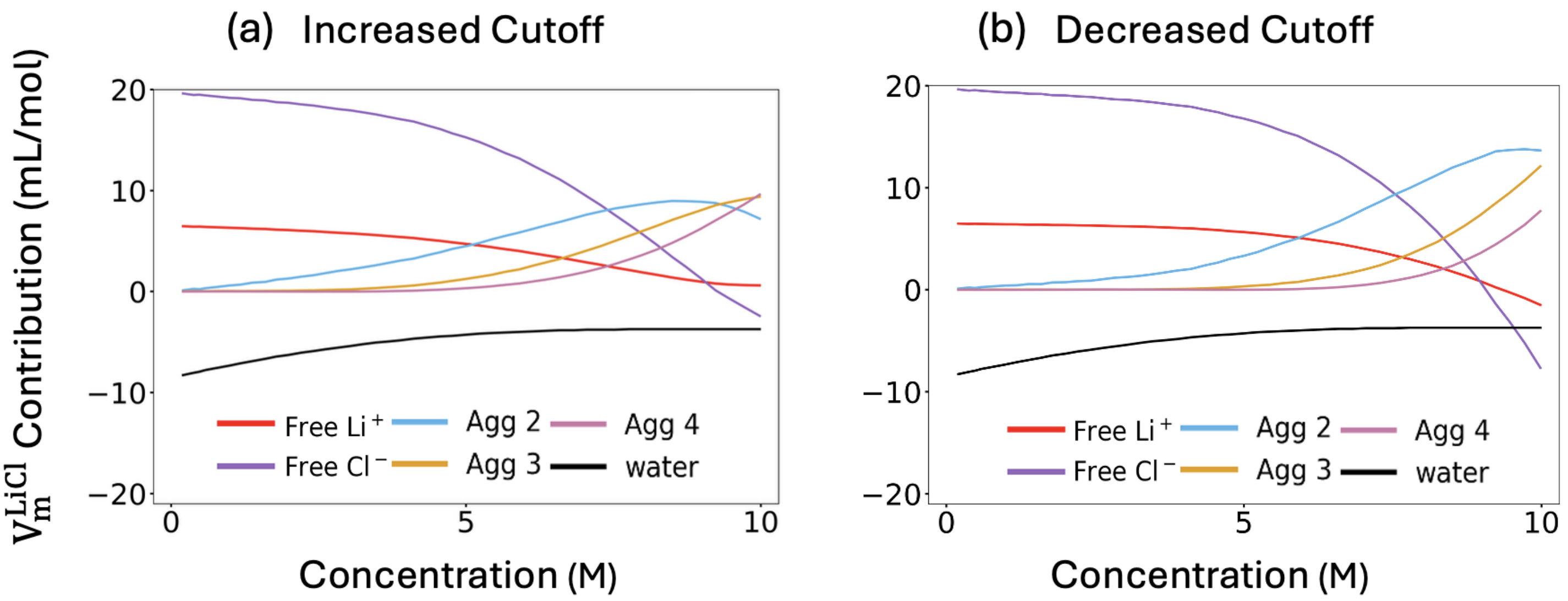

Figure S3.14: *PMV contribution from aggregates of different sizes for graphs generated with (a) 10% larger cutoffs and (b) 10% smaller cutoffs*

Table S3.8: Errors in reproducing PMV trends

Error Metric	Original Cutoff	Increased Cutoff	Decreased Cutoff
$\mathcal{E}$	1.82	1.80	1.92

- **Fit Hyperparameters:**

  The errors (Equation S3.29) from varying the fit hyperparameters are tabulated below. The hyperparameter set, including clusters up to size four, and both the f_i and $\langle V_{mol} \rangle$ fit to polynomials of the 4th order, is chosen as a reference for comparison.

  Table S3.6 indicates that all hyperparameter choices below 3 lead to noticeable deviations, whereas values of 3 or higher yield stable and consistent performance. Accordingly, hyperparameter values ≥ 3 are deemed necessary for a reliable description. For the present work, a uniform choice of 4 was adopted, ensuring internal consistency and aligning with the fourth-order polynomial employed in the virial expansion discussed in Section S1.2.B.

Table S3.9: Hyperparameter sweep and its effect on the error

Parameter Varied	$poly_v$	$poly_f$	Max Cluster	Error
—	4	4	4	1.82
$poly_v$	2	4	4	71.24
	3	4	4	2.89
	5	4	4	2.97
$poly_f$	4	2	4	4.89
	4	3	4	1.89
	4	5	4	2.18
Max Cluster	4	4	2	6.42
	4	4	3	2.10
	4	4	5	1.71

S3.7 Normal Mode Raman Spectra

Analysis of the hydrogen-bond network of water reveals significant distortions in the local coordination environment, particularly in the concentrated solution, where H-bond distributions broaden and shift relative to pure water, indicating a heterogeneous network of competing structural motifs. In an attempt to link this structural heterogeneity with MCR Raman signatures, quantum chemical calculations were performed using the ORCA[64] quantum calculation package on idealized ion-water clusters constructed with imposed geometric constraints, yielding normal-mode Raman intensities as described below.

S3.7.A Evolution of the Hydrogen Bond Network for Water

In response to the development of extended ion networks, the hydrogen-bond network of liquid water undergoes substantial structural reorganization. This is reflected, in part, in changes to the O-O RDF discussed along with Figure S3.8 in Section S3.4.B, where the position of the first maximum shifts to larger separations. To obtain a more complete characterization of the accompanying modifications to the H-bond network, joint probability distributions for O–O distances and O-O-H (acceptor-donor-H) angles were computed for bulk TIP4P/2005 water and for the system at the highest simulated concentration (9.98 M upon equilibration) using the $\mathbf{JC_{opt}}$ parameters. Neighboring water molecules were identified using a geometric cutoff based on the first minimum of the O-O RDF (0.35 nm). For each water molecule in a given configuration, all neighboring waters within this cutoff were analyzed. The angles subtended at the oxygen of the reference water by each of its two hydrogen atoms and the oxygen atom of a neighboring water (i.e., the O-O-H1 and O-O-H2 where the two hydrogens of the reference water are labeled H1 and H2) were collected. The resulting joint distributions were smoothed using a Gaussian kernel density estimator implemented in SciPy. The corresponding results are shown in Figure. S3.15

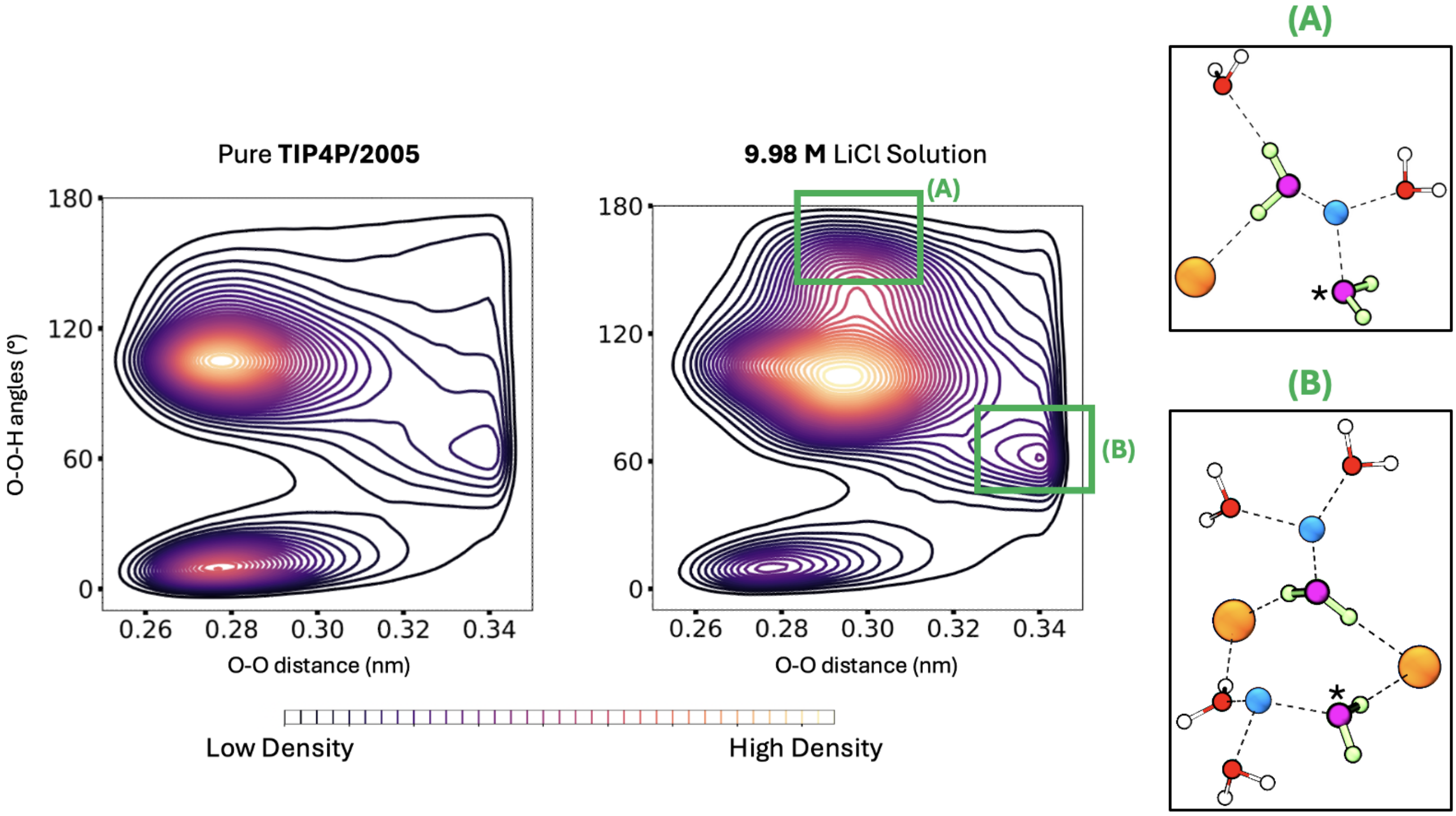

Figure S3.15: *Joint probability distributions of hydrogen-bond parameters in water: acceptor-donor-H angles and O-O distances for pure water with a 9.98 M LiCl solution. Representative structures responsible for the distortions observed in the 9.98 M solution are illustrated on the right. Water molecules exhibiting angles within the highlighted regions of the distributions are shown in purple and green. Among these, those marked with an asterisk (*) are centers of hydrogen-bonding defects within the network.*

The joint probability distribution for pure TIP4P/2005 water exhibits a bimodal character. A sharp peak appears at an O-O separation of 0.28 nm and an O-O-H angle of 30○, consistent with a conventional hydrogen bond according to the Luzar-Chandler[65] definition. A second, broader feature occurs at approximately the same O-O distance but near 110○, arising from geometrically close acceptor–donor pairs within the 0.35 nm cutoff that do not adopt a favorable hydrogen-bonding orientation.

At 9.98 M, the distribution undergoes prominent restructuring. The feature near 0.28 nm and 110○ distorts towards 160○ (Feature A in Fig. S3.15), while a new peak emerges at 0.33 nm and 60○ (Feature B in Figure S3.15). Structural inspection of configurations contributing to these regions reveals water molecules (highlighted in violet and green) simultaneously coordinating either the same Li^+ ion (Feature A) or different Li^+ ions (Feature B), often in the presence of additional nearby counterions. The compact solvation shells of Li^+ impose angular constraints that are uncommon in neat water, driving distortions in the surrounding hydrogen-bond network and often generating locally defective bonding motifs (marked with * in the structural representations), i.e. waters not donating hydrogen bonds to other waters or Cl^- ions, consistent with recent work[66]. These observations not only reaffirm the increasing structural complexity of the ion-water network at high concentration, but also reveal an enhancement of hydrogen-bond defects. This results in a larger fraction of water molecules residing in less tetrahedral environments.

S3.7.B Clusters for Raman Calculations

Model clusters used for the Raman calculations were constructed using several criteria:

- Unless stated otherwise, interspecies distances were chosen based on the position of the first maximum in the corresponding RDF, representing the most probable separation between a given pair of particles. The distances are summarized in Table S3.10. Distance constraints between the heavy atoms are maintained when quantum calculations are performed.
- In all cases, a central water molecule is considered with a complete two-acceptor, two-donor hydrogen-bonding configuration. In the case of the former, a water molecule can accept a hydrogen-bond from a water molecule or be coordinated with a Li^+ ion. For the latter, a water molecule can donate a hydrogen-bond to another water molecule or a Cl^- ion. This molecule serves as the probe water whose vibrational spectra are analyzed, as it most closely represents the local environments encountered in condensed phases. Unless stated otherwise, the geometry around this water molecule is constrained to be tetrahedral, i.e., all angles subtended at the central water are approximately 109°, although the distances to the four neighboring species may differ. For instance, when the central water resides in the first solvation shell of an ion, the ion-water separation is set to the first maximum of the corresponding ion-water RDF, whereas water-water separations are set to the first maximum of the water-water one.
- Whenever a Cl^- ion is included in a cluster, it is considered fully solvated, typically with the central ion included as one of its coordinating water molecules. The geometry around the Cl^- is constrained to be octahedral in all cases. Similarly, any Li^+ generally includes a solvation shell with a tetrahedral geometry enforced.

Table S3.10: Distance between particles in clusters

**Pair**	**Li^+-O**	**Cl^--O**	**Li^+-Cl^-**	**O-O**
Distance (nm)	0.19	0.32	0.23	0.28

S3.7.C Frequency Calibration

All quantum chemical calculations were performed using ORCA with the r^2-SCAN meta-GGA functional[67,68] at the DFT level. The D4-corrected[69] def2-TZVPPD[70,71] basis set was used for Cl^-, O, and H, while Li^+ was treated without the dispersion correction using the def2-TZVPP basis as negligible dispersion is expected in it[72].

The constructed clusters were first subjected to geometry optimization in ORCA, maintaining the interatomic distance constraints described above. Following optimization, the Raman activity A_i for each vibrational mode i was calculated from the derivatives of the system's polarizability along the corresponding normal modes. These Raman activities were then converted to Raman intensities I_i by incorporating the effect of state populations into the general Raman scattering formalism, as expressed in the following Equation[35]:

$$I_i \propto \frac{(\nu_0 - \nu_i)^4 A_i}{\nu_i \left(1 - e^{-\frac{hc\nu_i}{k_B T}}\right)} \tag{S3.30}$$

Here, ν_0 is the excitation frequency of the laser used, which in our case corresponds to 640 nm. The symbols h, c and k_B denote the usual Planck's constant, the speed of light in vacuum, and the Boltzmann constant, respectively. T is the temperature at which the results are evaluated, 298 K in our calculations.

Finally, the obtained spectral frequencies were scaled as is commonplace with DFT functionals, using a factor of 0.968[73]. The scaling reproduces the frequencies of vacuum and bulk water at ~3700 cm^{-1} and ~3400 cm−1 respectively.

References

[1] Gareth James, Daniela Witten, Trevor Hastie, and Robert Tibshirani. *An Introduction to Statistical Learning: with Applications in R, Chapter 6*. Springer, 2013.

[2] Samuel Glasstone. *Thermodynamics for Chemists, Chapter XVIII*. D. Van Nostrand Company, 1947.

[3] Michael J Blandamer and Harald Hiland. Volumetric properties of solutions: A novel method of data analysis yielding partial molar volumes and partial molar expansions of solutes. *Physical Chemistry Chemical Physics*, 1(8):1873–1875, 1999.

[4] William G McMillan Jr and Joseph E Mayer. The statistical thermodynamics of multicomponent systems. *The Journal of Chemical Physics*, 13(7):276–305, 1945.

[5] Jean-Pierre Hansen and Ian Ranald McDonald. *Theory of Simple Liquids: with Applications to Soft Matter, Chapter 3*. Academic Press, 2013.

[6] Frank J. Millero. Molal volumes of electrolytes. *Chemical Reviews*, 71(2):147–176, 1971.

[7] W Heber Green. Ccii. studies on the viscosity and conductivity of some aqueous solutions. part i. solutions of sucrose, hydrogen chloride, and lithium chloride. *Journal of the Chemical Society, Transactions*, 93: 2023–2048, 1908.

[8] W. Nernst and R.A. Lehfeldt. *Theoretical Chemistry from the Standpoint of Avogadro's Rule & Thermodynamics, Chapter VIII*. Macmillan and Company, Limited, 1904.

[9] Gregory Paul Baxter. Changes in volume upon solution in water of the halogen salts of the alkalis. *Journal of the American Chemical Society*, 33(6):922–940, 1911.

[10] V. K. LaMer and T. H. Gronwall. The partial molal volumes of water and salt in solutions of the alkali halides. *The Journal of Physical Chemistry*, 31(3):393–406, 1927.

[11] O. Redlich and P. Rosenfeld. Zur theorie des molvolumens gelöster elektrolyte ii. *Zeitschrift für Elektrochemie und Angewandte Physikalische Chemie*, 37(8-9):705–711, 1931.

[12] Benton B Owen and Stuart R Brinkley Jr. Extrapolation of apparent molal properties of strong electrolytes. *Annals of the New York Academy of Sciences*, 51(4):753–764, 1949.

[13] Pasupati Mukerjee. On ion-solvent interactions. part i. partial molal volumes of ions in aqueous solution1,2. *The Journal of Physical Chemistry*, 65(5):740–744, 1961.

[14] Ralph Albert Horne. *Water and Aqueous Solutions: Structure, Thermodynamics, and Transport Processes, Chapter 13*. Wiley-Interscience, New York, 1972.

[15] D Orme Masson. Xxviii. solute molecular volumes in relation to solvation and ionization. *The London, Edinburgh, and Dublin Philosophical Magazine and Journal of Science*, 8(49):218–235, 1929.

[16] Fred Vaslow. The apparent molal volumes of the alkali metal chlorides in aqueous solution and evidence for salt-induced structure transitions. *The Journal of Physical Chemistry*, 70(7):2286–2294, 1966.

[17] Kenneth S Pitzer and CRC Press. *Activity Coefficients in Electrolyte Solutions*, volume 2. CRC Press Boca Raton, FL, 1991.

[18] BS Krumgalz, R Pogorelsky, Ya A Iosilevskii, A Weiser, and Kenneth S Pitzer. Ion interaction approach for volumetric calculations for solutions of single electrolytes at 25°C. *Journal of Solution Chemistry*, 23(8): 849–875, 1994.

[19] I Yu Klugman. Partial and apparent molar volumes of aqueous solutions of 1:1 electrolytes. *Russian Journal of Electrochemistry*, 38(4):415–420, 2002.

[20] Alexander Y. Payumo, R. Michael Huijon, Deauna D. Mansfield, Laurel M. Belk, Annie K. Bui, Anne E. Knight, and Daryl K. Eggers. Changes in apparent molar water volume and dkp solubility yield insights on the hofmeister effect. *The Journal of Physical Chemistry B*, 115(49):14784–14788, 2011.

[21] Gabriela Horwitz, Paula Y. Steinberg, and Horacio R. Corti. Volumetric and viscosity properties of water-in-salt lithium electrolytes: A comparison with ionic liquids and hydrated molten salts. *The Journal of Chemical Thermodynamics*, 158:106457, 2021.

[22] A.V. Wolf. *Aqueous Solutions and Body Fluids: Their Concentration Properties and Conversion Tables*. Hoeber Medical Division, Harper & Row, 1966.

[23] Otakar Söhnel and Petr Novotný. *Densities of Aqueous Solutions of Inorganic Substances*. Elsevier, 1985.

[24] Victor M. M. Lobo and J. L. Quaresma. *Handbook of electrolyte solutions*. Physical sciences data; 41. Elsevier, Amsterdam, 1989.

[25] Ernesto Vercher, Solange Solsona, M. Isabel Vázquez, and Antoni Martínez-Andreu. Apparent molar volumes of lithium chloride in 1-propanol + water in the temperature range from 288.15 to 318.15 k. *Fluid Phase Equilib.*, 209(1):95–111, 2003.

[26] David R. Lide, editor. *CRC Handbook of Chemistry and Physics, Internet Version*. CRC Press, Boca Raton, FL, 2005.

[27] Gregory Paul Baxter and Curtis Clayton Wallace. Changes in volume upon solution in water of the halogen salts of the alkali metals. ii. *J. Am. Chem. Soc.*, 38:70–105, 1916.

[28] L. Nickels and A. J. Allmand. The electrical conductivities and viscosities at 25°C. of solutions of potassium, sodium, and lithium chlorides, in water and in one-tenth molar hydrochloric acid. *J. Phys. Chem.*, 41:861–872, 1937.

[29] A Ben-Naim. Standard thermodynamics of transfer. uses and misuses. *The Journal of Physical Chemistry*, 82 (7):792–803, 1978.

[30] Manuel R Conde. Properties of aqueous solutions of lithium and calcium chlorides: formulations for use in air conditioning equipment design. *International Journal of Thermal Sciences*, 43(4):367–382, 2004.

[31] JN Pearce and AF Nelson. The vapor pressures of aqueous solutions of lithium nitrate and the activity coefficients of some alkali salts in solutions of high concentration at 25. *Journal of the American Chemical Society*, 54(9):3544–3555, 1932.

[32] Pradeep Perera, Melanie Wyche, Yvette Loethen, and Dor Ben-Amotz. Solute-induced perturbations of solvent-shell molecules observed using multivariate raman curve resolution. *Journal of the American Chemical Society*, 130(14):4576–4577, 2008.

[33] Kathryn Rebecca Fega, Avid Scott Wilcox, and Dor Ben-Amotz. Application of raman multivariate curve resolution to solvation-shell spectroscopy. *Applied Spectroscopy*, 66(3):282–288, 2012.

[34] Huib J Bakker and James L Skinner. Vibrational spectroscopy as a probe of structure and dynamics in liquid water. *Chemical Reviews*, 110(3):1498–1517, 2010.

[35] Johannes Neugebauer, Markus Reiher, Carsten Kind, and Bernd A Hess. Quantum chemical calculation of vibrational spectra of large molecules—Raman and IR spectra for buckminsterfullerene. *Journal of Computational Chemistry*, 23(9):895–910, 2002.

[36] AD Buckingham, JE Del Bene, and SAC McDowell. The hydrogen bond. *Chemical Physics Letters*, 463 (1-3):1–10, 2008.

[37] In Suk Joung and Thomas E Cheatham III. Determination of alkali and halide monovalent ion parameters for use in explicitly solvated biomolecular simulations. *The Journal of Physical Chemistry B*, 112(30):9020–9041, 2008.

[38] Max F Döpke, Othonas A Moultos, and Remco Hartkamp. On the transferability of ion parameters to the tip4p/2005 water model using molecular dynamics simulations. *The Journal of Chemical Physics*, 152(2), 2020.

[39] IM Zeron, JLF Abascal, and C Vega. A force field of Li^+, Na^+, K^+, Mg^{2+}, Ca^{2+}, Cl^-, and SO_4^{2-} in aqueous solution based on the tip4p/2005 water model and scaled charges for the ions. *The Journal of Chemical Physics*, 151(13), 2019.

[40] Jose LF Abascal and Carlos Vega. A general purpose model for the condensed phases of water: Tip4p/2005. *The Journal of Chemical Physics*, 123(23), 2005.

[41] Gerhard Hummer and Jürgen Köfinger. Bayesian ensemble refinement by replica simulations and reweighting. *The Journal of Chemical Physics*, 143(24):243150, 2015.

[42] Jürgen Köfinger, Lukas S. Stelzl, Klaus Reuter, César Allande, Katrin Reichel, and Gerhard Hummer. Efficient ensemble refinement by reweighting. *Journal of Chemical Theory and Computation*, 15(5):3390–3401, 2019.

[43] Andrea Cesari, Sandro Bottaro, Kresten Lindorff-Larsen, Pavel Banáš, Jiří Šponer, and Giovanni Bussi. Fitting corrections to an rna force field using experimental data. *Journal of Chemical Theory and Computation*, 15 (6):3425–3431, 2019.

[44] Thorben Fröhlking, Mattia Bernetti, and Giovanni Bussi. Simultaneous refinement of molecular dynamics ensembles and forward models using experimental data. *The Journal of Chemical Physics*, 158(21), 2023.

[45] Ivan Gilardoni, Thorben Fröhlking, and Giovanni Bussi. Boosting ensemble refinement with transferable force-field corrections: synergistic optimization for molecular simulations. *The Journal of Physical Chemistry Letters*, 15(5):1204–1210, 2024.

[46] Michael P Allen and Dominic J Tildesley. *Computer Simulation of Liquids, Chapter 1*. Oxford University Press, 2017.

[47] Mark James Abraham, Teemu Murtola, Roland Schulz, Szilárd Páll, Jeremy C Smith, Berk Hess, and Erik Lindahl. Gromacs: high performance molecular simulations through multi-level parallelism from laptops to supercomputers. *SoftwareX*, 1:19–25, 2015.

[48] R.W. Hockney and J.W. Eastwood. *Computer Simulation Using Particles*. Advanced book program. McGraw-Hill International Book Company, 1981.

[49] Giovanni Bussi, Davide Donadio, and Michele Parrinello. Canonical sampling through velocity rescaling. *The Journal of Chemical Physics*, 126(1), 2007.

[50] Mattia Bernetti and Giovanni Bussi. Pressure control using stochastic cell rescaling. *The Journal of Chemical Physics*, 153(11), 2020.

[51] Tom Darden, Darrin York, Lee Pedersen, and others. Particle mesh ewald: An N log (N) method for ewald sums in large systems. *Journal of Chemical Physics*, 98(12):10089–10092, 1993.

[52] Berk Hess, Henk Bekker, Herman JC Berendsen, and Johannes GEM Fraaije. Lincs: A linear constraint solver for molecular simulations. *Journal of Computational Chemistry*, 18(12):1463–1472, 1997.

[53] John R Taylor. *An introduction to error analysis: the study of uncertainties in physical measurements, Chapter 9*. MIT Press, 2022.

[54] Hannah O Wood, Hannah M Burnett, Robert AW Dryfe, and Paola Carbone. Stability and structure of the aqueous LiTFSI–LiCl interface. *Faraday Discussions*, 253:212–232, 2024.

[55] Leonardo Perin and Paola Gallo. Phase diagram of aqueous solutions of LiCl: a study of concentration effects on the anomalies of water. *The Journal of Physical Chemistry B*, 127(20):4613–4622, 2023.

[56] Juan L. Aragones, Mauro Rovere, Carlos Vega, and Paola Gallo. Computer simulation study of the structure of LiCl aqueous solutions: Test of non-standard mixing rules in the ion interaction. *The Journal of Physical Chemistry B*, 118(28):7680–7691, 2014.

[57] Jun-Ho Choi and Minhaeng Cho. Ion aggregation in high salt solutions. ii. spectral graph analysis of water hydrogen-bonding network and ion aggregate structures. *The Journal of Chemical Physics*, 141(15), 2014.

[58] Jun-Ho Choi and Minhaeng Cho. Ion aggregation in high salt solutions. iv. graph-theoretical analyses of ion aggregate structure and water hydrogen bonding network. *The Journal of Chemical Physics*, 143(10), 2015.

[59] Jun-Ho Choi, Hyung Ran Choi, Jonggu Jeon, and Minhaeng Cho. Ion aggregation in high salt solutions. vii. the effect of cations on the structures of ion aggregates and water hydrogen-bonding network. *The Journal of Chemical Physics*, 147(15), 2017.

[60] Vladimir P Voloshin, Nikolai N Medvedev, Maximilian N Andrews, R Reddy Burri, Roland Winter, and Alfons Geiger. Volumetric properties of hydrated peptides: Voronoi–delaunay analysis of molecular simulation runs. *The Journal of Physical Chemistry B*, 115(48):14217–14228, 2011.

[61] Vladimir P Voloshin, Alexandra V Kim, Nikolai N Medvedev, Roland Winter, and Alfons Geiger. Calculation of the volumetric characteristics of biomacromolecules in solution by the voronoi–delaunay technique. *Biophysical Chemistry*, 192:1–9, 2014.

[62] Franz Aurenhammer. Power diagrams: properties, algorithms and applications. *SIAM Journal on Computing*, 16(1):78–96, 1987.

[63] Evgenii D Kadtsyn, Vladislav A Nichiporenko, and Nikolai N Medvedev. Volumetric properties of solutions on the perspective of voronoi tessellation. *Journal of Molecular Liquids*, 349:118173, 2022.

[64] Frank Neese. Software update: the orca program system—version 6.0. *Wiley Interdisciplinary Reviews: Computational Molecular Science*, 15(2):e70019, 2025.

[65] Alenka Luzar and David Chandler. Effect of environment on hydrogen bond dynamics in liquid water. *Physical Review Letters*, 76(6):928, 1996.

[66] Man Thi Hong Nguyen, Ondrej Tichacek, Hector Martinez-Seara, Philip E. Mason, and Pavel Jungwirth. Resolving the equal number density puzzle: molecular picture from simulations of LiCl(aq) and NaCl(aq). *The Journal of Physical Chemistry B*, 125(12):3153–3162, 2021.

[67] James W Furness, Aaron D Kaplan, Jinliang Ning, John P Perdew, and Jianwei Sun. Accurate and numerically efficient r2scan meta-generalized gradient approximation. *The Journal of Physical Chemistry Letters*, 11(19): 8208–8215, 2020.

[68] Susi Lehtola, Conrad Steigemann, Micael JT Oliveira, and Miguel AL Marques. Recent developments in libxc—a comprehensive library of functionals for density functional theory. *SoftwareX*, 7:1–5, 2018.

[69] Eike Caldeweyher, Jan-Michael Mewes, Sebastian Ehlert, and Stefan Grimme. Extension and evaluation of the d4 london-dispersion model for periodic systems. *Physical Chemistry Chemical Physics*, 22(16): 8499–8512, 2020.

[70] Florian Weigend and Reinhart Ahlrichs. Balanced basis sets of split valence, triple zeta valence and quadruple zeta valence quality for h to rn: design and assessment of accuracy. *Physical Chemistry Chemical Physics*, 7 (18):3297–3305, 2005.

[71] Dmitrij Rappoport and Filipp Furche. Property-optimized gaussian basis sets for molecular response calculations. *The Journal of Chemical Physics*, 133(13), 2010.

[72] Vojtech Kostal, Philip E Mason, Hector Martinez-Seara, and Pavel Jungwirth. Common cations are not polarizable: effects of dispersion correction on hydration structures from ab initio molecular dynamics. *The Journal of Physical Chemistry Letters*, 14(19):4403–4408, 2023.

[73] Denis S Tikhonov, Igor Gordiy, Danila A Iakovlev, Alisa A Gorislav, Mikhail A Kalinin, Sergei A Nikolenko, Ksenia M Malaskeevich, Karina Yureva, Nikita A Matsokin, and Melanie Schnell. Harmonic scale factors of fundamental transitions for dispersion-corrected quantum chemical methods. *ChemPhysChem*, 25(23): e202400547, 2024.
